# Identifying the HLA DRB1-DQB1 molecules and predicting epitopes associated with high-risk HPV infection clearance and redetection

**DOI:** 10.1038/s41598-020-64268-x

**Published:** 2020-04-29

**Authors:** Luisa Del Río-Ospina, Milena Camargo, Sara C. Soto-De León, Ricardo Sánchez, Darwin A. Moreno-Pérez, Manuel E. Patarroyo, Manuel A. Patarroyo

**Affiliations:** 1 Molecular Biology and Immunology Department, Fundación Instituto de Inmunología de Colombia, Carrera 50 # 26-20, Bogotá, Colombia; 2 Clinical Research Group, Instituto Nacional de Cancerología, Calle 1 # 9-85, Bogotá, Colombia; 3 PhD Programme in Biomedical and Biological Sciences, Universidad del Rosario, Carrera 24 # 63C-69, Bogotá, Colombia; 4 Animal Science Faculty, Universidad de Ciencias Aplicadas y Ambientales (U.D.C.A), Calle 222 # 55-37, Bogotá, Colombia; 5 Faculty of Medicine, Universidad Nacional de Colombia, Carrera 45 # 26-85, Bogotá, Colombia; 6 School of Medicine and Health Sciences, Universidad del Rosario, Carrera 24 # 63C-69, Bogotá, Colombia

**Keywords:** Gynaecological cancer, Immunogenetics, Risk factors

## Abstract

Several determining factors are involved in HPV infection outcomes; human leukocyte antigen (HLA) polymorphisms have been described as related factors. This study has ascertained the effect of genetic variation on HLA-DRB1 and DQB1 genes on HPV-16/-18/-31/-33/-45 and -58 clearance and redetection in Colombian women. PCR and qPCR were used for viral identification and the Illumina MiSeq system was used for HLA-typing of cervical samples (n = 276). Survival models were adjusted for identifying alleles/haplotypes related to HPV clearance/redetection; L1/L2 protein-epitope binding to MHC-II molecules was also predicted. Significant associations suggested effects favouring or hampering clearance/redetection events depending on the viral type involved in infection, e.g. just DRB1*12:01:01G favoured HPV-16 (coeff: 4.8) and HPV-45 clearance (coeff: 12.65) whilst HPV-18 (coeff: 2E-15), HPV-31 (coeff: 8E-17) and HPV-58 hindered elimination (coeff: 1E-14). An effect was only observed for some alelles when configured as haplotypes, e.g. DRB1*04:07:01G (having the greatest frequency in the target population) was associated with DQB1*02:01:1G or *03:02:03. Epitope prediction identified 23 clearance-related peptides and 29 were redetection-related; eight might have been related to HPV-16/-18 and -58 persistence and one to HPV-18 elimination. HLA allele/haplotype relationship with the course of HPV infection (clearance/redetection) depended on the infecting HPV type, in line with the specific viral epitopes displayed.

## Introduction

Human papilloma virus (HPV) is the most common sexually-transmitted viral infection, having around 291 million infections annually^1–3^. The causal relationship between persistent high-risk types of human papillomavirus (HR-HPV) infections and the development of cervical lesions progressing to cervical cancer (CC) has been extensively demonstrated^4,5^. Many infections (around 90%) are eliminated during an average period of 2 years; however, some of them are latent as non-productive infection is limited to the epithelium’s base layer and they do not become detected^6,7^. A complex interaction between viral and host factors is responsible for CC’s clinical course and development^8,9^. Host immunological and genetic factors play an essential role in HPV infection outcome^10,11^. Human leukocyte antigen (HLA) system alleles and haplotypes have been reported as being responsible for antigen presentation, recognition of infected cells and HPV elimination^12,13^.

Some HLA alleles and haplotypes have been described as being associated with CC, such as DRB1*07:02, DRB1*13:01, DQA1*01:03 and DQB1*06:03^14–16^. However, most reports contain inconsistencies given the differences in the populations being analysed, the typing techniques, the outcomes considered and the methodological design^11,15,17–19^. Few longitudinal studies have evaluated these molecules’ association with HPV’s natural history of infection (considering them as being generic infections) and just HPV-16 and -18 have been considered at type-specific level^19–22^.

This study was aimed at identifying HLA-*DRB1* and *DQB1* alleles related to the clearance and redetection of the 6 HPV types having the greatest distribution in Colombia (HR-HPV-16, -18, -31, -33, -45 and -58) in a cohort of Colombian women using next generation sequencing (NGS) for HLA typing and quantitative PCR assay for viral detection. L1 and L2 protein peptides fitting into alleles were analysed for predicting which of them might have been related to infection events regarding each viral type. Such information is relevant to understanding specific infections’ natural history and the genetic factors modulating them. The results should prove useful in identifying inmunological biomarkers enabling establishing HPV infection suceptibility and its clinical course.

## Results

### Selecting the study population

Two hundred and seventy-six women were considered eligible for this study; 12 were excluded as complete information was missing (lack of information regarding HPV or HLA typing). Cohort follow-up lasted 32.3 months; 206 women were visited four times and 59 women a fifth time during the follow-up period. Table 1 describes the target population and its sociodemographic characteristics. Regarding type-specific detection, HPV-16 had the greatest prevalence, followed by HPV-18; however, variations regarding specific type distribution were found during follow-up (shown in Supplementary Fig. S1).

**Table 1:** Baseline sociodemographic characteristics, risk factors and cervical findings regarding the study population.

	Characteristic	n	%	95%CI	
				Lower	Upper
Age, years (median, IQR)		43 (34-50)			
Origin (n = 266)	Chaparral	7	2.63	0.70	4.57
	Bogotá	82	30.83	25.24	36.41
	Girardot	187	66.54	60.83	72.25
Ethnicity (n = 266)	Afro-descendant	5	1.88	0.24	3.52
	Mestizo	259	97.37	95.43	99.30
	Indigenous	2	0.75	−0.29	1.80
Smoker (n = 266)	No	200	75.19	69.61	80.04
	Yes	66	24.81	19.96	30.39
Age on first intercourse, years (n = 266)	≤18 years	154	57.89	51.84	63.72
	>18 years	112	42.11	36.28	48.16
Lifetime amount of sexual partners (n = 260)	1	116	44.62	38.47	50.88
	2	88	33.85	28.12	39.95
	≥3	56	21.54	16.70	27.04
Contraceptive method (n = 248)	None	96	38.71	32.61	44.81
	Barrier	42	16.94	12.24	21.64
	Surgery	81	32.66	26.78	38.54
	Hormonal	29	11.69	7.67	15.72
Births (n = 257)	None	10	3.89	1.51	6.27
	1-2	127	49.42	43.26	55.57
	3-4	104	40.47	34.43	46.51
	>4	16	6.23	3.25	9.20
Abortions (n = 200)	None	104	52.00	45.02	58.98
	1	72	36.00	29.29	42.71
	≥2	24	12.00	7.46	16.54
STD (n = 256)	No	205	80.08	75.15	85.00
	Yes	51	19.92	15.00	24.85
Cytological findings (n = 264)	Negative	241	91.29	87.86	94.71
	ASCUS	10	3.79	1.47	6.11
	LSIL	13	4.92	2.30	7.55

IQR: interquartile range, 95%CI: 95% confidence interval, STD: sexually transmitted disease, ASCUS: atypical squamous cells of undetermined significance, LSIL: low-grade squamous intraepithelial lesion.

### HLA *DRB1* and *DQB1* allele and haplotype frequencies and the clinical course of HR-HPV infection

Supplementary Tables S1 and S2 give HLA *DRB1* and *DQB1* allele and haplotype frequencies. The results regarding type-specific infection clearance for 219 women out of the 276 included in this study have already been reported^23^; Table 2 gives the data for the women included in this study (n = 264). Supplementary Tables S3 and S4 give sociodemographic variable distribution, risk factors and cytopathology result concerning the three outcomes considered (clearance, persistence and redetection).

**Table 2:** Clearance and redetection rates for the 6 HR-HPV types.

Viral type	Clearance						Redetection					
	Events^a^		Median time	Rate	95%CI		Events^a^		Median time	Rate	95%CI	
	n	%			Lower	Upper	n	%			Lower	Upper
HPV-16	119	36.28	7.70	5.77	4.82	6.91	105	50.24	21.00	5.62	4.64	6.80
HPV-18	66	22.00	NA	2.32	1.82	2.95	69	29.24	26.87	6.27	4.95	7.93
HPV-31	74	23.42	NA	2.45	1.95	3.07	73	31.74	11.53	6.31	5.02	7.94
HPV-33	46	28.22	7.00	6.99	5.24	9.33	9	12.50	NA	1.73	0.90	3.32
HPV-45	96	33.68	9.10	5.13	4.20	6.27	70	32.26	12.40	4.94	3.91	6.24
HPV-58	100	37.88	13.57	4.30	3.53	5.23	40	16.60	NA	3.06	2.25	4.18

HPV: human papillomavirus, 95%CI: 95% confidence interval.

Rates per 100 people/month.

^a^Percentage clearance and redetection events identified in the total amount of infections and those proving negative during follow-up.

n/a: not applicable, right-censored data.

**Figure 1:**
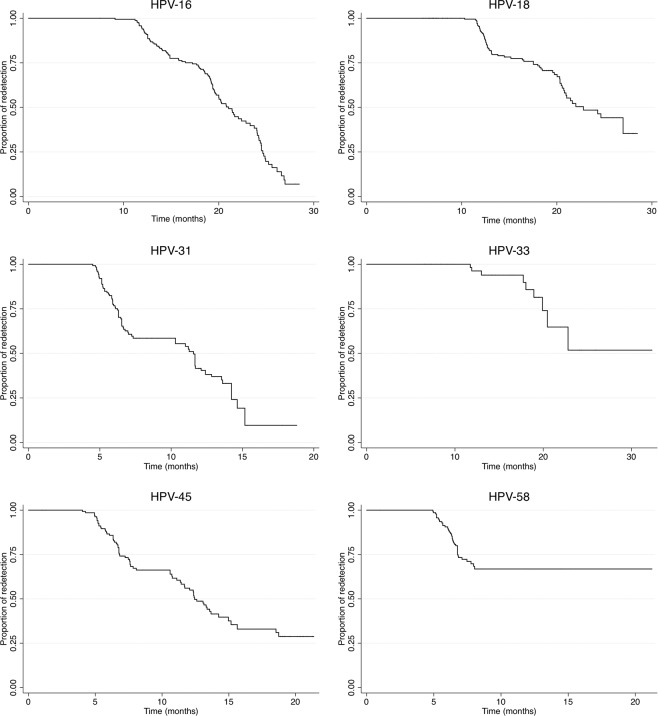
Kaplan Meier curves for redetecting the six HR-HPV types. Abbreviations: HR-HPV: high-risk human papillomavirus.

Evaluating time-related redetection percentage revealed that most viral types (HPV-18, -33, -45 and -58) became positive again after 5 months’ non-detection (Fig. 1). There was a lower percentage of redetection of positive cases at the end of follow-up for HPV-16 (6.95%) and HPV-31 (9.64%) compared to a higher non-redetection percentage for HPV-58 (66.8%) and HPV-33 (51.82%) at the end of follow-up (Fig. 1). The highest clearance rate was observed for HPV-33 (6.99) whilst HPV-18 (6.27) and HPV-31 (6.31) had the highest redetection rates (Table 2).

### Identifying HLA alleles and haplotypes related to HR-HPV infection’s clinical course

Calculating multicollinearity between the variables (evaluated by variance inflation factor (VIF) and tolerance) revealed no collinearity between the variables included in the model. Cox multivariate and log-normal models were thus adjusted for identifying alleles and haplotypes related to infection clearance/persistence (Supplementary Tables S5 to S8) and redetection (Supplementary Tables S9 to S11) for each HPV type evaluated.

Alleles/haplotypes having associations favouring clearance/redetection were found (considered in the model as having greater and earlier probability of occurrence) as well as hindering associations (considered as lower probability and later occurrence).

**Figure 2:**
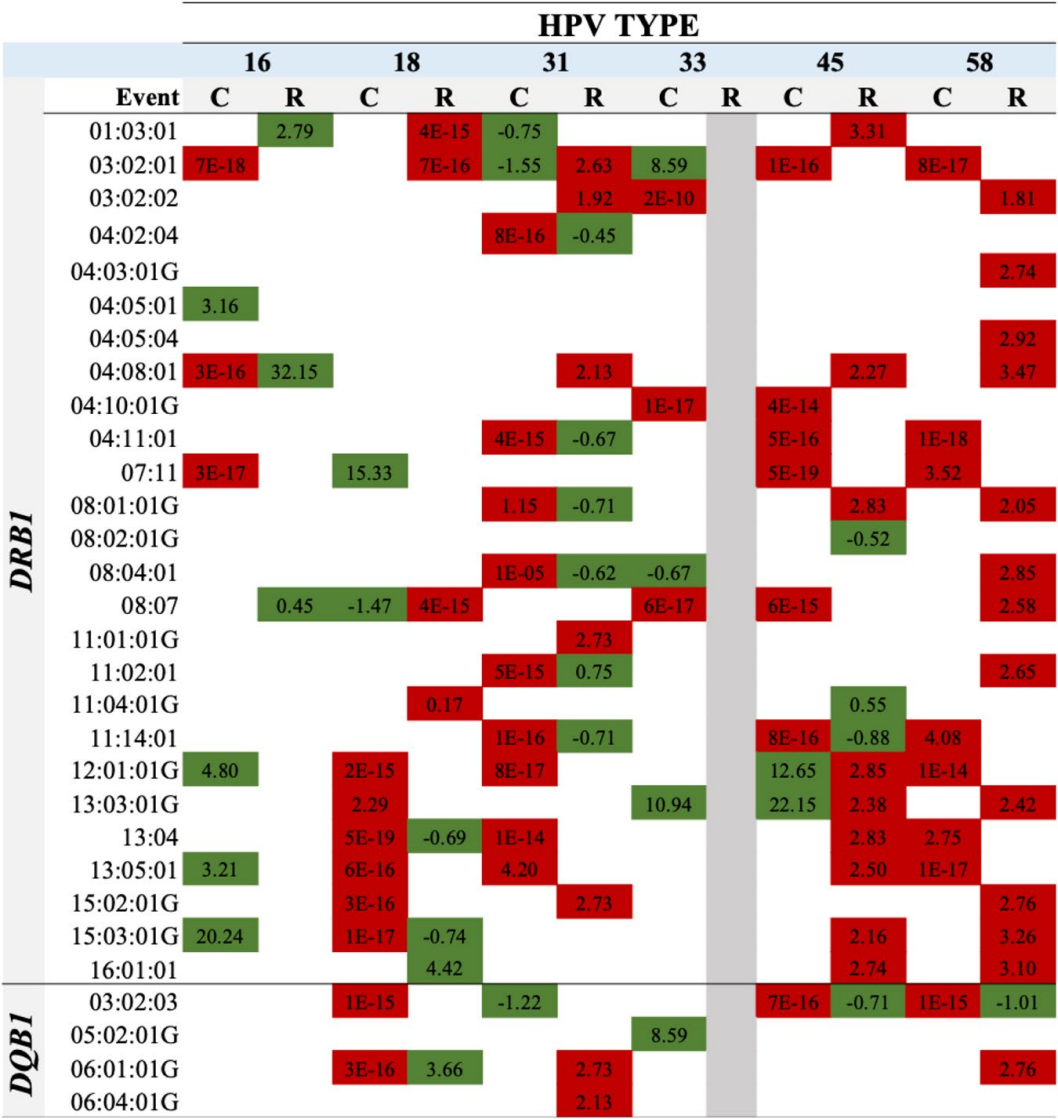
Schematic representation of *DRB1* and *DQB1* alleles associated with clearance or redetection for each of the 6 HR-HPV types. Associations shown in green represent alleles favouring a particular event (clearance or redetection), whilst associations shown in red represent alleles hindering an event (clearance or redetection). Abbreviations: C: clearance; R: redetection).

Sixty-three *DRB1* and nine *DQB1* allele associations with HR-HPV infection clearance were found (Supplementary Tables S5 and S6) of which 47 for *DRB1* (represented by 20 alleles) and 6 for *DQB1* (represented by 3 alleles) continued being significant after *p* values (*p* ≤ 0.001) had been corrected (Fig. 2 and Supplementary Table S12). Fifteen associations were identified favouring clearance (greater probability or earlier occurrence) and 38 hindering it (lower probability or later occurrence) (Fig. 2).

The HPV-33 model could not be adjusted for infection redetection due to the few (n = 9) events found for this viral type in the study population (Supplementary Table S9); 88 associations were found for the remaining viral types (Supplementary Table S9 and S13). Forty-five associations continued being significant for *DRB1* (23 alleles) and five for *DQB1* (3 alleles) after the *p* values had been corrected (*p* ≤ 0.001), 17 of these associations favouring infection redetection and 33 hindering it (Fig. 2 and Supplementary Table S13).

**Figure 3:**
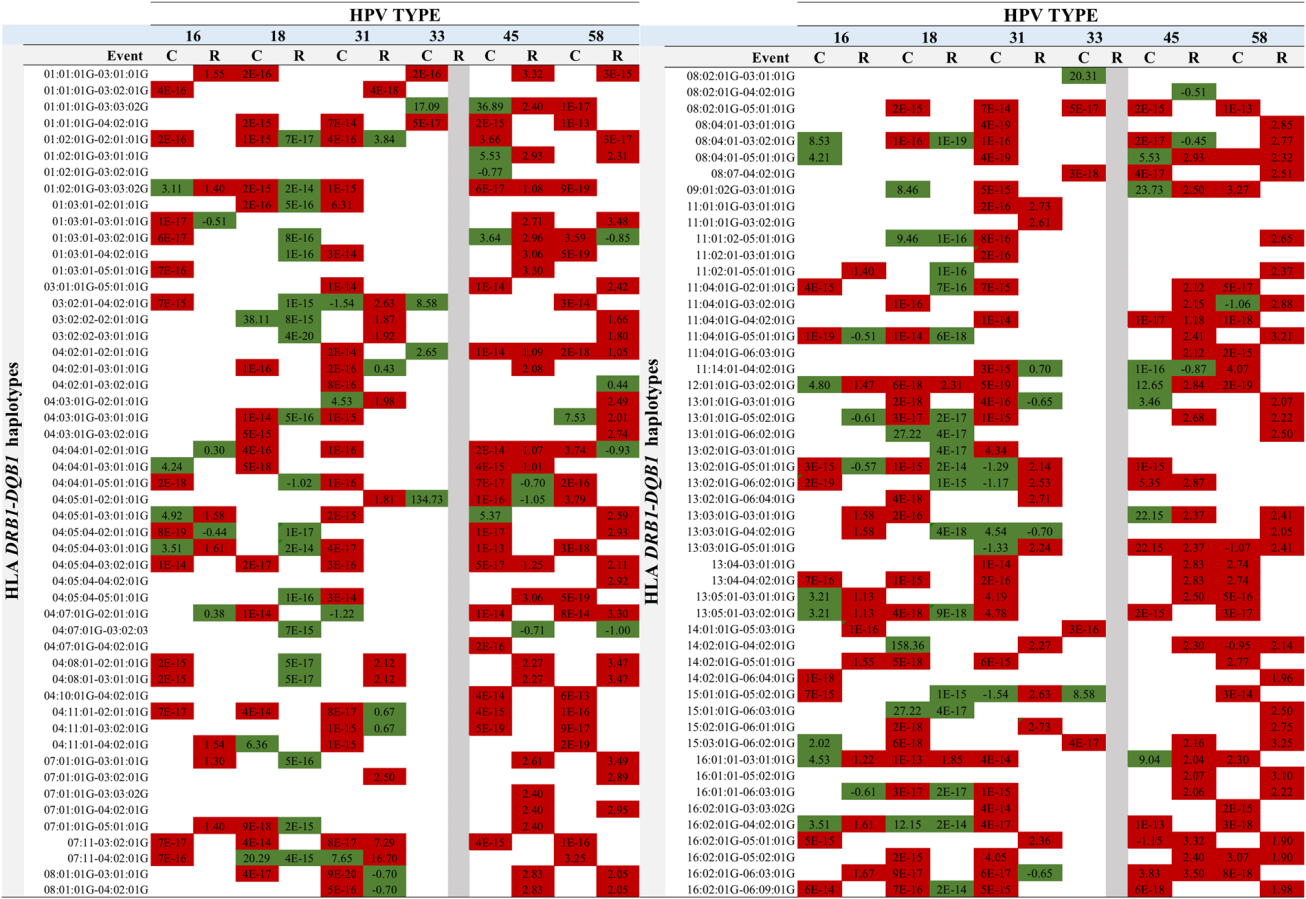
Schematic representation of haplotypes associated with clearance or redetection for each of the 6 HR-HPV types. Associations shown in green represent alleles favouring clearance or redetection whilst associations shown in red represent alleles hindering clearance or redetection. Abbreviations: C: clearance; R: redetection).

Two hundred and ninety-five associations were identified for 144 *DRB1*-*DQB1* haplotypes (Supplementary Tables S7, S8 and S14); 233 associations (represented in 88 haplotypes) continued being significant (Fig. 3 and Supplementary Table S14) following Bonferroni correction (*p* ≤ 0.001). Twenty-four of these were positively associated with the probability of infection clearance whilst 63 had a negative effect, i.e. favouring HPV infection persistence (Fig. 3). Regarding redetection, 264 associations were found, 197 of them (represented by 94 haplotypes) continued being significant following *p* value correction (*p* ≤ 0.001) (Fig. 3 and Supplementary Table S15), 64 favouring redetection and 133 hindering it.

Some alleles and haplotypes were found to be related to a single HR-HPV type and others were associated with more than just one HR-HPV type. Concerning the latter, allele/haplotype associations were found which were consistent amongst the different HPV types (i.e. in the same sense, favouring or hindering a particular event), e.g. DRB1* 04:11:01 favouring HPV-31, -45 and -58 redetection. Different associations were found depending on HR-HPV type (favouring and hindering a particular event), e.g. DRB1*03:02:01 was associated with favouring HPV-31 and -33 clearance, whilst HPV-16, -45 and -58 hindered clearance (Fig. 2).

The association found for some haplotypes agreed with that found for the alleles constituting them (e.g. DRB1*15:02:01G-DQB1*06:01:01G) (Figs. 2 and 3). Haplotypes having significant associations were found where *DRB1* and *DQB1* constituting them had no independent associations (e.g. DRB1*04:05:01-DQB1*02:01:01G, for HPV-33, -45 and -58 for clearance) as was modification of the effect when alleles were associated and not associated independently (e.g. DRB1*11:04:01G-DQB1*02:01:01G for redetection) (Figs. 2 and 3).

Allele/haplotype associations were found having the same sense as the event (clearance and/or redetection) for the same HPV type, i.e. an allele/haplotype was associated with hindering clearance of an HPV type and also associated with a greater probability of redetection of the same viral type. For example, DRB1*11:02:01 was associated with a lower probability of HPV-31 clearance and earlier redetection of the same viral type. DRB1*03:02:01-DQB1*04:02:01 was associated with earlier HPV-31 clearance and its later redetection (Figs. 2 and 3).

### Clearance and redetection event-related peptides

It was predicted that 23 L1 and L2 capsid protein peptides from different viral types had strong binding to different HLA-II molecules (Table 3 and Supplementary Tables S16 to S19). Some peptides could have been related to the same infection outcome amongst viral types (peptide binding to DRB1*01:03, DRB1*07:11 (HPV-16 and -45) and DRB1*13:04 (HPV-18, -31 and -58)). Others could have explained the differences regarding an event due to being specific for each virus (peptides for DRB1*04:08, DRB1*07:11, DRB1*13:03 and DRB1*15:03 all from HPV-18) (Table 3 and Supplementary Tables S16 to S19).

**Table 3:** HR-HPV L1 and L2 proteins’ MHC-II binding epitopes related to clearance/persistence events.

Event	Allele	HR-HPV	Outcome	Peptides per protein^a^	%Rank
Clearance/Persistence	DRB1*01:03	31	Earlier occurrence	L1-VTRTNI**YYHAGSARL**LTVGH L1-FPLGRK**FLLQAGYRA**RPKFK L1-ITLSADIMT**YIHSMNPAI**LE L2-DPDFLDI**IALHRPALT**SRRN	0.08 0.60 1.30 3.50
		45	Greater probability	L1-YVSRTSI**FYHAGSSRL**LTVG L1-QYPLGRK**FLVQAGLRR**RPTI L1-TAEVMS**YIHSMNSSI**LENWN L2- SDFMDI**IRLHRPALS**SRRGT	0.15 3.00 3.00 1.10
	DRB1*04:08	16	Lower probability	L1-TGFGAMD**FTTLQANK**SEVPL L1-QFPLGRK**FLLQAGLKA**KPKF L2-EIPMDT**FIVSTNPNT**VTSST L2-MLRKRRKRLPY**FFSDVSLAA** L2-AETGGH**FTLSSSTIS**THNYE	4.00 4.00 1.20 1.60 4.50
		18	Greater probability	L1-GHYIIL**FLRNVNVFP**IFLQM L2-TTSFAFFKYSPTISSASSYS	3.00 0.17
	DRB1*07:11	16	Lower probability	L2-QQVKVVDPA**FVTTPTKLI**TY	3.00
		18	Greater probability	L2-TTSFAF**FKYSPTISS**ASSYS	4.50
		45	Lower probability	L2- QQ**VRVSTSRFL**TRPSSLVTF	4.00
	DRB1*13:03	18	Lower probability	L1-GHYIIL**FLRNVNVFPI**FLQM	4.50
		33	Greater probability	—	—
		45	Greater probability	—	—
		58	Earlier occurrence	—	—
	DRB1*13:04	18	Lower probability	L2-YLWP**LYYFIPKKR**KRVPYFF	1.70
		31	Lower probability	L2-YLHPSYYM**LKRRRKRVS**YFF	0.40
		58	Later occurrence	L2-FMLHPSYF**ILRRRRKRF**PYF	0.40
	DRB1*15:03	16	Greater probability	L1-QFPLGRK**FLLQAGLKA**KPKF	3.00
				L1-FYLRREQM**FVRHLFNRA**GAV	4.00
		18	Lower probability	L1-GHYIILF**LRNVNVFPI**FLQM	3.00
				L1-FYHAGSFR**LLTVGNPYF**RVP L2-DSDFMDI**IRLHRPALT**SRRG L2-RSTTSFA**FFKYSPTIS**SASS	4.50 1.70 3.00

^a^The peptide core for binding to an allele is shown in bold.

Interestingly, some peptides could have favoured or hindered a clearance event depending on the allele presenting it, e.g. HPV-16 L1-QFPLGRKFLLQAGLKAKPKF presented by DRB1*04:08 and DRB1*15:03, HPV-18 L1-GHYIILFLRNVNVFPIFLQM presented by DRB1*04:08, DRB1*13:03 and DRB1*15:03 and HPV-18 L2-TTSFAFFKYSPTISSASSYS presented by DRB1*04:08, DRB1*07:11 and DRB1*15:03.

Twenty-nine L1 and L2 capsid protein peptides were found to be related to redetection. Some peptides could have been related to the same event amongst viral types (e.g. those presented by DRB1*01:03 (HPV-18 and HPV-45), DRB1*13:04, DRB1*15:02 (HPV-31 and HPV-58), whilst peptides presented by the other alleles could explain the differences found regarding events (Table 4 and Supplementary Tables S16 to S19).

**Table 4:** HR-HPV L1 and L2 proteins’ MHC-II binding epitopes related to redetection events.

Event	Allele	HR-HPV	Outcome	Peptides per protein^a^	%Rank
Redetection	DRB1*01:03	16	Greater probability	L1-YLRREQM**FVRHLFNRA**GAVG	4.50
		18	Lower probability	L2-SDFMDI**IRLHRPALT**SRRGT	1.40
		45	Later occurrence	L2-SDFMDI**IRLHRPALS**SRRGT	1.10
	DRB1*03:02	18	Lower probability	—	—
		31	Later occurrence	—	—
		45	Earlier occurrence	L2-QIGGRVH**FYHDISPIA**ATEE	4.50
		58	Later occurrence	—	—
	DRB1*04:02	18	Greater probability	L1-GHYIILF**LRNVNVFPI**FLQM L1-IFYHAGSFR**LLTVGNPYF**RV L2-SFAF**FKYSPTISS**ASSYSNV L2-SDFMDI**IRLHRPALT**SRRGT	4.00 4.50 1.70 4.00
		58	Later occurrence	L1-FPLGRK**FLLQSGLKA**KPRLK	4.00
	DRB1*04:08	16	Greater probability	L1-TGFGAMD**FTTLQANKS**EVPL L2-AETGGH**FTLSSSTIS**THNYE	4.00 4.50
		31	Later occurrence	—	—
		45	Later occurrence	—	—
		58	Later occurrence	—	—
	DRB1*10:01	18	Lower probability	L2-TTSFAF**FKYSPTISS**ASSYS	0.25
		45	Earlier occurrence	L1-KVSAYQ**YRVFRVALP**DPNKF L1-RLLTVGNP**YFRVVPSGA**GNK L1-LTAEVMS**YIHSMNSSI**LENW	1.40 3.00 4.50
	DRB1*12:01	18	Greater probability	—	—
		31	Earlier occurrence	—	—
		45	Later occurrence	L1-NIIYGHGI**IIFLKNVNV**FPI	4.00
		58	Earlier occurrence	—	—
	DRB1*13:03	18	Greater probability	L1-GHYIILF**LRNVNVFPI**FLQM	4.50
		45	Later occurrence	—	—
		58	Later occurrence	—	—
	DRB1*13:04	18	Earlier occurrence	L2- YLWP**LYYFIPKKR**KRVPYFF	1.70
		31	Earlier occurrence	L2- YLHPSYYM**LKRRRKRVS**YFF	0.40
		45	Later occurrence	—	—
		58	Earlier occurrence	L2- FMLHPSYF**ILRRRRKRF**PYF	0.40
	DRB1*15:02	18	Greater probability	L1-CGHYIILF**LRNVNVFPI**FLQ L2- STTSFAF**FKYSPTISS**ASSY L2- IGARVH**FYHDISPIA**PSPEY	1.10 4.00 4.00
		31	Later occurrence	L2-SIFVDGGD**FYLHPSYYM**LKR	3.50
		58	Later occurrence	L2- TIIVDGAD**FMLHPSYFI**LRR	2.00
	DQB1*03:02	16	Greater probability	L1-YVARTNI**YYHAGTSRL**LAVG	1.20
				L1-TLEDT**YRFVTSQAI**ACQKHT	1.60
		45	Earlier occurrence	L1-YVSRTSI**FYHAGSSRL**LTVG	0.50
				L1-TTSLVDT**YRFVQSVAV**TCQK	1.60
		58	Earlier occurrence	L1-YVSRTSI**YYYAGSSRL**LAVG	0.70
				L1-ASLQDT**YRFVTSQAI**TCQKT	2.50
	DQB1*05:02	16	Earlier occurrence	L1-YVARTNI**YYHAGTSRL**LAVG	1.20
				L1-TLEDT**YRFVTSQAI**ACQKHT	1.60
		18	Greater probability	L1-RTSI**FYHAGSFRL**LTVGNPY	0.90
				L1-TTSLVDT**YRFVQSVAI**TCQK	2.00
	DQB1*06:01	18	Greater probability	—	—
		31	Later occurrence	L1-QFPLGRK**FLLQAGYRA**RPKF	0.70
		58	Later occurrence	L1-QFPLGRK**FLLQSGLKA**KPRL	0.70
	DQB1*04:02	18	Greater probability	L2-TTSFAF**FKYSPTISS**ASSYS	1.80
		45	Later occurrence	L2-SDFMDI**IRLHRPALS**SRRGT	4.50

^a^The peptide core for binding to an allele is shown in bold.

Some viral peptides could have favoured or hindered a redetection event depending on the allele presenting them, e.g. L1-GHYIILFLRNVNVFPIFLQM favouring a greater probability of HPV-18 redetection when presented by DRB1*04:02, DRB1*13:03 and DRB1*15:02, like HPV-16 L1-YVARTNIYYHAGTSRLLAVG and L1-TLEDTYRFVTSQAIACQKHT when presented by DQB1*03:02 and DQB1*05:02. L1-FPLGRKFLLQSGLKAKPRLK presented by DRB1*04:02 and DQB1*06:01 could have been related to a later HPV-58 viral type occurrence. Contrarily, HPV-18 L2-SDFMDIIRLHRPALTSRRGT could have been changing the redetection event when presented by DRB1*01:03 or DRB1*04:02 and L2-SFAFFKYSPTISSASSYSNV when presented by DRB1*04:02, DRB1*10:01 or DRB1*15:02.

Particularly interesting was the observation that responses to clearance and redetection were related when some MHC class II peptides were presented. For example, HPV-16 L1-TGFGAMD**FTTLQANK**SEVPL presented by DRB1*04:08 and HPV-18 L1-GHYIIL**FLRNVNVFPI**FLQM presented by DRB1*13:03 were related to a lower probability of clearance and greater probability of redetection. The same occurred when HPV-16 L2- AETGGHFTLSSSTISTHNYE and HPV-18 L2-YLWPLYYFIPKKRKRVPYFF and HPV-31 L2-YLHPSYYMLKRRRKRVSYFF were presented by DRB1*04:08 and HPV-58 L2-FMLHPSYFILRRRRKRFPYF by DRB1*13:04.

## Discussion

Differences were seen throughout follow-up regarding infection patterns for all 6 HR-HPV types, this being consistent with previous studies^23–25^. However, differences amongst studies have been reported, mainly due to variations concerning persistence, clearance and redetection^26^, inclusion of prevalent and incident infection patterns^24^ and target population characteristics (host risk factors).

This study’s findings regarding infection clearance correlated with those of a prior cohort study carried out with part of the population in this study^23^. Differences concerning redetection rates were seen to depend on the HR-HPV type; the percentages and rates reported for every viral type were similar to those reported previously^27,28^. However, redetection events could not be differentiated between new infection and/or latent infection due to study design^29^.

Previous studies evaluating HPV generic redetection have reported cumulative percentages of up to 23.9% regarding new viral identification^29,30^. Cumulative post-clearance redetection in this study, including all types of HR-HPV analysed, was 7.42% and cumulative redetection following non-detection was 22.10% (Table 2). However, comparing redetection and clearance rates amongst studies is difficult regarding the different designs and definitions used regarding follow-up duration and the type of cohort^28,31^.

Significant associations between HLA class II alleles/haplotypes and outcomes regarding infection (clearance/persistence, redetection) were found to be positive (greater probability or earlier occurrence of an event) or negative (lower probability or later occurrence of an event) (Figs. 2 and 3). The alleles/haplotypes favourably associated with viral clearance and hindering redetection could have been related to a lower risk of CC (given the lower risk of infection and viral persistence), whilst associations hindering clearance and favouring redetection could have been related to a greater risk of CC (greater risk of infection and persistence). Previous cohort results (regarding just infection by HPV-16 and -18) would seem to support such inferences and the results presented here (Supplementary Tables S12 to S15)^19–22^.

An immune response against HPV plays an essential role in determining such infections’ clinical course and the natural history of CC. Women having alleles/haplotypes negatively associated with clearance in this study would probably have had a lower viral peptide presentation for activating an immune response, thus favouring persistence and thereby increasing the risk of developing CC^32,33^. Immunoinformatics led to identifying viral peptides which could be considered factors favouring viral persistence in a host since they were related to a lower probability/later occurrence of a clearance event and the greater probability/earlier occurrence of redetection (Tables 3 and 4).

Alleles/haplotypes favouring redetection (greater probability or earlier occurrence), could have favoured HPV replication due to lower antigen presentation capability favouring the replication of such latent infections in a host, so that only when the amount of copies exceeded the detection threshold could they have been identified and diagnosed^29^. Epitope prediction thus contributes to identifying key host factors involved in the response to infection and could therefore be considered for designing therapeutic tools for HPV infection control (Tables 3 and 4).

Specific associations with HPV type were found for most HLA-*DRB1* and *DQB1* alleles and haplotypes identified in the study population, highlighting differences in the relationship with the clinical course of such infection in a host (Figs. 2 and 3). Some of these associations have been reported for other populations in which generic HPV has been considered or just HPV-16 or -18 (Supplementary Tables S12 and S14). Some alleles (DRB1*13:01 and DQB1*06:03) and haplotypes (DRB1*03:01:01G-DQB1*02:01:01G and DRB1*13:01 01G-DQB1*06:03:01G) previously reported as associated were not significant for this study’s population^11,34–36^.

Other associations (e.g. DRB1*01:03, DRB1*04:02, DRB1*04:08, DRB1*04:10, DRB1*04:11 DRB1*08:04, DRB1*11:14, DRB1*13:03, DRB1*13:04 and DRB1*13:05 alleles and DRB1*03:02:01-DQB1*04:02:01G and DRB1*04:02:01-DQB1*03:02:01G haplotypes) were not consistent with that reported previously or constituted new findings. This was especially true regarding redetection, this being the first study to consider this outcome (Figs. 2 and 3; Supplementary Tables S12 to S15)^19^. The forgoing could be explained by the greater variability obtained for HLA molecules when using the NGS technique, including viral types different to HPV-16 and -18, each population’s particular characteristics, interaction with other host and viral factors^11^ and differences in studies’ methodological designs and the outcomes considered (i.e. pre-neoplastic lesions and CC)^11,15,17–19^.

A joint effect was observed in this population which could be considered as the average of each allele’s individual effect or as a secondary effect regarding possible allele interaction^17,22^. The forgoing considers that some haplotypes were found having consistent effects with that found for the alleles constituting them, haplotypes with alleles not associated independently and haplotypes formed by an associated allele and another non-associated allele.

Future studies should consider additional variables such as changes regarding the amount of sexual partners through follow-up and changes in viral load, thereby broadening understanding of outcomes and supporting the conclusions, mainly regarding this study’s redetection events. Although biological assays are required to support the bioinformatics findings described here, this has been the first study predicting peptides which could favour or hinder viral persistence (in viral types different to HPV-16 and -18) and demonstrating their potential usefulness as therapeutic anti-HPV peptide vaccines.

This study has thus reported, for the first time, alleles and haplotypes (typed by NGS) associated with clearance/persistence and redetection events, as well as L1 and L2 epitopes from the six most frequently occurring HPV types which are responsible for around 85% of CC cases. The information obtained through this study provides relevant knowledge for understanding the genetic component in the immune response against HR-HPV types and the natural history of CC. The associations described here enable constructing the bases for future studies aimed at evaluating the impact and effectiveness of anti-HPV vaccines and treatments (current and future), considering each population’s genetic particularities.

## Materials and Methods

### Study population

The study’s target population consisted of a cohort of women who had been participating in a Fundación Instituto de Inmunología de Colombia (FIDIC) multicentre study evaluating 6 HR-HPV types’ persistence, clearance and reinfection^23^. The women had been attending hospitals in the Colombian cities of Bogotá, Girardot and Chaparral between January 2007 and March 2010; they voluntarily participated in the study and declared that they had not changed their place of residence in at least two years after the start of the study. Detailed information regarding the study population and the procedures related to the 6 HR-HPV types’ detection and quantification have been described in previous publications^5,23^.

Women having had at least three follow-ups (6 ± 3-month intervals) and real-time PCR viral identification results were included in the study. Women whose cervical samples did not have the minimum amount of DNA and/or required quality for HLA-*DRB1* and *DQB* typing were excluded from the analysis (Supplementary Fig. S2A). All the methods were performed in accordance with the Helsinki declaration and the Colombian Ministry of Health and Social Protection guidelines, as approved by the Universidad Nacional de Colombia’s Faculty of Medicine’s Research Ethics Committee (resolution 004-067-18). All the women who agreed to participate in the study signed an informed consent form.

### DNA extraction

A QIAamp 96 DNA Blood Kit (QIAGEN, Hilden, Germany) was used for extracting genomic DNA (gDNA) from the 276 aliquots containing the cervical samples (stored in FIDIC’s DNA bank), following the manufacturer’s recommendations and specifications. The cervical samples’ gDNA quality for HLA typing was determined as >10 ng/μL concentration and ≥1.8/2.0 absorbance ratio at 260/280 nm.

### Detecting and quantifying human papillomavirus

Conventional PCR was used for detecting six HR-HPV types (HR-HPV-16, -18, - 31, - 33, -45 and -58) and qPCR was used for quantifying the viral load, following previously described procedures^23,37,38^. The human *β-globin* gene was amplified by PCR for evaluating DNA integrity, using GH20/PC04 primers. This was followed by using two sets of generic primers targeting a region of the *L1* gene for the generic detection of HPV (GP5 +/GP6 + and MY09/MY11) (Supplementary Table S20)^37,38^.

Samples proving positive by at least one generic primer set were typed using primers targeting a region located on the *E5*, *E6* and *E7* genes; these were specific for HR-HPV-16, -18, - 31, - 33, -45 or -58 (Supplementary Table S20). All amplification products were visualised on 2% agarose gels. Synthetic genes from early HPV-18, -31, -45 and -58 regions of interest and samples proving positive and confirmed by Sanger sequencing for HPV-16 and -33 were used as positive controls. DNA-free water was used as negative control^37,38^.

Real-time PCR detection was used for determining the amount of viral copies and the CFX96 Touch qPCR detection system was used for analysis; the primers and TaqMan probes used here are described in Supplementary Table S21. 1:10 (10^11^–10^6^) serial dilutions were obtained from plasmid DNA (known concentration) from each viral type and the *HMBS* gene for making the calibration curve. The human *HMBS* gene was used for validating DNA integrity and determining the amount of viral copies per cell. The samples were analysed for the aforementioned six HR-HPV types, involving absolute (total HPV copies) and relative quantification (HPV copies /cell = HPV copies /(*HMBS*/2 copies)) of each type’s load. Six dilutions of plasmid DNA were included for each type and included as controls for *HMBS* in each analysis along with a negative NTC control (no template control)^5,23^.

### HLA typing

Illumina MiSeq (San Diego, CA, USA; Histogenetics, Ossining, NY, USA) was used for typing HLA-DRB1 and DQB1 l molecules from exons 2 and 3 from every *loci* (3x resolution) from good quality gDNA samples^39,40^. The IPD-IMGT/HLA database (https://www.ebi.ac.uk/ipd/imgt/hla), published in January 2018 (3.31.0), was used for assigning alleles. WHO Nomenclature Committee for Factors of the HLA System guidelines were followed when reporting alleles, using National Marrow Donor Program (NMDP) codes and G codes for ambiguous alleles (i.e. those having an identical nucleotide sequence at the antigen recognition site in exon 2)^41^.

### Statistical analysis

Frequencies and percentages were used for qualitative variables and measures of central tendency (median and mean) for quantitative ones, along with their measures of dispersion (interquartile ranges (IQR) and standard deviation (SD).

An expectation-maximisation (EM) algorithm was used for obtaining HLA- *DRB1* and *DQB1* allele and haplotype frequencies. Persistence was considered as being the detection of the same type of HPV in at least two consecutive visits, whilst clearance was determined as no viral detection in two consecutive samples after a positive detection when analysing infection by each of the 6 HPV types (-16, -18, -31, -33, -45 and -58). Redetection was taken as being positive for the same viral type following its prior non-detection (regardless of infection clearance) (Supplementary Fig. S2B)^23,42,43^. As some women had had more than one positive HPV-related event during follow-up, specific HR-HPV type infections were taken as the unit of analysis (i.e. not the women).

Cox-proportional hazard and accelerated failure time (AFT) models were adjusted when the assumption of proportionality was not met to evaluate allele and HLA-*DRB1* and *DQB1* relationship with type-specific infection (clearance/redetection) outcome^44^. Multicollinearity between the variables included in the models was also evaluated by VIF and tolerance.

Every AFT models’ goodness of fit was evaluated in line with Akaike information criterion (AIC) and Bayesian information criterion (BIC), selecting the model having the best fit (lowest AIC and BIC values)^45^. Hazard ratios (HR) and time ratios (TR) were reported, depending on the model used^46,47^. Independent models were run for each HPV type. TR values less than 1 denoted an earlier occurrence of an event, whilst values greater than 1 indicated a later occurrence of such event^46,47^.

A *p* < 0.200 value in univariate analysis was taken when selecting the independent variables for adjusting the models and the change in the crude estimator when added to the models. Origin, age, the amount of sexual partners, a background of abortion, coinfection (i.e. infection by 2 or more HR-HPV types) and absolute viral load (categorised as low: ≤9.99E + 5, middle: from 1.00E + 6 to 9.99E + 9 and high: ≥1.00E + 10^23^) were taken as independent variables.

The Bonferroni method was used for correcting each model’s raw *p* values, considering the multiple alleles and haplotypes identified in the study population and that there was no *a priori* hypothesis concerning the associated alleles/haplotypes^20,48^. Two-tailed tests were used for hypotheses testing (0.05 significance) and STATA14 software was used for the aforementioned analysis.

### Predicting epitopes for HLA-II alleles

The Immune Epitope Database (IEDB) was used for predicting binding peptides (20 aa long) for every HLA-II allele^49^, using the Technical University of Denmark’s Systems Biology Department’s Center for Biological Sequence Analysis’ NetMHCIIpan 3.2 server prediction method^50^. Peptides predicted to have a strong binding threshold (<5% Rank) were sought manually by aligning each HR-HPV type’s complete L1 or L2 capsid protein reference sequences (GenBank codes: HPV16-L1: ANA05539.1; HPV18-L1: AGG40789.1; HPV31-L1: AEI60965.1; HPV33-L1: AEI61181.1; HPV45-L1: ABP99855.1; HPV58-L1: BBA20221.1; HPV16-L2: AFP44645.1; HPV18-L2: AAP20600.1; HPV31-L2: AIG59270.1; HPV33-L2: AMY16574.1; HPV45-L2: ALV85694.1, HPV58-L2: AMY16537.1). This was aimed at finding similar or different regions and determining possible explanations for the associations found between HLA-II and HR-HPV. Peptide sequences coinciding with the secretion signal sequence were not considered for analysis.

## Supplementary information

Supplementary information.

## Data Availability

The datasets produced and/or analysed during this study are available from the corresponding author on reasonable request.
