# Supplementary material for: Identifying the HLA DRB1-DQB1 molecules and predicting epitopes associated with high-risk HPV infection clearance and redetection
Source: Sci Rep. 2020 Apr 29;10:7306. doi: 10.1038/s41598-020-64268-x (PMC7190668; doi:10.1038/s41598-020-64268-x)
Supplement: Supplementary file 1 — Supplementary information. [file 41598_2020_64268_MOESM1_ESM.pdf]

Identifying HLA DRB1-DQB1 molecules and predicting epitopes associated with high-risk HPV infection clearance and redetection

Luisa Del Río-Ospina[1,2†], Milena Camargo[1,3,4†], Sara C. Soto-De León[1†], Ricardo Sánchez[5], Darwin A. Moreno-Pérez[1,6], Manuel E. Patarroyo[1,5], Manuel A. Patarroyo[1,6*]

[1]Molecular Biology and Immunology Department, Fundación Instituto de Inmunología de Colombia, Carrera 50 # 26-20, Bogotá, Colombia

[2]Clinical Research Group, Instituto Nacional de Cancerología, Calle 1 # 9-85, Bogotá, Colombia

[3]PhD Programme in Biomedical and Biological Sciences, Universidad del Rosario, Carrera 24 # 63C-69, Bogotá, Colombia

[4]Animal Science Faculty, Universidad de Ciencias Aplicadas y Ambientales (U.D.C.A), Calle 222 # 55-37, Bogotá, Colombia

[5]Faculty of Medicine, Universidad Nacional de Colombia, Carrera 45 # 26-85, Bogotá, Colombia

[6]School of Medicine and Health Sciences, Universidad del Rosario, Carrera 24 # 63C-69, Bogotá, Colombia

[†]These authors contributed equally to this work

Correspondence: Manuel Alfonso Patarroyo M.D., Dr.Sc.
e-mail: mapatarr.fidic@gmail.com
Telephone number: +57-1-3244672 ext 141

Supplementary Figure legends

Supplementary Fig. S1. Prevalence of HPV types regarding Follow-up. Abbreviations; 1st FU: first follow-up; 2nd FU: second follow-up; 3rd FU: third follow-up.

Supplementary Fig. S2. Study design flowchart. (A) Patient flow in this study. (B) Schematic representation of persistence, clearance and redetection outcomes defined in this study (BioRender (@biorender.com) was used for making this diagram). Abbreviations; 1st FU: first follow-up; 2nd FU: second follow-up; 3rd FU: third follow-up.

[a] Regarding sociodemographic information, clinical data or results of HPV detection or HLA typing.

Supplementary Fig. S1.

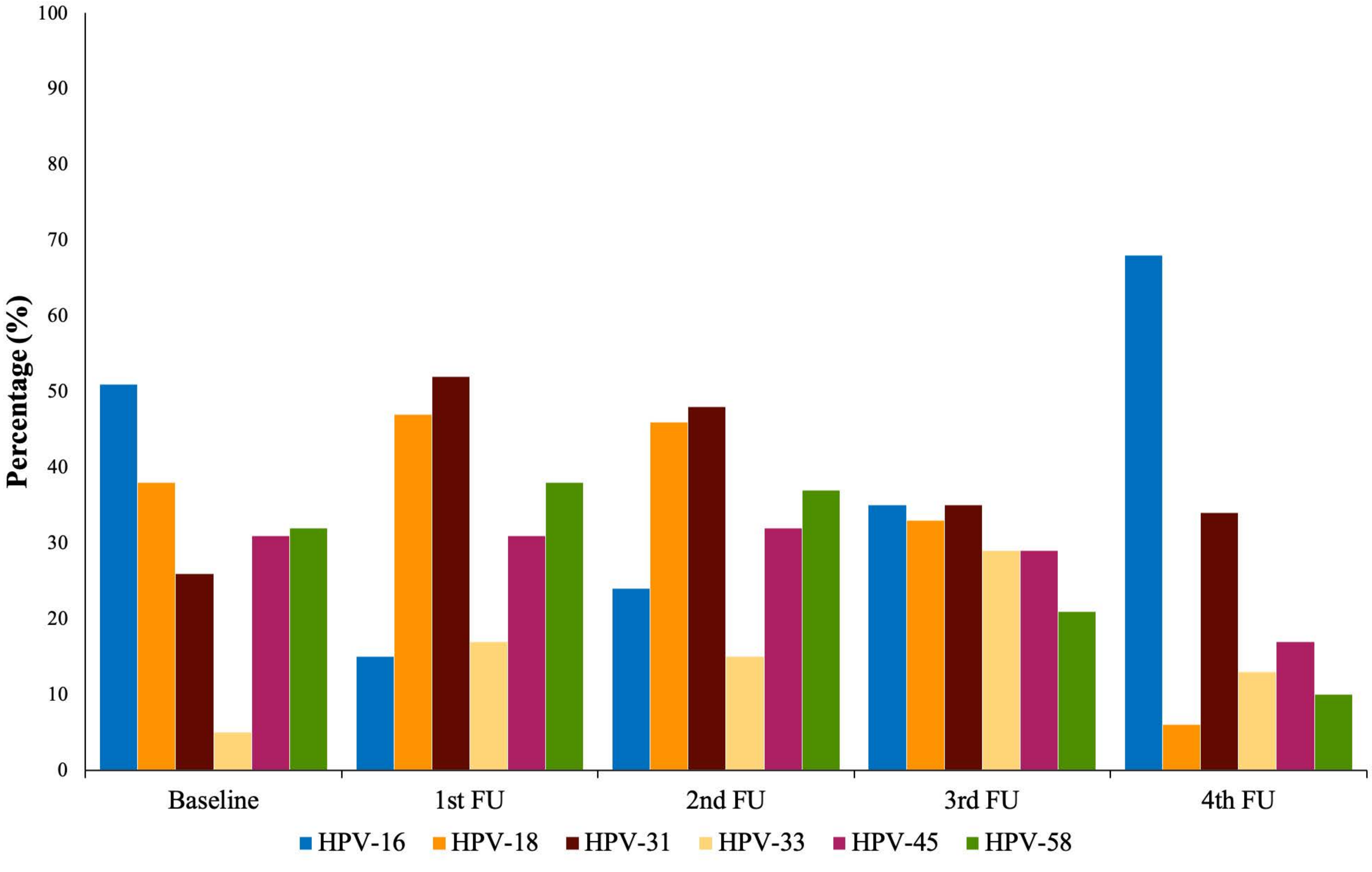

Supplementary Fig. S2.

A

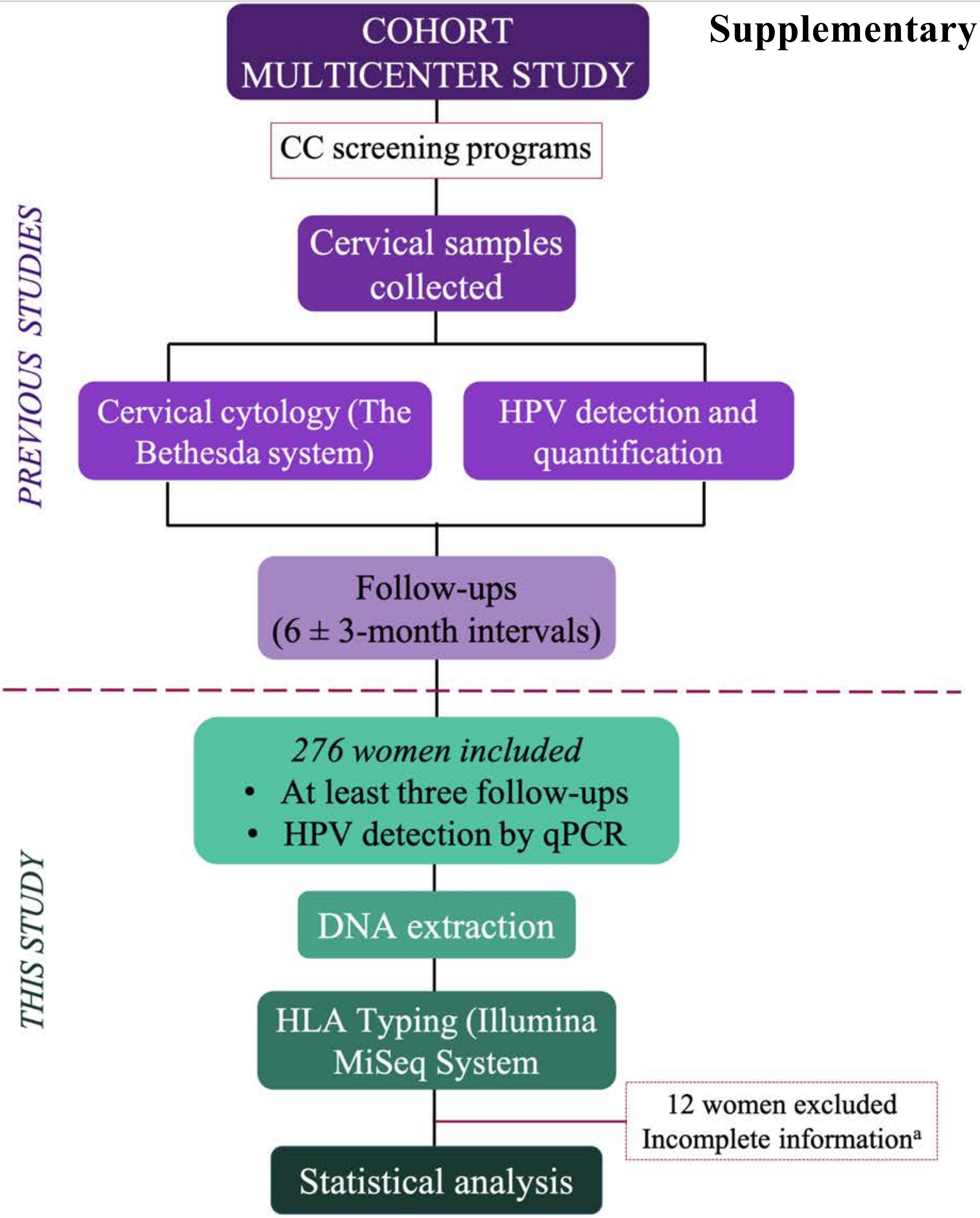

B

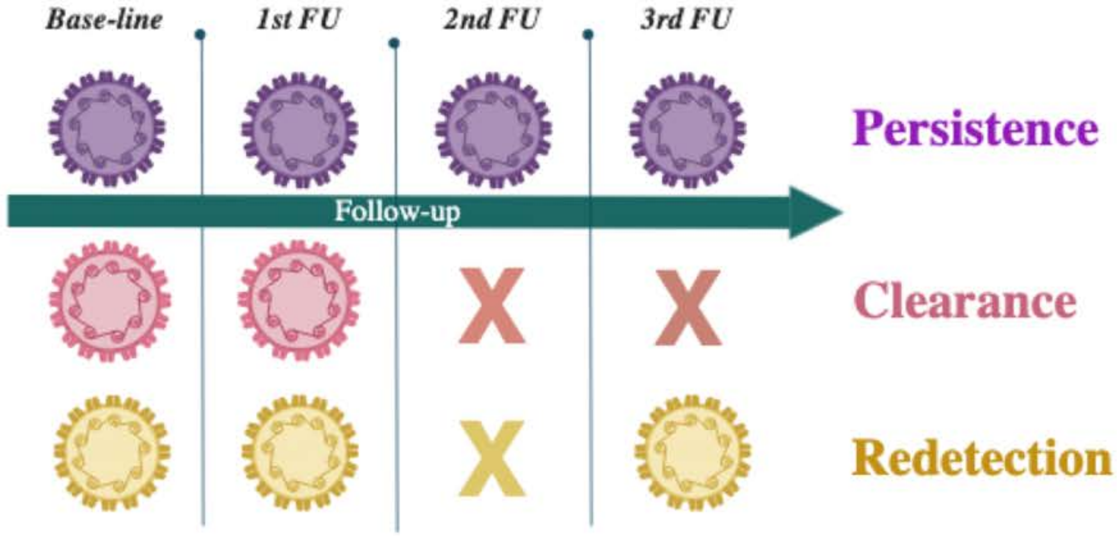

Supplementary Table S1. HLA-*DRB1* and -*DQB1* allele frequency distribution in the study population

Allele	# observed	%	Allele	# observed	%
**DRB1**			**DRB1**		
**01:01:01G**	19	3.47	**11:14:01**	1	0.18
**01:02:01**	15	2.74	**12:01:01G**	5	0.91
**01:02:01G**	5	0.91	**13:01:01G**	29	5.29
**01:03**	1	0.18	**13:02:01G**	23	4.20
**01:03:01**	7	1.28	**13:03:01**	2	0.36
**03:01:01G**	30	5.47	**13:03:01G**	3	0.55
**03:02:01**	2	0.36	**13:04**	2	0.36
**03:02:02**	4	0.73	**13:05:01**	4	0.73
**04:01:01G**	1	0.18	**14:01:01G**	8	1.46
**04:02:01**	9	1.64	**14:02:01**	13	2.37
**04:03:01**	1	0.18	**14:02:01G**	8	1.46
**04:03:01G**	8	1.46	**14:04:01**	1	0.18
**04:04:01**	20	3.65	**14:06:01**	1	0.18
**04:05:01**	9	1.64	**15:01:01G**	38	6.93
**04:05:04**	10	1.82	**15:02:01G**	5	0.91
**04:06:01G**	1	0.18	**15:03:01G**	3	0.55
**04:07:01G**	72	13.14	**16:01:01**	6	1.09
**04:08:01**	2	0.36	**16:02:01G**	29	5.29
**04:10:01G**	2	0.36			
**04:11:01**	10	1.82			
**07:01:01G**	43	7.85	**DQB1**		
**07:11**	2	0.36	**02:01:01G**	74	13.50
**08:01:01G**	5	0.91	**03:01:01G**	110	20.07
**08:02:01**	21	3.83	**03:02:01G**	124	22.63
**08:02:01G**	7	1.28	**03:02:03**	1	0.18
**08:03:02**	1	0.18	**03:03:02G**	8	1.46
**08:04:01**	6	1.09	**04:02:01G**	56	10.22
**08:06**	1	0.18	**05:01:01G**	62	11.31
**08:07**	4	0.73	**05:02:01G**	10	1.82
**09:01:02G**	5	0.91	**05:03:01G**	9	1.64
**10:01:01G**	10	1.82	**06:01:01G**	5	0.91
**11:01:01G**	12	2.19	**06:02:01G**	37	6.75
**11:01:02**	2	0.36	**06:03:01G**	30	5.47
**11:02:01**	6	1.09	**06:04:01G**	8	1.46
**11:04:01**	1	0.18	**06:09:01G**	14	2.55
**11:04:01G**	13	2.37	**Total**	**548**	**100**

HLA: human leukocyte antigen, *DRB1: DR beta 1, DQB1: DQ beta 1*

Supplementary Table S2. HLA *DRB1-DQB1 loci* haplotype frequency distribution in the study population

Haplotype		# observed	%
DRB1	*DQB1*		
**01:01:01G**	**03:01:01G**	6	1.09
**01:01:01G**	**03:02:01G**	6	1.09
**01:01:01G**	**03:03:02G**	2	0.36
**01:01:01G**	**04:02:01G**	2	0.36
**01:01:01G**	**05:01:01G**	6	1.09
**01:02:01G**	**02:01:01G**	4	0.73
**01:02:01G**	**03:01:01G**	4	0.73
**01:02:01G**	**03:02:01G**	6	1.09
**01:02:01G**	**03:03:02G**	2	0.36
**01:02:01G**	**04:02:01G**	4	0.73
**01:02:01G**	**05:01:01G**	6	1.09
**01:03:01**	**02:01:01G**	2	0.36
**01:03:01**	**03:01:01G**	4	0.73
**01:03:01**	**03:02:01G**	2	0.36
**01:03:01**	**04:02:01G**	2	0.36
**01:03:01**	**05:01:01G**	4	0.73
**01:03:01**	**05:02:01G**	2	0.36
**03:01:01G**	**02:01:01G**	6	1.09
**03:01:01G**	**05:01:01G**	2	0.36
**03:02:01**	**02:01:01G**	2	0.36
**03:02:01**	**04:02:01G**	2	0.36
**03:02:02**	**02:01:01G**	2	0.36
**03:02:02**	**03:01:01G**	6	1.09
**04:01:01G**	**03:02:01G**	2	0.36
**04:02:01**	**02:01:01G**	2	0.36
**04:02:01**	**03:01:01G**	6	1.09
**04:02:01**	**03:02:01G**	6	1.09
**04:02:01**	**05:01:01G**	2	0.36
**04:03:01G**	**02:01:01G**	4	0.73
**04:03:01G**	**03:01:01G**	2	0.36
**04:03:01G**	**03:02:01G**	6	1.09
**04:04:01**	**02:01:01G**	2	0.36
**04:04:01**	**03:01:01G**	6	1.09
**04:04:01**	**03:02:01G**	6	1.09
**04:04:01**	**05:01:01G**	2	0.36
**04:05:01**	**02:01:01G**	4	0.73
**04:05:01**	**03:01:01G**	4	0.73
**08:04:01**	**04:02:01G**	2	0.36
**08:04:01**	**05:01:01G**	2	0.36
**08:06**	**06:02:01G**	2	0.36
**08:07**	**04:02:01G**	6	1.09
**09:01:02G**	**03:01:01G**	4	0.73
**09:01:02G**	**03:03:02G**	6	1.09
**10:01:01G**	**05:01:01G**	6	1.09
**11:01:01G**	**03:01:01G**	6	1.09
**11:01:01G**	**03:02:01G**	6	1.09
**11:01:02**	**05:01:01G**	4	0.73
**11:02:01**	**03:01:01G**	6	1.09
**11:02:01**	**03:02:01G**	2	0.36
**11:02:01**	**04:02:01G**	2	0.36
**11:02:01**	**05:01:01G**	2	0.36
**11:04:01G**	**02:01:01G**	2	0.36
**11:04:01G**	**03:01:01G**	6	1.09
**11:04:01G**	**03:02:01G**	4	0.73
**11:04:01G**	**04:02:01G**	2	0.36
**11:04:01G**	**05:01:01G**	4	0.73
**11:04:01G**	**06:03:01G**	2	0.36
**11:14:01**	**04:02:01G**	2	0.36
**12:01:01G**	**03:02:01G**	6	1.09
**12:01:01G**	**04:02:01G**	2	0.36
**13:01:01G**	**03:01:01G**	6	1.09
**13:01:01G**	**05:02:01G**	2	0.36
**13:01:01G**	**05:03:01G**	4	0.73
**13:01:01G**	**06:02:01G**	2	0.36
**13:01:01G**	**06:03:01G**	6	1.09
**13:02:01G**	**03:01:01G**	6	1.09
**13:02:01G**	**05:01:01G**	2	0.36
**13:02:01G**	**06:02:01G**	2	0.36
**13:02:01G**	**06:04:01G**	6	1.09
**13:02:01G**	**06:09:01G**	6	1.09
**13:03:01G**	**03:01:01G**	4	0.73
**13:03:01G**	**03:02:01G**	2	0.36
**13:03:01G**	**04:02:01G**	2	0.36
**13:03:01G**	**05:01:01G**	2	0.36

**04:05:01**	**03:02:01G**	6	1.09
**04:05:04**	**02:01:01G**	6	1.09
**04:05:04**	**03:01:01G**	2	0.36
**04:05:04**	**03:02:01G**	4	0.73
**04:05:04**	**04:02:01G**	6	1.09
**04:05:04**	**05:01:01G**	2	0.36
**04:06:01G**	**04:02:01G**	2	0.36
**04:07:01G**	**02:01:01G**	6	1.09
**04:07:01G**	**03:01:01G**	6	1.09
**04:07:01G**	**03:02:01G**	6	1.09
**04:07:01G**	**03:02:03**	2	0.36
**04:07:01G**	**04:02:01G**	4	0.73
**04:07:01G**	**05:01:01G**	6	1.09
**04:08:01**	**02:01:01G**	2	0.36
**04:08:01**	**03:01:01G**	2	0.36
**04:10:01G**	**03:01:01G**	2	0.36
**04:10:01G**	**04:02:01G**	2	0.36
**04:11:01**	**02:01:01G**	2	0.36
**04:11:01**	**03:02:01G**	6	1.09
**04:11:01**	**04:02:01G**	2	0.36
**07:01:01G**	**02:01:01G**	6	1.09
**07:01:01G**	**03:01:01G**	4	0.73
**07:01:01G**	**03:02:01G**	6	1.09
**07:01:01G**	**03:03:02G**	4	0.73
**07:01:01G**	**04:02:01G**	6	1.09
**07:01:01G**	**05:01:01G**	6	1.09
**07:11**	**03:02:01G**	2	0.36
**07:11**	**04:02:01G**	2	0.36
**08:01:01G**	**03:01:01G**	4	0.73
**08:01:01G**	**04:02:01G**	6	1.09
**08:02:01G**	**03:01:01G**	6	1.09
**08:02:01G**	**04:02:01G**	6	1.09
**08:02:01G**	**05:01:01G**	6	1.09
**08:03:02**	**04:02:01G**	2	0.36
**08:04:01**	**03:01:01G**	2	0.36
**08:04:01**	**03:02:01G**	6	1.09
**13:04**	**03:01:01G**	2	0.36
**13:04**	**04:02:01G**	2	0.36
**13:05:01**	**03:01:01G**	4	0.73
**13:05:01**	**03:02:01G**	4	0.73
**14:01:01G**	**05:03:01G**	6	1.09
**14:01:01G**	**06:03:01G**	4	0.73
**14:02:01G**	**03:01:01G**	6	1.09
**14:02:01G**	**03:02:01G**	6	1.09
**14:02:01G**	**04:02:01G**	4	0.73
**14:02:01G**	**05:01:01G**	2	0.36
**14:02:01G**	**06:04:01G**	2	0.36
**14:02:01G**	**06:09:01G**	2	0.36
**14:04:01**	**03:01:01G**	2	0.36
**14:06:01**	**06:03:01G**	2	0.36
**15:01:01G**	**05:01:01G**	6	1.09
**15:01:01G**	**05:02:01G**	4	0.73
**15:01:01G**	**06:02:01G**	6	1.09
**15:01:01G**	**06:03:01G**	2	0.36
**15:01:01G**	**06:04:01G**	2	0.36
**15:02:01G**	**06:01:01G**	6	1.09
**15:03:01G**	**06:02:01G**	6	1.09
**16:01:01**	**03:01:01G**	2	0.36
**16:01:01**	**05:02:01G**	6	1.09
**16:01:01**	**06:03:01G**	2	0.36
**16:02:01G**	**03:01:01G**	6	1.09
**16:02:01G**	**03:02:01G**	6	1.09
**16:02:01G**	**03:03:02G**	2	0.36
**16:02:01G**	**04:02:01G**	2	0.36
**16:02:01G**	**05:01:01G**	2	0.36
**16:02:01G**	**05:02:01G**	4	0.73
**16:02:01G**	**05:03:01G**	2	0.36
**16:02:01G**	**06:03:01G**	4	0.73
**16:02:01G**	**06:04:01G**	2	0.36
**16:02:01G**	**06:09:01G**	2	0.36
**Total**		**548**	**100**

HLA: human leukocyte antigen, *DRB1: DR beta 1, DQB1: DQ beta 1*

Supplementary Table S3. Distribution of sociodemographic variables, risk factors and cervical cytology findings, showing infection clearance, persistence or non-detection-redetection events for HPV-16, -18 and -31

Characteristic	HPV-16 (n=328)							HPV-18 (n=300)							HPV-31 (n=316)						
	Clearance		Persistence		ND-RD		P[a]	Clearance		Persistence		ND-RD		P[a]	Clearance		Persistence		ND-RD		P[a]
	n	%	n	%	n	%		n	%	n	%	n	%		n	%	n	%	n	%	
**Age, years**																					
**≤30**	19	43.18	8	18.18	17	38.64	0.343	13	28.89	13	28.89	19	42.22	0.216	10	21.28	22	46.81	15	31.91	0.262
**31-45**	48	32.43	19	12.84	81	54.73		30	21.90	46	33.58	61	44.53		33	23.74	41	29.50	65	46.76	
**>45**	52	38.24	22	16.18	62	45.59		23	19.49	53	44.92	42	35.59		31	23.85	41	31.54	58	44.62	
**Origin**																					
**Chaparral**	3	42.86	1	14.29	3	42.86	0.058	2	25.00	2	25.00	4	50.00	0.771	1	14.29	3	42.86	0	42.86	0.292
**Bogotá**	23	25.00	19	20.65	50	54.35		18	22.22	34	41.98	29	35.80		29	28.16	26	25.24	0	46.60	
**Girardot**	93	40.61	29	12.66	107	46.72		46	21.80	76	36.02	89	42.18		44	21.36	75	36.41	87	42.23	
**Ethnicity**																					
**Afro-descendant**	3	42.86	0	0.00	4	57.14	0.884	1	25.00	2	50.00	1	25.00	0.933	2	33.33	1	16.67	3	50.00	0.722
**Mestizo**	116	36.25	49	15.31	155	48.44		65	22.03	110	37.29	120	40.68		71	23.05	103	33.44	134	43.51	
**Indigenous**	0	0.00	0	0.00	1	100		0	0.00	0	0.00	1	100		1	50.00	0	0.00	1	50.00	
**Smoker**																					
**No**	90	36.44	35	14.17	122	49.39	0.787	46	20.09	86	37.55	97	42.36	0.316	55	23.11	81	34.03	102	42.86	0.758
**Yes**	29	35.80	14	17.28	38	46.91		20	28.17	26	36.62	25	35.21		19	24.36	23	29.49	36	46.15	
**Age on first intercourse, years**																					
**≤18**	70	36.27	33	17.10	90	46.63	0.385	36	20.45	68	38.64	72	40.91	0.718	41	22.53	66	36.26	75	41.21	0.332
**>18**	49	36.30	16	11.85	70	51.85		30	24.19	44	35.48	50	40.32		33	24.63	38	28.36	63	47.01	
**Lifetime amount of sexual partners**																					
**1**	50	35.71	25	17.86	65	46.43	0.363	29	21.64	52	38.81	53	39.55	0.907	33	24.26	50	36.76	53	38.97	0.389
**>1**	67	37.22	22	12.22	91	50.56		35	22.29	57	36.31	65	41.40		39	22.67	53	30.81	80	46.51	
**Contraceptive method**																					
**None**	43	36.75	15	12.82	59	50.43	0.568	21	18.75	49	43.75	42	37.50	0.363	27	22.69	36	30.25	56	47.06	0.605

Other	54	34.84	23	14.84	78	50.32		33	24.26	46	33.82	57	41.91		34	24.29	51	36.43	55	39.29	
Hormonal	14	48.28	5	17.24	10	34.48		8	24.24	9	27.27	16	48.48		6	16.67	12	33.33	18	50.00	
**Births**																					
None	2	25.00	0	0.00	6	75.00	0.768	2	33.33	3	50.00	1	16.67	0.703	1	9.09	3	27.27	7	63.64	0.604
1-2	55	36.91	21	14.09	73	48.99		34	23.13	51	34.69	62	42.18		41	26.28	51	32.69	64	41.03	
>2	57	36.08	24	15.19	77	48.73		28	20.59	52	38.24	56	41.18		30	21.74	46	33.33	62	44.93	
**Abortions**																					
No	33	27.73	25	21.01	61	51.26	0.139	30	29.41	37	36.27	35	34.31	**0.037**	33	27.50	38	31.67	49	40.83	0.420
Yes	45	38.14	16	13.56	57	48.31		20	17.54	37	32.46	57	50.00		23	20.18	39	34.21	52	45.61	
**STD**																					
No	92	36.08	41	16.08	122	47.84	0.292	45	19.40	89	38.36	98	42.24	0.113	57	23.36	80	32.79	107	43.85	0.946
Yes	24	39.34	5	8.20	32	52.46		17	32.08	19	35.85	17	32.08		15	25.00	20	33.33	25	41.67	
**Cytological findings**																					
Negative	108	35.88	44	14.62	149	49.50	0.275	64	23.19	101	36.59	111	40.22	**0.047**	64	22.70	93	32.98	125	44.33	0.652
ASC-US	3	37.50	1	12.50	4	50.00		0	0.00	7	70.00	3	30.00		2	25.00	3	37.50	3	37.50	
LSIL	7	70.00	1	10.00	2	20.00		2	40.00	3	60.00	0	0.00		0	0.00	1	100	0	0.00	
HSIL	0	0.00	0	0.00	0	0.00		0	0.00	0	0.00	1	100.00		5	41.67	3	25.00	4	33.33	
**Viral load[b]**																					
Low	43	31.62	16	11.76	77	56.62	**0.000**	7	23.33	12	40.00	11	36.67	0.985	6	19.35	10	32.26	15	48.39	0.667
Medium	69	47.26	23	15.75	54	36.99		48	22.12	81	37.33	88	40.55		50	26.32	60	31.58	80	42.11	
High	7	15.22	10	21.74	29	63.04		11	20.75	19	35.85	23	43.40		18	18.95	34	35.79	43	45.26	

95%CI: 95% confidence interval, ND-RD: no-detection/redetection, STD: sexually transmitted disease, ASC-US: atypical squamous cells of undetermined significance, LSIL: low-grade squamous intraepithelial lesion, HSIL: high-grade squamous intraepithelial lesion, HPV: human papillomavirus

[a] Chi squared or exact Fisher tests were used

[b] Viral load, low: ≤9.99E+5, medium: 1.00E+6 to 9.99E+9, high: ≥1.00E+10

Supplementary Table S4. Distribution of sociodemographic variables, risk factors, and cervical cytology findings, showing infection clearance, persistence or non-detection-redetection events for HPV-33, -45 and -58

Characteristic	HPV-33 (n=163)							HPV-45 (n=285)							HPV-58 (n=264)						
	Clearance		Persistence		ND-RD		P[a]	Clearance		Persistence		ND-RD		P[a]	Clearance		Persistence		ND-RD		P[a]
	n	%	n	%	n	%		n	%	n	%	n	%		n	%	n	%	n	%	
**Age, years**																					
**≤30**	7	29.17	2	8.33	15	62.50	0.895	18	43.90	6	14.63	17	41.46	0.618	18	40.91	3	6.82	23	52.27	0.490
**31-45**	16	24.62	4	6.15	45	69.23		39	31.71	21	17.07	63	51.22		44	37.29	20	16.95	54	45.76	
**>45**	23	31.08	5	6.76	46	62.16		39	32.23	24	19.83	58	47.93		38	37.25	19	18.63	45	44.12	
**Origin**																					
**Chaparral**	0	0.00	1	20.00	4	80.00	**0.001**	1	11.11	2	22.22	6	66.67	**0.044**	3	42.86	0	0.00	4	57.14	**0.000**
**Bogotá**	9	14.29	2	3.17	52	82.54		17	22.37	16	21.05	43	56.58		44	57.14	11	14.29	22	28.57	
**Girardot**	37	38.95	8	8.42	50	52.63		78	39.00	33	16.50	89	44.50		53	29.44	31	17.22	96	53.33	
**Ethnicity**																					
**Afro-descendant**	2	50.00	0	0.00	2	50.00	0.667	4	66.67	0	0.00	2	33.33	0.163	2	33.33	1	16.67	3	50.00	0.626
**Mestizo**	43	27.39	11	7.01	103	65.61		91	32.85	50	18.05	136	49.10		96	37.50	41	16.02	119	46.48	
**Indigenous**	1	50.00	0	0.00	1	50.00		1	50.00	1	50.00	0	0.00		2	100.00	0	0.00	0	0.00	
**Smoker**																					
**No**	35	27.56	9	7.09	83	65.35	0.951	75	34.56	42	19.35	100	46.08	0.314	73	36.50	30	15.00	97	48.50	0.411
**Yes**	11	30.56	2	5.56	23	63.89		21	30.88	9	13.24	38	55.88		27	42.19	12	18.75	25	39.06	
**Age on first intercourse, years**																					
**≤18**	21	23.08	5	5.49	65	71.43	0.157	60	36.14	29	17.47	77	46.39	0.578	64	41.29	24	15.48	67	43.23	0.383
**>18**	25	34.72	6	8.33	41	56.94		36	30.25	22	18.49	61	51.26		36	33.03	18	16.51	55	50.46	
**Lifetime amount of sexual partners**																					
**1**	20	28.57	6	8.57	44	62.86	0.734	40	31.25	27	21.09	61	47.66	0.436	43	39.09	16	14.55	51	46.36	0.812
**>1**	25	27.78	5	5.56	60	66.67		53	35.33	23	15.33	74	49.33		55	36.91	26	17.45	68	45.64	
**Contraceptive method**																					
**None**	19	32.76	0	0.00	39	67.24	**0.016**	34	33.01	23	22.33	46	44.66	0.526	37	37.37	15	15.15	47	47.47	0.937

**Other**	15	21.13	8	11.27	48	67.61		40	31.75	22	17.46	64	50.79		45	38.14	16	13.56	57	48.31	
**Hormonal**	10	41.67	2	8.33	12	50.00		14	45.16	5	16.13	12	38.71		11	36.67	6	20.00	13	43.33	
**Births**																					
**None**	1	16.67	0	0.00	5	83.33	0.801	1	16.67	2	33.33	3	50.00	0.490	4	36.36	1	9.09	6	54.55	0.946
**1-2**	19	24.05	5	6.33	55	69.62		47	35.61	19	14.39	66	50.00		49	37.98	22	17.05	58	44.96	
**>2**	22	30.56	6	8.33	44	61.11		43	31.85	28	20.74	64	47.41		47	41.59	18	15.93	48	42.48	
**Abortions**																					
**No**	21	29.58	5	7.04	45	63.38	0.793	33	30.28	24	22.02	52	47.71	0.233	39	39.80	19	19.39	40	40.82	0.507
**Yes**	14	24.56	5	8.77	38	66.67		34	34.00	13	13.00	53	53.00		35	34.31	17	16.67	50	49.02	
**STD**																					
**No**	38	31.67	9	7.50	73	60.83	0.090	66	30.00	43	19.55	111	50.45	**0.046**	73	35.27	33	15.94	101	48.79	0.198
**Yes**	5	14.29	2	5.71	28	80.00		24	48.00	6	12.00	20	40.00		23	48.94	7	14.89	17	36.17	
**Cytological findings**																					
**Negative**	43	28.86	8	5.37	98	65.77	0.080	91	34.47	45	17.05	128	48.48	0.261	88	36.97	37	15.55	113	47.48	0.070
**ASC-US**	1	100	0	0.00	0	0.00		1	25.00	0	0.00	3	75.00		5	71.43	2	28.57	0	0.00	
**LSIL**	0	0.00	0	0.00	0	0.00		0	0.00	0	0.00	0	0.00		0	0.00	0	0.00	1	100.00	
**HSIL**	1	14.29	2	28.57	4	57.14		1	16.67	3	50.00	2	33.33		4	36.36	3	27.27	4	36.36	
**Viral loa [b]**																					
**Low**	21	56.76	3	8.11	13	35.14	**0.000**	38	55.88	12	17.65	18	26.47	**0.000**	47	36.43	23	17.83	59	45.74	0.450
Medium	16	18.39	4	4.60	67	77.01		42	26.25	33	20.63	85	53.13		44	41.90	12	11.43	49	46.67	
**High**	9	23.08	4	10.26	26	66.67		16	28.07	6	10.53	35	61.40		9	30.00	7	23.33	14	46.67	

95%CI: 95% confidence interval, ND-RD: no-detection/redetection, STD: sexually transmitted disease, ASC-US: atypical squamous cells of undetermined significance, LSIL: low-grade squamous intraepithelial lesion, HSIL: high-grade squamous intraepithelial lesion, HPV: human papillomavirus

[a] Chi squared or exact Fisher tests were used

[b] Viral load, low: ≤9.99E+5, medium: 1.00E+6 to 9.99E+9, high: ≥1.00E+10

Supplementary Table S5. Multivariate models evaluating the association between *DRB1* and *DQB1* alleles and infection clearance/persistence for HPV-16, -18 and -31

Allele	HPV-16			HPV-18			HPV-31		
	Coeff.	95%CI		Coeff.	95%CI		Coeff.	95%CI	
DRB1									
**01:01:01G**	0.31	0.91	1.05	0.32	0.08	1.33	-0.44[a]	-0.89	0.02
**01:02:01G**	1.08	0.58	2.03	**2.50**	**1.12**	**5.60**	0.32	0.06	1.68
**01:03:01**	3.39E-20	.	.	1.35	0.26	6.99	**-0.75[a]**	**-1.31**	**-0.19**
**03:01:01G**	0.99	0.43	2.25	0.95	0.37	2.40	0.09[a]	-0.51	0.68
**03:02:01**	**7.16E-18**	**8.15E-19**	**6.30E-17**	1.64	0.91	2.98	**-1.55[a]**	**-2.01**	**-1.09**
**03:02:02**	1.92	0.75	4.92	3.74	0.85	1.64	1.89	0.69	5.16
**04:01:01G**	-	.	.	1.00	.	.	-	.	.
**04:02:01**	0.34[a]	-0.52	1.20	0.41	0.05	3.46	**8.00E-16**	**2.99E-16**	**2.14E-15**
**04:03:01G**	1.25	0.32	4.87	1.35	0.09	2.08	-0.09[a]	-1.06	0.88
**04:04:01**	1.91	0.96	3.82	0.23	0.03	2.00	0.97	0.29	3.21
**04:05:01**	**3.16**	**1.59**	**6.28**	1.58	0.42	5.96	0.21	0.03	1.61
**04:05:04**	0.94	0.18	4.73	2.18	0.89	5.38	0.44[a]	-0.64	1.53
**04:07:01G**	0.88	0.53	1.44	1.01	0.52	1.95	1.68	0.95	2.96
**04:08:01**	**3.64E-16**	**4.11E-17**	**3.23E-15**	**4.22**	**1.40**	**1.27**	0.21[a]	-1.11	1.53
**04:10:01G**	-	.	.	1.00	.	.	-	.	.
**04:11:01**	1.25	0.68	2.29	2.67	0.98	7.25	**4.43E-15**	**1.37E-15**	**1.43E-14**
**07:01:01G**	1.12	0.58	2.17	1.85	0.91	3.78	1.17	0.59	2.32
**07:11**	**3.47E-17**	**7.58E-18**	**1.59E-16**	**15.33**	**5.17**	**45.46**	1.92	0.23	16.33
**08:01:01G**	0.41	0.04	4.06	1.00	0.18	5.51	**1.15E-14**	**3.17E-15**	**4.15E-14**
**08:02:01G**	-0.01[a]	-0.58	0.56	1.51	0.53	4.34	0.13[a]	-0.55	0.80
**08:03:02**	-	.	.	1.00	.	.	-	.	.
**08:04:01**	1.50	0.24	9.16	0.74	0.15	3.60	**1.33E-15**	**3.53E-16**	**4.99E-15**
**08:06**	-	.	.	1.00	.	.	-	.	.
**08:07**	5.25	0.17	162.75	**-1.47[a]**	**-2.10**	**-0.84**	**2.96**	**1.47**	**5.96**
**09:01:02G**	1.40	0.58	3.39	1.60	0.19	13.51	0.73	0.15	3.45
**10:01:01G**	0.62	0.24	1.61	1.78	0.63	5.04	2.93	0.81	10.64
**11:01:01G**	0.55	0.17	1.76	0.86	0.25	3.00	-0.53[a]	-1.30	0.25
**11:01:02**	-	.	.	1.00	.	.	-	.	.
**11:02:01**	1.22	0.55	2.70	3.51	0.56	21.87	**5.15E-15**	**1.31E-15**	**2.02E-14**
**11:04:01G**	0.50	0.23	1.11	0.68	0.15	3.07	-0.01[a]	-0.69	0.68
**11:14:01**	1.25	0.42	3.76	**3.43**	**1.21**	**9.74**	**1.58E-16**	**1.89E-17**	**1.33E-15**
**12:01:01G**	**4.80**	**2.67**	**8.64**	**2.79E-15**	**2.74E-16**	**2.84E-14**	**8.24E-17**	**1.68E-17**	**4.03E-16**
**13:01:01G**	1.31	0.61	2.79	0.27	0.06	1.35	1.39	0.66	2.93
**13:02:01G**	0.51	0.14	1.91	0.23	0.04	1.55	0.94	0.26	3.38
**13:03:01G**	1.41	0.53	3.70	**2.29E-16**	**4.88E-17**	**1.08E-15**	0.04[a]	-1.42	1.50
**13:04**	0.44	0.04	5.32	**5.04E-19**	**7.32E-20**	**3.48E-18**	**1.19E-14**	**2.24E-15**	**6.27E-14**
**13:05:01**	**3.21**	**1.68**	**6.12**	**6.84E-16**	**1.90E-16**	**2.46E-15**	**4.20[a]**	**3.08**	**5.31**
**14:01:01G**	2.24	0.91	5.54	1.70	0.74	3.92	0.66	0.17	2.59

**14:02:01G**	1.55	0.78	3.11	0.64	0.15	2.75	0.05[a]	-0.57	0.68
**14:04:01**	-	.	.	1.00	.	.	-	.	.
**14:06:01**	-	.	.	1.00	.	.	-	.	.
**15:01:01G**	1.04	0.48	2.24	1.58	0.75	3.32	-0.09[a]	-0.66	0.48
**15:02:01G**	0.19[a]	-1.52	1.89	**3.74E-16**	**7.98E-17**	**1.75E-15**	0.83	0.08	8.36
**15:03:01G**	**20.24**	**8.61**	**47.55**	**1.89E-17**	**3.79E-18**	**9.44E-17**	1.24	0.06	27.89
**16:01:01**	0.92	0.17	4.89	0.65	0.10	4.29	0.98	0.17	5.54
**16:02:01G**	0.95	0.47	1.91	0.59	0.20	1.75	0.80	0.35	1.87
DQB1									
**02:01:01G**	0.93	0.53	1.64	1.53	0.79	2.95	0.96	0.50	1.85
**03:01:01G**	1.01	0.62	1.62	0.93	0.50	1.73	0.21[a]	-0.14	0.57
**03:02:01G**	1.15	0.73	1.80	0.91	0.51	1.62	1.03	0.58	1.81
**03:02:03**	**2.25**	**1.23**	**4.11**	**1.62E-15**	**2.00E-16**	**1.30E-14**	**-1.22[a]**	**-1.67**	**-0.78**
**03:03:02G**	1.82	0.73	4.57	2.49	0.45	13.65	0.96	0.34	2.66
**04:02:01G**	0.91	0.41	2.01	**-0.65[a]**	**-1.16**	**-1.16**	0.03[a]	-0.44	0.51
**05:01:01G**	0.57	0.31	1.02	1.20	0.61	2.35	1.62	0.81	3.23
**05:02:01G**	0.48	0.09	2.53	0.67	0.19	2.36	-0.24[a]	-1.11	0.64
**05:03:01G**	2.24	0.91	5.54	1.70	0.74	3.92	0.66	0.17	2.59
**06:01:01G**	0.18[a]	-1.52	1.89	**3.74E-16**	**7.98E-17**	**1.75E-15**	0.83	0.08	8.36
**06:02:01G**	1.44	0.68	3.06	1.00	0.41	2.46	1.06	0.40	2.80
**06:03:01G**	1.31	0.61	2.79	0.27	0.06	1.35	1.39	0.66	2.93
**06:04:01G**	0.82	0.25	2.63	6.01.E-20	.	.	0.56	0.08	4.19
**06:09:01G**	0.30	0.02	3.68	0.37	0.06	2.31	1.19	0.22	6.56

Analysis was adjusted for origin, age, lifetime amount of sexual partners, abortions, coinfection and viral load (low: ≤9.99E+5, medium: 1.00E+6 to 9.99E+9, high: ≥1.00E+10).

95%CI: 95% confidence interval, HPV: human papillomavirus, Coeff: regression coefficient (hazard ratio for Cox proportional hazards regression model and time ratio for log-normal parametric model), *DRB1: DR beta 1, DQB1: DQ beta 1*

Values in bold indicate statistical significance based on the 95%CI, $p<0.05$

[a] The Cox proportional hazards model did not fulfil the the assumption of proportionality; the log-normal parametric model was used in such cases

Supplementary Table S6. Multivariate models evaluating the association between *DRB1* and *DQB1* alleles and infection clearance/persistence for HPV-33, -45 and -58

Allele	HPV-33			HPV-45			HPV-58		
	Coeff.	95%CI		Coeff.	95%CI		Coeff.	95%CI	
DRB1									
**01:01:01G**	1.47	0.46	4.68	1.14	0.45	2.90	1.14	0.61	2.11
**01:02:01G**	0.50	0.08	2.97	0.05[a]	-0.49	0.58	-0.19[a]	-0.62	0.23
**01:03:01**	1.73	0.54	5.49	**2.92**	**1.42**	**5.98**	-0.51[a]	-1.23	0.21
**03:01:01G**	**3.49**	**1.32**	**9.26**	0.79	0.36	1.71	2.15E-04[a]	-0.39	0.40
**03:02:01**	**8.59**	**2.76**	**26.73**	**1.65E-16**	**2.13E-17**	**1.28E-15**	**8.69E-17**	**1.02E-17**	**7.40E-16**
**03:02:02**	**2.60E-10**	**2.52E-11**	**2.68E-09**	0.07[a]	-0.92	1.07	1.42	0.45	4.52
**04:01:01G**	1.00	.	.	1.00	.	.	-	.	.
**04:02:01**	0.38	0.04	3.39	0.96	0.24	3.87	-0.04[a]	-1.12	1.05
**04:03:01G**	1.94E-19	.	.	2.17	0.60	7.86	2.29	0.66	7.97
**04:04:01**	6.15E-20	.	.	0.65	0.15	2.81	0.09[a]	-0.64	0.81
**04:05:01**	0.01[a]	-0.87	0.88	0.83	0.27	2.53	0.43[a]	-0.23	1.09
**04:05:04**	1.06	0.43	2.63	0.79	0.19	3.32	0.40	0.10	1.55
**04:07:01G**	1.07	0.49	2.33	0.83	0.48	1.42	**1.72**	**1.03**	**2.85**
**04:08:01**	0.42	0.15	1.19	0.77	0.39	1.53	2.13	0.44	10.25
**04:10:01G**	**1.11E-17**	**1.11E-18**	**1.11E-16**	**4.02E-14**	**4.79E-15**	**3.37E-13**	5.36E-19	.	.
**04:11:01**	8.78	.	.	**5.48E-16**	**1.56E-16**	**1.92E-15**	**1.71E-18**	**5.97E-19**	**4.92E-18**
**07:01:01G**	0.68	0.21	2.22	0.89	0.42	1.88	0.95	0.51	1.77
**07:11**	1.00	.	.	**5.15E-19**	**6.56E-20**	**4.05E-18**	**3.52[a]**	**2.93**	**4.11**
**08:01:01G**	1.06	0.43	2.63	0.64	0.08	5.14	0.31[a]	-0.18	0.81
**08:02:01G**	0.65	0.12	3.48	0.23	0.05	1.08	0.17[a]	-0.35	0.70
**08:03:02**	1.00	.	.	1.00	.	.	-	.	.
**08:04:01**	**-0.67[a]**	**-0.97**	**-0.36**	-0.58[a]	-1.39	0.23	**3.80**	**1.47**	**9.86**
**08:06**	1.00	.	.	1.00	.	.	-	.	.
**08:07**	**6.79E-17**	**7.26E-18**	**6.35E-16**	**6.78E-15**	**7.83E-16**	**5.87E-14**	**-0.28[a]**	**-0.52**	**-0.05**
**09:01:02G**	1.00	.	.	**-0.70[a]**	**-1.16**	**-0.24**	0.84	0.33	2.17
**10:01:01G**	**3.79**	**1.50**	**9.55**	0.38	0.07	2.19	0.60	0.08	4.62
**11:01:01G**	1.31	0.26	6.59	0.36	0.05	2.73	0.58[a]	-0.44	1.60
**11:01:02**	1.00	.	.	1.00	.	.	-	.	.
**11:02:01**	3.32E-04	.	.	**1.97**	**1.08**	**3.59**	0.02[a]	-0.65	0.69
**11:04:01G**	0.84	0.28	2.55	0.76	0.33	1.76	0.53[a]	-0.10	1.16
**11:14:01**	1.00	.	.	**8.05E-16**	**1.03E-16**	**6.30E-15**	**4.08[a]**	**3.35**	**4.81**
**12:01:01G**	**3.58**	**1.34**	**9.58**	**12.65**	**4.93**	**32.45**	**1.65E-14**	**3.59E-15**	**7.55E-14**
**13:01:01G**	0.75	0.30	1.86	**1.93**	**1.07**	**3.50**	0.08[a]	-0.38	0.53
**13:02:01G**	0.83	0.29	2.36	1.13	0.47	2.72	-0.32[a]	-0.95	0.30
**13:03:01G**	**10.94**	**2.66**	**45.00**	**22.15**	**8.34**	**58.85**	**-0.57[a]**	**-1.13**	**-0.01**
**13:04**	1.00	.	.	**2.67**	**1.22**	**5.81**	**2.75[a]**	**2.11**	**3.39**
**13:05:01**	1.00	.	.	1.25	0.13	12.37	**1.09E-17**	**1.65E-18**	**7.24E-17**
**14:01:01G**	4.73E-20	.	.	0.51[a]	-0.33	1.35	0.03[a]	-0.74	0.80

**14:02:01G**	0.55	0.06	5.39	1.28	0.62	2.68	1.43	0.60	3.39
**14:04:01**	1.00	.	.	1.00	.	.	-	.	.
**14:06:01**	1.00	.	.	1.00	.	.	-	.	.
**15:01:01G**	1.26	0.42	3.80	-0.17[a]	-0.52	0.17	0.07[a]	-0.35	0.48
**15:02:01G**	1.49E-20	.	.	-0.15[a]	-0.97	0.68	-0.24[a]	-0.98	0.50
**15:03:01G**	1.03E-19	.	.	2.04	0.21	20.27	1.68	0.29	9.58
**16:01:01**	0.02	.	.	-0.13[a]	-0.71	0.44	-0.08[a]	-0.80	0.63
**16:02:01G**	1.61	0.58	4.47	1.77	0.89	3.49	1.16	0.60	2.25
DQB1									
**02:01:01G**	1.54	0.60	3.94	0.67	0.38	1.18	0.09[a]	-0.22	0.41
**03:01:01G**	1.09	0.54	2.22	1.34	0.80	2.26	1.15	0.70	1.89
**03:02:01G**	0.88	0.40	1.94	0.71	0.43	1.19	-0.09[a]	-0.36	0.18
**03:02:03**	1.00	.	.	**7.39E-16**	**3.63E-17**	**1.50E-14**	**1.51E-15**	**1.76E-16**	**1.29E-14**
**03:03:02G**	1.30	0.09	1.86	2.39	0.57	9.97	0.99	0.46	2.13
**04:02:01G**	0.67	0.24	1.91	0.32	0.12	0.87	0.29[a]	-0.05	0.63
**05:01:01G**	0.75	0.33	1.72	-0.16[a]	-0.46	0.13	1.14	0.64	2.01
**05:02:01G**	**8.59**	**2.76**	**2.67**	0.18[a]	-0.46	0.82	0.20[a]	-0.51	0.92
**05:03:01G**	4.73E-20	.	.	0.51[a]	-0.33	1.35	0.03[a]	-0.74	0.80
**06:01:01G**	1.49E-20	.	.	-0.15[a]	-0.97	0.68	-0.24[a]	-0.98	0.50
**06:02:01G**	1.65	0.74	3.67	-0.24[a]	-0.55	0.08	-0.06[a]	-0.44	0.31
**06:03:01G**	0.90	0.42	1.91	**1.82**	**1.08**	**3.07**	0.10[a]	-0.34	0.54
**06:04:01G**	2.26E-20	.	.	0.70	0.20	2.42	0.78	0.14	4.44
**06:09:01G**	1.36	0.51	3.64	1.33	0.40	4.37	-0.53[a]	-1.25	0.19

Analysis was adjusted for origin, age, lifetime amount of sexual partners, abortions, coinfection and viral load (low: ≤9.99E+5, medium: 1.00E+6 to 9.99E+9, high: ≥1.00E+10).
95%CI: 95% confidence interval, HPV: human papillomavirus, Coeff: regression coefficient (hazard ratio for Cox proportional hazards regression model and time ratio for log-normal parametric model), *DRB1: DR beta 1, DQB1: DQ beta 1*
Values in bold indicate statistical significance based on the 95%CI, $p<0.05$
[a] The Cox proportional hazards model did not fulfil thethe assumption of proportionality; the log-normal parametric model was used in such cases

Supplementary Table S7. Multivariate models evaluating the association between *DRB1* and *DQB1* haplotypes and infection clearance/persistence for HPV-16, -18 and -31

**Haplotype**		**HPV-16**			**HPV-18**			**HPV-31**		
DRB1	***DQB1***	**Coeff.**	**95%CI**		**Coeff.**	**95%CI**		**Coeff.**	**95%ICI**	
**01:01:01G**	**03:01:01G**	0.51	0.12	2.07	**2.00E-16**	**3.72E-17**	**1.08E-15**	-0.57[a]	-1.02	-0.11
**01:01:01G**	**03:02:01G**	**4.30E-16**	**1.01E-16**	**1.83E-16**	2.12	0.36	12.43	1.69	0.49	5.79
**01:01:01G**	**03:03:02G**	1.00	.	.	1.00	.	.	1.00	.	.
**01:01:01G**	**04:02:01G**	1.00	.	.	**2.32E-15**	**2.64E-16**	**2.05E-14**	**7.52E-14**	**9.37E-15**	**6.04E-13**
**01:01:01G**	**05:01:01G**	0.32	0.09	1.11	0.31	0.07	1.32	**-0.51[a]**	**-0.95**	**-0.71**
**01:02:01G**	**02:01:01G**	**2.43E-16**	**4.56E-16**	**1.29E-16**	**1.63E-15**	**1.58E-16**	**1.67E-14**	**4.05E-16**	**8.02E-17**	**2.05E-15**
**01:02:01G**	**03:01:01G**	1.11	0.35	3.44	**4.57**	**1.745**	**12.00**	9.30E-20	.	.
**01:02:01G**	**03:02:01G**	1.93	0.65	5.67	**4.96**	**1.62**	**15.14**	-0.40[a]	-1.43	0.63
**01:02:01G**	**03:03:02G**	**3.11**	**1.72**	**5.63**	**2.20E-15**	**2.51E-16**	**1.93E-14**	**1.64E-15**	**2.10E-16**	**1.28E-14**
**01:02:01G**	**04:02:01G**	1.00	.	.	1.00	.	.	1.00	.	.
**01:02:01G**	**05:01:01G**	1.08	0.57	2.03	**2.50**	**.12**	**5.59**	0.32	0.06	1.68
**01:03:01**	**02:01:01G**	1.00	.	.	**2.72E-16**	**2.84E-17**	**2.61E-15**	**6.31**	**2.84**	**1.40**
**01:03:01**	**03:01:01G**	**1.21E-17**	**1.50E-18**	**9.80E-17**	1.00	.	.	-0.67[a]	-1.85	0.49
**01:03:01**	**03:02:01G**	**6.63E-17**	**5.97E-18**	**7.36E-16**	1.89E-19	.	.	1.00	.	.
**01:03:01**	**04:02:01G**	1.00	.	.	**4.24**	**1.53**	**11.69**	**3.48E-14**	**4.04E-15**	**3.00E-13**
**01:03:01**	**05:01:01G**	**7.48E-16**	**9.57E-17**	**5.84E-15**	1.35	0.26	6.99	**-0.75[a]**	**-1.31**	**-0.19**
**01:03:01**	**05:02:01G**	1.00	.	.	1.00	.	.	1.00	.	.
**03:01:01G**	**02:01:01G**	0.98	0.43	2.25	0.94	0.37	2.40	0.08[a]	-0.50	0.68
**03:01:01G**	**05:01:01G**	1.88E-19	.	.	1.00	.	.	**1.06E-14**	**1.18E-15**	**9.49E-14**
**03:02:01**	**02:01:01G**	1.00	.	.	1.00	.	.	1.00	.	.
**03:02:01**	**04:02:01G**	**7.86E-15**	**8.94E-16**	**6.90E-14**	1.64	0.90	2.98	**-1.54[a]**	**-2.01**	**-1.08**
**03:02:02**	**02:01:01G**	1.00	.	.	**38.11**	**9.46**	**153.43**	**3.88**	**1.56**	**9.65**
**03:02:02**	**03:01:01G**	1.91	0.74	4.91	3.73	0.84	16.42	1.88	0.68	5.16
**04:01:01G**	**03:02:01G**	1.00	.	.	1.00	.	.	1.00	.	.
**04:02:01**	**02:01:01G**	1.00	.	.	1.11E-19	.	.	**2.24E-14**	**2.72E-15**	**1.84E-13**
**04:02:01**	**03:01:01G**	1.05	0.28	3.93	**1.11E-16**	**1.16E-17**	**1.05E-15**	**2.65E-16**	**5.63E-17**	**1.25E-15**
**04:02:01**	**03:02:01G**	0.34[a]	-0.33	1.01	0.40	0.04	3.45	**8.00E-16**	**2.99E-16**	**2.14E-15**
**04:02:01**	**05:01:01G**	1.00	.	.	1.00	.	.	1.00	.	.
**04:03:01G**	**02:01:01G**	0.60	0.32	1.13	**9.79**	**2.10**	**45.67**	**4.53**	**2.13**	**9.62**
**04:03:01G**	**03:01:01G**	1.00	.	.	**1.20E-14**	**1.31E-15**	**1.11E-13**	**1.23E-15**	**1.22E-16**	**1.23E-14**
**04:03:01G**	**03:02:01G**	1.48	0.33	6.65	**5.43E-15**	**8.14E-16**	**3.62E-14**	0.37[a]	-0.83	1.58
**04:04:01**	**02:01:01G**	1.81	0.92	3.55	**4.76E-16**	**5.87E-17**	**3.86E-15**	**1.79E-16**	**1.99E-17**	**1.61E-15**
**04:04:01**	**03:01:01G**	**4.24**	**2.39**	**7.55**	**5.37E-18**	**1.18E-18**	**2.45E-17**	-0.19[a]	-0.98	0.59
**04:04:01**	**03:02:01G**	1.91	0.95	3.81	0.23	0.02	1.99	1.10	0.32	3.75
**04:04:01**	**05:01:01G**	**2.01E-18**	**2.26E-19**	**1.79E-17**	1.00	.	.	**1.73E-16**	**2.10E-17**	**1.42E-15**
**04:05:01**	**02:01:01G**	0.94	0.47	1.85	**4.59**	**1.39**	**15.15**	0.20[a]	-1.00	1.42
**04:05:01**	**03:01:01G**	**4.92**	**2.49**	**9.71**	3.20	0.34	30.16	**2.90E-15**	**5.71E-16**	**1.48E-14**
**04:05:01**	**03:02:01G**	**3.16**	**1.59**	**6.27**	1.19	0.21	6.67	0.23	0.03	1.85
**04:05:04**	**02:01:01G**	**8.26E-19**	**1.47E-19**	**4.63E-18**	6.01	0.57	63.39	0.93	0.07	108.62

**04:05:04**	**03:01:01G**	**3.51**	**1.74**	**7.09**	**2.84**	**1.55**	**5.19**	**4.31E-17**	**4.63E-18**	**4.01E-16**
**04:05:04**	**03:02:01G**	**1.22E-14**	**1.43E-15**	**1.05E-13**	**2.98E-17**	**3.68E-18**	**2.41E-16**	**3.89E-16**	**4.88E-17**	**3.11E-15**
**04:05:04**	**04:02:01G**	0.93	0.18	4.73	2.18	0.88	5.38	0.44[a]	-0.64	1.53
**04:05:04**	**05:01:01G**	1.00	.	.	**4.24**	**1.53**	**11.69**	**3.48E-14**	**4.04E15**	**3.00E-13**
**04:06:01G**	**04:02:01G**	1.00	.	.	1.00	.	.	1.00	.	.
**04:07:01G**	**02:01:01G**	0.83	0.29	2.38	1.31	0.37	4.65	0.39	0.06	2.50
**04:07:01G**	**03:01:01G**	0.74	0.37	1.49	0.91	0.41	2.01	-0.51[a]	-1.07	0.03
**04:07:01G**	**03:02:01G**	0.84	0.50	1.39	1.06	0.55	2.03	1.55	0.87	2.75
**04:07:01G**	**03:02:03**	**2.25**	**1.23**	**4.11**	**1.19E-14**	**1.48E-15**	**9.63E-14**	**-1.22[a]**	**-1.66**	**-0.77**
**04:07:01G**	**04:02:01G**	0.46	0.06	3.17	0.83	0.09	7.45	**5.92**	**1.33**	**2.64**
**04:07:01G**	**05:01:01G**	0.69	0.24	2.00	**3.40**	**1.45**	**7.97**	185.42	0.72	475.10
**04:08:01**	**02:01:01G**	**2.69E-15**	**3.03E-16**	**2.39E-14**	**4.21**	**1.39**	**12.70**	0.21[a]	-1.11	1.53
**04:08:01**	**03:01:01G**	**2.69E-15**	**3.03E-16**	**2.39E-14**	**4.21**	**1.39**	**12.70**	0.21[a]	-1.11	1.53
**04:10:01G**	**03:01:01G**	1.00	.	.	1.00	.	.	1.00	.	.
**04:10:01G**	**04:02:01G**	1.00	.	.	1.00	.	.	1.00	.	.
**04:11:01**	**02:01:01G**	**7.47E-17**	**9.48E-18**	**5.89E-16**	**4.27E-14**	**2.42E-15**	**7.54E-13**	**8.96E-17**	**1.10E-17**	**7.32E-16**
**04:11:01**	**03:02:01G**	1.25	0.68	2.29	2.67	0.98	7.25	**1.63E-15**	**5.06E-16**	**5.25E-15**
**04:11:01**	**04:02:01G**	1.17	0.58	2.36	**6.36**	**2.35**	**17.22**	**1.17E-15**	**1.39E-16**	**9.89E-15**
**07:01:01G**	**02:01:01G**	0.89	0.44	1.78	1.71	0.83	3.54	1.18	0.57	2.44
**07:01:01G**	**03:01:01G**	0.83	0.09	7.28	2.60	0.61	11.09	1.10	0.32	3.78
**07:01:01G**	**03:02:01G**	1.02	0.37	2.78	2.36	0.74	7.54	1.28	0.42	386.15
**07:01:01G**	**03:03:02G**	-0.32[a]	-1.22	0.58	1.99	0.14	27.75	0.76	0.16	3.60
**07:01:01G**	**04:02:01G**	1.27[a]	-1.46	1.72	1.72	0.18	16.19	0.98	0.23	4.23
**07:01:01G**	**05:01:01G**	0.83	0.10	6.52	**9.31E-18**	**2.31E-18**	**3.75E-17**	0.74	0.08	6.28
**07:11**	**03:02:01G**	**7.47E-17**	**9.48E-18**	**5.89E-16**	**4.27E-14**	**2.42E-15**	**7.54E-13**	**8.96E-17**	**1.10E-17**	**7.32E-16**
**07:11**	**04:02:01G**	**7.84E-16**	**1.01E-16**	**6.08E-15**	**20.29**	**8.25**	**49.93**	**7.65**	**3.18**	**837.74**
**08:01:01G**	**03:01:01G**	0.40	0.04	4.06	**1.93E-16**	**4.17E-17**	**8.98E-16**	**4.34E-19**	**9.48E-20**	**1.98E-18**
**08:01:01G**	**04:02:01G**	0.40	0.04	4.06	1.00	0.18	5.51	**5.71E-16**	**1.58E-16**	**2.07E-15**
**08:02:01G**	**03:01:01G**	1.24	0.41	3.75	**8.17**	**1.32**	**50.47**	-0.16[a]	-1.74	1.41
**08:02:01G**	**04:02:01G**	1.10	0.38	3.15	1.51	0.52	4.33	0.12[a]	-0.54	0.02
**08:02:01G**	**05:01:01G**	1.00	.	.	**2.32E-15**	**2.64E-16**	**2.05E-14**	**7.52E-14**	**9.37E-15**	**6.04E-13**
**08:03:02**	**04:02:01G**	1.00	.	.	1.00	.	.	1.00	.	.
**08:04:01**	**03:01:01G**	1.49	0.24	9.16	0.73	0.15	3.59	**4.45E-19**	**1.18E-19**	**1.67E-18**
**08:04:01**	**03:02:01G**	**8.53**	**4.05**	**18.00**	**1.46E-16**	**3.36E-17**	**6.37E-16**	**1.36E-16**	**2.70E-17**	**6.87E-16**
**08:04:01**	**04:02:01G**	1.00	.	.	1.00	.	.	1.00	.	.
**08:04:01**	**05:01:01G**	**4.21**	**2.05**	**8.65**	**2.91**	**1.02**	**8.25**	**4.50E-19**	**4.81E-20**	**4.21E-18**
**08:06:06**	**02:01G**	1.00	.	.	1.00	.	.	1.00	.	.
**08:07**	**04:02:01G**	5.25	0.16	16.27	3.83E+18	.	.	**0.95**	**1.46**	**0.96**
**09:01:02G**	**03:01:01G**	1.91	0.69	5.29	**8.46**	**3.12**	**22.95**	**5.38E-15**	**1.17E-15**	**2.48E-14**
**09:01:02G**	**03:03:02G**	0.99	0.38	2.55	1.67	0.19	14,.39	0.86	0.19	3.79
**10:01:01G**	**05:01:01G**	0.62	0.23	1.61	1.78	0.63	5.04	2.92	0.80	1.06
**11:01:01G**	**03:01:01G**	0.54	0.17	1.76	0.85	0.24	2.99	-0.52[a]	-1.30	0.24
**11:01:01G**	**03:02:01G**	0.89	0.26	3.08	1.85	0.55	6.14	270.36	0.62	1.16
**11:01:02**	**05:01:01G**	1.00	.	.	1.00	.	.	1.00	.	.
**11:02:01**	**03:01:01G**	1.22	0.55	2.70	3.50	0.56	21.86	**2.56E-16**	**6.52E-17**	**1.01E-15**
**11:02:01**	**03:02:01G**	1.00	.	.	1.00	.	.	1.00	.	.

**11:02:01**	**04:02:01G**	1.00	.	.	1.00	.	.	1.00	.	.
**11:02:01**	**05:01:01G**	0.61	0.35	1.06	**9.46**	**4.48**	**19.98**	**8.97E-16**	**1.12E-16**	**7.17E-15**
**11:04:01G**	**02:01:01G**	**4.73E-15**	**7.81E-16**	**2.87E-14**	1.92	0.23	16.01	**7.54E-15**	**9.53E-16**	**5.96E-14**
**11:04:01G**	**03:01:01G**	0.50	0.22	1.10	0.68	0.15	3.07	-0.05[a]	-0.69	0.68
**11:04:01G**	**03:02:01G**	0.80	0.33	1.93	**1.11E-16**	**1.16E-17**	**1.07E-15**	2.67	0.33	2.15
**11:04:01G**	**04:02:01G**	1.00	.	.	1.00	.	.	**1.31E-14**	**1.51E-15**	**1.13E-13**
**11:04:01G**	**05:01:01G**	**1.33E-19**	**2.52E-20**	**7.03E-19**	**1.70E-14**	**1.85E-19**	**1.56E-17**	1.68	0.35	8.06
**11:04:01G**	**06:03:01G**	1.00	.	.	1.00	.	.	1.00	.	.
**11:14:01**	**04:02:01G**	1.24	0.41	3.75	**3.42**	**1.20**	**9.73**	**3.18E-15**	**3.79E-16**	**2.67E-14**
**12:01:01G**	**03:02:01G**	**4.80**	**2.66**	**8.64**	**6.92E-18**	**6.79E-19**	**7.05E-17**	**5.55E-19**	**1.13E-19**	**2.72E-18**
**12:01:01G**	**04:02:01G**	1.00	.	.	1.00	.	.	1.00	.	.
**13:01:01G**	**03:01:01G**	1.17	0.64	2.11	**2.09E-18**	**5.74E-19**	**7.58E-18**	**4.09E-16**	**9.32E-17**	**1.79E-15**
**13:01:01G**	**05:02:01G**	4.94E-20	.	.	**3.35E-17**	**4.13E-18**	**2.71E-18**	**1.64E-15**	**2.10E-16**	**1.28E-14**
**13:01:01G**	**05:03:01G**	1.00	.	.	1.00	.	.	1.00	.	.
**13:01:01G**	**06:02:01G**	1.00	.	.	**27.22**	**6.58**	**112.44**	**-0.78[a]**	**-1.22**	**-0.34**
**13:01:01G**	**06:03:01G**	1.30	0.61	2.79	0.27	0.05	1.35	139.14	0.65	2.93
**13:02:01G**	**03:01:01G**	0.44	0.13	1.52	2.91E-20	.	.	**4.34[a]**	**3.69**	**4.99**
**13:02:01G**	**05:01:01G**	**3.38E-15**	**4.41E-16**	**2.59E-14**	**1.23E-15**	**2.61E-16**	**5.79E-15**	**-1.29[a]**	**-1.74**	**-0.85**
**13:02:01G**	**06:02:01G**	**2.76E-19**	**3.47E-20**	**2.20E-18**	**2.90**	**1.04**	**8.07**	**-1.17[a]**	**-1.67**	**-0.67**
**13:02:01G**	**06:04:01G**	0.81	0.25	2.62	**4.87E-16**	**1.09E-16**	**2.17E-15**	0.56	0.07	4.18
**13:02:01G**	**06:09:01G**	0.29	0.02	3.67	0.36	0.05	2.31	1.18	0.21	6.55
**13:03:01G**	**03:01:01G**	1.40	0.53	3.70	**2.29E-16**	**4.88E-17**	**1.08E-15**	0.03[a]	-1.42	1.49
**13:03:01G**	**03:02:01G**	1.00	.	.	1.00	.	.	1.00	.	.
**13:03:01G**	**04:02:01G**	-0.25[a]	-0.67	0.16	1.98E-19	.	.	**4.54[a]**	**3.88**	**5.20**
**13:03:01G**	**05:01:01G**	1.00	.	.	1.00	.	.	**-1.33[a]**	**-1.82**	**-0.84**
**13:04**	**03:01:01G**	0.43	0.03	5.31	**2.75E-17**	**4.00E-18**	**1.90E-16**	**1.19E-14**	**2.24E-15**	**6.27E-14**
**13:04**	**04:02:01G**	**7.84E-16**	**3.56E-17**	**2.26E-15**	**1.92E-15**	**1.75E-16**	**2.12E-14**	**2.24E-16**	**2.51E-17**	**2.00E-15**
**13:05:01**	**03:01:01G**	**3.21**	**1.68**	**6.11**	8.44E-20	.	.	**4.19[a]**	**3.08**	**5.31**
**13:05:01**	**03:02:01G**	**3.21**	**1.68**	**6.11**	**4.66E-18**	**8.06E-19**	**2.70E-17**	**4.78[a]**	**3.65**	**5.92**
**14:01:01G**	**05:03:01G**	2.24	0.90	5.54	1.70	0.73	3.91	0.66	0.16	2.59
**14:01:01G**	**06:03:01G**	1.00	.	.	1.00	.	.	1.00	.	.
**14:02:01G**	**03:01:01G**	1.55	0.77	3.11	0.63	0.14	2.74	0.05[a]	-0.57	0.67
**14:02:01G**	**03:02:01G**	1.48	0.45	4.89	0.80	0.26	2.48	-0.40[a]	-1.12	0.31
**14:02:01G**	**04:02:01G**	1.00	.	.	**158.36**	**31.24**	**802.67**	-0.75[a]	-1.95	0.45
**14:02:01G**	**05:01:01G**	1.18	0.62	2.25	**5.67E-18**	**7.09E-19**	**4.54E-17**	**6.63E-15**	**8.29E-16**	**5.30E-14**
**14:02:01G**	**06:04:01G**	**1.69E-18**	**1.96E-19**	**1.46E-17**	9.35E-20	.	.	3.59[a]	2.92	4.27
**14:02:01G**	**06:09:01G**	1.00	.	.	1.00	.	.	1.00	.	.
**14:04:01**	**03:01:01G**	1.00	.	.	1.00	.	.	1.00	.	.
**14:06:01**	**06:03:01G**	1.00	.	.	1.00	.	.	1.00	.	.
**15:01:01G**	**05:01:01G**	0.89	0.25	3.11	1.14	0.27	4.76	0.34[a]	-0.84	1.52
**15:01:01G**	**05:02:01G**	**7.86E-15**	**8.94E-15**	**6.90E-14**	1.64	0.90	2.98	**-1.54[a]**	**-2.01**	**-1.08**
**15:01:01G**	**06:02:01G**	1.09	0.49	2.38	1.30	0.52	3.25	0.05[a]	-0.54	0.65
**15:01:01G**	**06:03:01G**	1.00	.	.	**27.22**	**6.58**	**112.44**	**-0.78[a]**	**-1.22**	**-0.34**
**15:01:01G**	**06:04:01G**	1.00	.	.	1.00	.	.	1.00	.	.
**15:02:01G**	**06:01:01G**	0.18[a]	-1.52	1.89	**2.52E-18**	**5.37E-19**	**1.18E-17**	0.82	0.08	8.36
**15:03:01G**	**06:02:01G**	**2.02**	**8.61**	**47.54**	**6.96E-18**	**1.39E-18**	**3.47E-17**	1.24	0.05	2.78

**16:01:01**	**03:01:01G**	**4.53**	**1.97**	**10.38**	**119.E-13**	**1.20E-14**	**1.18E-12**	**4.43E-14**	**5.34E-15**	**3.67E-13**
**16:01:01**	**05:02:01G**	0.92	0.17	4.89	0.64	0.09	4.29	0.97	0.17	5.53
**16:01:01**	**06:03:01G**	4.94E-20	.	.	**3.35E-17**	**4.13E-18**	**2.71E-16**	**1.64E-15**	**2.10E-16**	**1.28E-14**
**16:02:01G**	**03:01:01G**	1.15	0.59	2.24	0.68	0.22	2.05	0.85	0.36	1.97
**16:02:01G**	**03:02:01G**	0.40	0.10	1.64	0.71	0.16	3.14	-0.08[a]	-0.90	0.72
**16:02:01G**	**03:03:02G**	1.13	0.61	2.09	**12.15**	**4.57**	**32.34**	**4.26E-14**	**5.33E-15**	**3.41E-13**
**16:02:01G**	**04:02:01G**	**3.51**	**1.74**	**7.09**	**2.84**	**1.55**	**5.19**	**4.31E-17**	**4.63E-18**	**4.01E-16**
**16:02:01G**	**05:01:01G**	**5.54E-15**	**7.05E-16**	**4.36E-14**	1.53E-18	.	.	**-0.75[a]**	**-1.52**	**0.01**
**16:02:01G**	**05:02:01G**	0.58	0.05	6.79	**2.23E-15**	**3.10E-16**	**1.60E-14**	**4.05[a]**	**3.41**	**4.70**
**16:02:01G**	**05:03:01G**	1.00	.	.	1.00	.	.	1.00	.	.
**16:02:01G**	**06:03:01G**	0.83	0.43	1.59	**9.34E-17**	**2.06E-17**	**4.23E-16**	**6.35E-17**	**7.72E-18**	**5.22E-16**
**16:02:01G**	**06:04:01G**	1.00	**.**	**.**	1.00	.	.	1.00	.	.
**16:02:01G**	**06:09:01G**	**6.61E-14**	**6.63E-15**	**6.60E-13**	**7.99E-16**	**9.42E-17**	**26.78E-15**	**5.43E-15**	**6.40E-16**	**4.61E-14**

Analysis was adjusted for origin, age, lifetime amount of sexual partners, abortions, coinfection and viral load (low: ≤9.99E+5, medium: 1.00E+6 to 9.99E+9, high: ≥1.00E+10).

95%CI: 95% confidence interval, HPV: human papillomavirus, Coeff: regression coefficient (hazard ratio for Cox proportional hazards regression model and time ratio for log-normal parametric model), *DRB1: DR beta 1, DQB1: DQ beta 1*

Values in bold indicate statistical significance based on the 95%CI, $p<0.05$.

[a] The Cox proportional hazards model did not fulfil the the assumption of proportionality; the log-normal parametric model was used in such cases

Supplementary Table S8. Multivariate models evaluating the association between *DRB1* and *DQB1* alleles and infection clearance/persistence for HPV-33 -45 and -58

Haplotype		HPV-33			HPV-45			HPV-58		
DRB1	***DQB1***	**Coeff.**	**95%CI**		**Coeff.**	**95%CI**		**Coeff.**	**95%CI**	
**01:01:01G**	**03:01:01G**	**2.17E-16**	**2.07E-17**	**2.27E-15**	0.86	0.041	1.79	0.78	0.44	1.38
**01:01:01G**	**03:02:01G**	1.01	0.29	3.51	0.34	0.04	2.56	0.22[a]	-0.72	1.17
**01:01:01G**	**03:03:02G**	**17.09**	**4.51**	**64.70**	**36.89**	**9.94**	**136.91**	**1.43E-17**	**1.80E-18**	**1.13E-16**
**01:01:01G**	**04:02:01G**	**5.44E-17**	**5.31E-18**	**5.57E-16**	**2.01E-15**	**2.33E-16**	**1.73E-14**	**1.60E-13**	**2.03E-14**	**1.27E-12**
**01:01:01G**	**05:01:01G**	1.15	0.34	3.85	1.16	0.45	2.95	1.01	0.53	1.90
**01:02:01G**	**02:01:01G**	**11.69**	**2.76**	**4.94**	**3.66[a]**	**3.05**	**4.27**	**-0.68[a]**	**-1.09**	**.0.28**
**01:02:01G**	**03:01:01G**	0.01	.	.	**5.53**	**2.75**	**11.13**	**2.31**	**1.25**	**4.29**
**01:02:01G**	**03:02:01G**	1.00	.	.	**-0.77[a]**	**-1.12**	**-0.42**	0.79	0.03	1.63
**01:02:01G**	**03:03:02G**	5.02E-20	.	.	**6.14E-17**	**7.55E-18**	**4.99E-16**	**9.02E-19**	**1.15E-19**	**7.10E-18**
**01:02:01G**	**04:02:01G**	1.00	.	.	1.00	.	.	1.00	.	.
**01:02:01G**	**05:01:01G**	0.49	0.08	2.97	0.45[a]	-0.48	0.57	-0.19[a]	-0.61	0.23
**01:03:01**	**02:01:01G**	1.00	.	.	1.00	.	.	1.00	.	.
**01:03:01**	**03:01:01G**	1.43	0.26	7.68	2.24	0.53	9.32	-0.62[a]	-1.44	0.18
**01:03:01**	**03:02:01G**	1.00	.	.	**3.64**	**1.79**	**7.40**	**3.59***	**3.04**	**4.13**
**01:03:01**	**04:02:01G**	1.00	.	.	1.66	0.77	3.58	**5.97E-19**	**5.74E-20**	**6.20E-18**
**01:03:01**	**05:01:01G**	1.72	0.54	5.48	**2.91**	**1.42**	**5.98**	-0.50[a]	-1.23	0.21
**01:03:01**	**05:02:01G**	1.00	.	.	1.00	.	.	1.00	.	.
**03:01:01G**	**02:01:01G**	**3.49**	**1.31**	**9.25**	0.78	0.36	1.70	0.02[a]	-0.39	0.39
**03:01:01G**	**05:01:01G**	**11.69**	**2.76**	**4.94**	**1.94E-14**	**1.86E-15**	**2.02E-13**	**-0.52[a]**	**-0.89**	**-0.16**
**03:02:01**	**02:01:01G**	1.00	.	.	1.00	.	.	1.00	.	.
**03:02:01**	**04:02:01G**	**8.58**	**2.75**	**2.67**	5.53E-20	.	.	**3.51E-14**	**4.12E-15**	**2.98E-13**
**03:02:02**	**02:01:01G**	1.00	.	.	1.00	.	.	1.26	0.69	2.30
**03:02:02**	**03:01:01G**	3.16E-14	.	.	0.07[a]	-0.91	1.06	1.42	0.44	4.52
**04:01:01G**	**03:02:01G**	1.00	.	.	1.00	.	.	1.00	.	.
**04:02:01**	**02:01:01G**	**2.65**	**2.16**	**3.15**	**1.22E-14**	**1.53e-15**	**9.82E-14**	**2.89E-18**	**3.68E-19**	**2.27E-17**
**04:02:01**	**03:01:01G**	0.66	0.06	6.93	0.70	0.04	1.00	1.22E-19	.	.
**04:02:01**	**03:02:01G**	0.38	0.04	3.38	0.95	0.23	3.86	-0.03[a]	-1.11	1.04
**04:02:01**	**05:01:01G**	1.00	.	.	1.00	.	.	1.00	.	.
**04:03:01G**	**02:01:01G**	1.00	.	.	1.11	0.53	2.32	1.22	0.21	7.05
**04:03:01G**	**03:01:01G**	1.00	.	.	1.00	.	.	**7.53**	**2.97**	**1.91**
**04:03:01G**	**03:02:01G**	1.94E-19	.	.	2.17	0.60	7.85	**4.28**	**1.85**	**9.88**
**04:04:01**	**02:01:01G**	1.00	.	.	**2.01E-14**	**2.49E-15**	**1.63E-13**	**3.74[a]**	**3.19**	**4.28**
**04:04:01**	**03:01:01G**	6.15E-20	.	.	**4.17E-15**	**1.17E-15**	**1.49E-14**	1.03	0.19	5.33
**04:04:01**	**03:02:01G**	0.03	.	.	0.23[a]	-0.47	0.94	0.08[a]	-0.64	0.81
**04:04:01**	**05:01:01G**	1.00	.	.	**7.77E-17**	**9.90E-18**	**6.10E-16**	**2.05E-16**	**1.18E-17**	**3.57E-15**
**04:05:01**	**02:01:01G**	**134.73**	**10.84**	**1673.64**	**1.25E-16**	**2.48E-17**	**6.27E-16**	**3.79[a]**	**3.23**	**4.36**
**04:05:01**	**03:01:01G**	1.00	.	.	**5.37**	**2.17**	**13.28**	0.93	0.10	8.68
**04:05:01**	**03:02:01G**	0.06	-0.86	0.88	0.83	0.27	2.53	0.41[a]	-0.25	1.09
**04:05:04**	**02:01:01G**	0.03	.	.	**1.81E-17**	**2.20E-18**	**1.48E-16**	0.33	0.03	3.02

**04:05:04**	**03:01:01G**	1.00	.	.	**1.02E-13**	**6.26E-15**	**1.66E-12**	**3.22E-18**	**3.96E-19**	**2.61E-17**
**04:05:04**	**03:02:01G**	1.00	.	.	**5.68E-17**	**6.99E-18**	**4.61E-16**	-0.17 [a]	-0.36	0.32
**04:05:04**	**04:02:01G**	1.05	0.42	2.62	0.79	0.18	3.32	0.39	0.10	1.55
**04:05:04**	**05:01:01G**	1.00	.	.	1.66	0.77	3.58	**5.97E-19**	**5.74E-20**	**6.20E-18**
**04:06:01G**	**04:02:01G**	1.00	.	.	1.00	.	.	1.00	.	.
**04:07:01G**	**02:01:01G**	0.99	0.16	5.92	0.50	0.13	1.96	1.10	0.31	3.83
**04:07:01G**	**03:01:01G**	1.21	0.46	3.18	**0.45**	**0.21**	**0.95**	**-0.41 [a]**	**-0.76**	**-0.07**
**04:07:01G**	**03:02:01G**	1.06	0.48	2.33	0.83	0.48	1.42	**1.75**	**1.05**	**2.91**
**04:07:01G**	**03:02:03**	1.00	.	.	**1.48E-14**	**7.29E-16**	**3.02E-13**	**8.24E-14**	**9.61E-15**	**7.06E-13**
**04:07:01G**	**04:02:01G**	2.55	0.96	6.76	**2.49E-16**	**4.69E-17**	**1.33E-15**	0.08 [a]	-0.31	0.49
**04:07:01G**	**05:01:01G**	0.24	0.03	1.63	**2.66**	**1.00**	**7.02**	0.59	0.11	3.02
**04:08:01**	**02:01:01G**	0.41	0.14	1.18	0.77	0.38	1.52	2.12	0.44	10.25
**04:08:01**	**03:01:01G**	0.41	0.14	1.18	0.77	0.38	1.52	2.12	0.44	10.25
**04:10:01G**	**03:01:01G**	1.00	.	.	1.00	.	.	1.00	.	.
**04:10:01G**	**04:02:01G**	2.03E-19	.	.	**4.02E-14**	**4.79E-15**	**3.37E-13**	**6.45E-13**	**8.02E-14**	**5.19E-12**
**04:11:01**	**02:01:01G**	1.00	.	.	**4.18E-15**	**5.31E-16**	**3.28E-14**	**1.94E-16**	**2.29E-17**	**1.63E-15**
**04:11:01**	**03:02:01G**	0.06	.	.	**5.00E-19**	**1.42E-19**	**1.75E-18**	**9.36E-17**	**3.26E-17**	**2.68E-16**
**04:11:01**	**04:02:01G**	1.00	.	.	1.00	.	.	**2.40E-19**	**2.85E-20**	**2.02E-18**
**07:01:01G**	**02:01:01G**	0.59	0.17	1.94	0.81	0.38	1.70	0.97	0.49	1.90
**07:01:01G**	**03:01:01G**	0.41	0.14	1.18	1.02	0.58	1.80	-0.48 [a]	-1.10	0.13
**07:01:01G**	**03:02:01G**	0.44	0.03	6.04	0.53	0.13	2.17	1.58	0.77	3.25
**07:01:01G**	**03:03:02G**	1.30	0.09	1.86	2.28	0.14	3.48	0.91	0.38	2.20
**07:01:01G**	**04:02:01G**	0.03	.	.	1.57	0.50	4.93	0.55	0.11	2.63
**07:01:01G**	**05:01:01G**	1.30	0.09	1.86	0.22 [a]	-0.91	1.37	0.04 [a]	-0.08	0.96
**07:11**	**03:02:01G**	1.00	.	.	**4.18E-15**	**5.31E-16**	**3.28E-14**	**1.94E-16**	**2.29E-17**	**1.63E-15**
**07:11**	**04:02:01G**	1.00	.	.	1.00	.	.	**3.25 [a]**	**2.49**	**4.02**
**08:01:01G**	**03:01:01G**	1.00	.	.	**2.66**	**1.22**	**5.80**	0.84	0.32	2.18
**08:01:01G**	**04:02:01G**	1.05	0.42	2.62	0.63	0.07	5.13	0.31 [a]	-0.18	0.80
**08:02:01G**	**03:01:01G**	**20.31**	**4.38**	**94.17**	0.36	0.02	5.31	-0.41 [a]	-1.37	0.53
**08:02:01G**	**04:02:01G**	0.64	0.11	3.48	0.22	0.04	1.08	0.17 [a]	-0.35	0.70
**08:02:01G**	**05:01:01G**	**5.44E-17**	**5.31E-18**	**5.57E-16**	**2.01E-15**	**2.33E-16**	**1.73E-14**	**1.60E-13**	**2.03E-14**	**1.27E-12**
**08:03:02**	**04:02:01G**	1.00	.	.	1.00	.	.	1.00	.	.
**08:04:01**	**03:01:01G**	2.36E+18	.	.	-0.57 [a]	-1.38	0.23	**3.80**	**1.46**	**9.86**
**08:04:01**	**03:02:01G**	1.00	.	.	**2.14E-17**	**2.41E-18**	**1.89E-16**	**4.04**	**1.43**	**1.14**
**08:04:01**	**04:02:01G**	1.00	.	.	1.00	.	.	1.00	.	.
**08:04:01**	**05:01:01G**	1.00	.	.	**5.53**	**2.75**	**1.11**	**1.68**	**1.00**	**2.82**
**08:06:06**	**02:01G**	1.00	.	.	1.00	.	.	1.00	.	.
**08:07**	**04:02:01G**	**3.38E-18**	**3.62E-19**	**3.16E1-7**	**4.57E-17**	**5.28E-18**	**3.96E-16**	**-0.28 [a]**	**-0.51**	**0.05**
**09:01:02G**	**03:01:01G**	1.00	.	.	**23.73**	**7.92**	**71.10**	**3.27 [a]**	**2.76**	**3.77**
**09:01:02G**	**03:03:02G**	1.00	.	.	2.58	0.93	7.13	1.12	0.52	2.42
**10:01:01G**	**05:01:01G**	**3.78**	**1.50**	**9.55**	0.37	0.06	2.19	0.59	0.07	4.62
**11:01:01G**	**03:01:01G**	1.31	0.26	6.58	0.35	0.04	2.73	0.58 [a]	-0.43	1.59
**11:01:01G**	**03:02:01G**	**3.13**	**1.12**	**8.78**	0.58	0.06	5.21	0.01 [a]	-1.16	1.19
**11:01:02**	**05:01:01G**	1.00	.	.	1.00	.	.	1.00	.	.
**11:02:01**	**03:01:01G**	0.03	.	.	**1.96**	**1.08**	**3.59**	0.01 [a]	-0.64	0.68
**11:02:01**	**03:02:01G**	1.00	.	.	1.00	.	.	1.00	.	.

**11:02:01**	**04:02:01G**	1.00	.	.	1.00	.	.	1.00	.	.
**11:02:01**	**05:01:01G**	1.00	.	.	1.00	.	.	**3.96**	**1.69**	**9.27**
**11:04:01G**	**02:01:01G**	1.86	0.40	8.63	1.02	0.48	2.18	**5.97E-17**	**1.68E-17**	**2.13E-16**
**11:04:01G**	**03:01:01G**	0.73	0.23	2.26	0.70	0.27	1.81	0.49 [a]	-0.15	1.14
**11:04:01G**	**03:02:01G**	0.64	0.06	6.25	0.44	0.07	2.64	**-1.06 [a]**	**-1.50**	**-0.62**
**11:04:01G**	**04:02:01G**	1.00	.	.	**1.84E-17**	**2.26E-18**	**1.49E-16**	**1.53E-18**	**1.78E-19**	**1.32E-17**
**11:04:01G**	**05:01:01G**	0.35	0.10	1.26	0.45	0.09	2.21	0.80	0.39	1.63
**11:04:01G**	**06:03:01G**	1.86	0.40	8.63	1.25	0.60	2.59	**2.29E-15**	**2.77E-16**	**1.90E-14**
**11:14:01**	**04:02:01G**	1.00	.	.	**1.09E-16**	**1.39E-17**	**8.53E-16**	**4.07 [a]**	**3.35**	**4.80**
**12:01:01G**	**03:02:01G**	**3.58**	**1.33**	**9.58**	**12.65**	**4.93**	**32.45**	**2.75E-19**	**5.99E-20**	**1.26E-18**
**12:01:01G**	**04:02:01G**	1.00	.	.	1.00	.	.	1.00	.	.
**13:01:01G**	**03:01:01G**	0.02	.	.	**3.46**	**2.13**	**5.63**	**0.62 [a]**	**0.172**	**1.08**
**13:01:01G**	**05:02:01G**	1.00	.	.	1.45	0.76	2.75	1.37	0.62	3.02
**13:01:01G**	**05:03:01G**	1.00	.	.	1.00	.	.	1.00	.	.
**13:01:01G**	**06:02:01G**	1.00	.	.	**3.74**	**1.68**	**8.32**	-0.14 [a]	-0.48	0.20
**13:01:01G**	**06:03:01G**	0.74	0.30	1.85	**1.93**	**1.06**	**3.49**	0.07 [a]	-0.37	0.53
**13:02:01G**	**03:01:01G**	5.73E-19	.	.	1.38	0.52	3.66	1.62	0.52	4.98
**13:02:01G**	**05:01:01G**	0.56	0.06	4.65	**1.37E-15**	**1.73E-16**	**1.08E-14**	1.00	.	.
**13:02:01G**	**06:02:01G**	2.11	0.87	5.08	**5.35**	**2.39**	**1.19**	**-1.07 [a]**	**-1.96**	**-0.18**
**13:02:01G**	**06:04:01G**	2.26E-20	.	.	0.702	0.20	2.42	0.77	0.13	4.43
**13:02:01G**	**06:09:01G**	1.35	0.50	3.63	1.33	0.40	4.37	-0.53 [a]	-1.25	0.18
**13:03:01G**	**03:01:01G**	**10.94**	**2.66**	**44.99**	**22.15**	**8.33**	**58.85**	**-0.56 [a]**	**-1.13**	**-0.05**
**13:03:01G**	**03:02:01G**	1.00	.	.	1.00	.	.	1.00	.	.
**13:03:01G**	**04:02:01G**	1.00	.	.	1.00	.	.	1.28	0.78	2.08
**13:03:01G**	**05:01:01G**	**10.94**	**2.66**	**44.99**	**22.15**	**8.33**	**58.85**	**-1.07 [a]**	**-1.34**	**-0.80**
**13:04**	**03:01:01G**	1.00	.	.	**2.66**	**1.22**	**5.80**	**2.74 [a]**	**2.10**	**3.38**
**13:04**	**04:02:01G**	1.00	.	.	**2.66**	**1.22**	**5.80**	**2.74 [a]**	**2.10**	**3.38**
**13:05:01**	**03:01:01G**	1.00	.	.	1.25	0.12	1.23	**5.97E-16**	**9.02E-17**	**3.95E-15**
**13:05:01**	**03:02:01G**	1.00	.	.	**2.90E-15**	**3.28E-16**	**2.56E-14**	**3.24E-17**	**3.62E-18**	**2.91E-16**
**14:01:01G**	**05:03:01G**	**3.83E-16**	**4.52E-17**	**3.24E-15**	0.50 [a]	-0.32	1.34	0.02 [a]	-0.74	0.80
**14:01:01G**	**06:03:01G**	1.00	.	.	1.00	.	.	1.00	.	.
**14:02:01G**	**03:01:01G**	0.54	0.05	5.39	1.28	0.61	2.67	1.42	0.60	3.38
**14:02:01G**	**03:02:01G**	8.97E-20	.	.	**0.36**	**0.13**	**0.86**	1.43	0.42	4.78
**14:02:01G**	**04:02:01G**	1.43	0.08	2.32	3.23	0.24	4.34	**-0.95 [a]**	**-1.30**	**-0.59**
**14:02:01G**	**05:01:01G**	1.00	.	.	1.00	.	.	**2.77 [a]**	**2.17**	**3.36**
**14:02:01G**	**06:04:01G**	5.73E-19	.	.	1.32	0.57	3.06	**-0.59 [a]**	**-0.92**	**-0.25**
**14:02:01G**	**06:09:01G**	1.00	.	.	1.00	.	.	1.00	.	.
**14:04:01**	**03:01:01G**	1.00	.	.	1.00	.	.	1.00	.	.
**14:06:01**	**06:03:01G**	1.00	.	.	1.00	.	.	1.00	.	.
**15:01:01G**	**05:01:01G**	0.38	0.01	7.49	0.86	0.09	7.54	0.33 [a]	-0.50	1.18
**15:01:01G**	**05:02:01G**	**8.58**	**2.75**	**2.67**	5.53E-20	.	.	**3.51E-14**	**4.12E-15**	**2.98E-13**
**15:01:01G**	**06:02:01G**	**2.35**	**1.07**	**5.18**	-0.29 [a]	-0.55	0.09	-0.03 [a]	-0.44	0.37
**15:01:01G**	**06:03:01G**	1.00	.	.	**3.74**	**1.68**	**8.32**	-0.14 [a]	-0.48	0.20
**15:01:01G**	**06:04:01G**	1.00	.	.	1.00	.	.	1.00	.	.
**15:02:01G**	**06:01:01G**	1.49E-20	.	.	-0.14 [a]	-0.97	0.67	-0.24 [a]	-0.98	0.49
**15:03:01G**	**06:02:01G**	**4.15E-17**	**6.69E-18**	**2.57E-16**	2.04	0.20	2.02	1.68	0.29	9.57

**16:01:01**	**03:01:01G**	1.00	.	.	**9.04**	**3.92**	**2.08**	**2.30** [a]	**1.50**	**3.09**
**16:01:01**	**05:02:01G**	0.01	.	.	-0.13 [a]	-0.71	0.44	-0.80 [a]	-0.79	0.62
**16:01:01**	**06:03:01G**	1.00	.	.	1.45	0.76	2.75	1.37	0.62	3.02
**16:02:01G**	**03:01:01G**	1.60	0.57	4.46	1.96	0.98	3.92	1.17	0.60	2.27
**16:02:01G**	**03:02:01G**	0.83	0.27	2.51	0.72	0.28	1.85	**-0.56** [a]	**-0.89**	**-0.23**
**16:02:01G**	**03:03:02G**	1.00	.	.	1.00	.	.	**2.83E-15**	**3.65E-16**	**2.20E-14**
**16:02:01G**	**04:02:01G**	1.00	.	.	**1.02E-13**	**6.26E-15**	**1.66E-12**	**3.22E-18**	**3.96E-19**	**2.61E-17**
**16:02:01G**	**05:01:01G**	1.00	.	.	**-1.15** [a]	**-1.58**	**-0.73**	0.89	0.41	1.95
**16:02:01G**	**05:02:01G**	1.00	.	.	2.16	0.23	1.98	**3.07** [a]	**2.50**	**3.64**
**16:02:01G**	**05:03:01G**	1.00	.	.	1.00	.	.	1.00	.	.
**16:02:01G**	**06:03:01G**	1.00	.	.	**3.83**	**1.86**	**7.88**	**8.62E-18**	**9.52E-19**	**7.80E-17**
**16:02:01G**	**06:04:01G**	1.00	.	.	1.00	.	.	1.00	.	.
**16:02:01G**	**06:09:01G**	1.00	.	.	**6.80E-18**	**8.01E-19**	**5.77E-17**	2.21	0.45	1.08

Analysis was adjusted for origin, age, lifetime amount of sexual partners, abortions, coinfection and viral load (low: ≤9.99E+5, medium: 1.00E+6 to 9.99E+9, high: ≥1.00E+10).
95%CI: 95% confidence interval, HPV: human papillomavirus, Coeff: regression coefficient (hazard ratio for Cox proportional hazards regression model and time ratio for log-normal parametric model), *DRB1: DR beta 1, DQB1: DQ beta 1*
Values in bold indicate statistical significance based on the 95%CI, $p<0.05$.
[a] The Cox proportional hazards model did not fulfil the the assumption of proportionality;; the log-normal parametric model was used in such cases

Supplementary Table S9. Multivariate models evaluating the association between *DRB1* and *DQB1* alleles and HR-HPV redetection

**Alelo**	**HPV-16**			**HPV-18**			**HPV-31[a]**			**HPV-45[a]**			**HPV-58[a]**		
	**Coeff.**	**95%CI**		**Coeff.**	**95%CI**		**Coeff.**	**95%CI**		**Coeff.**	**95%CI**		**Coeff.**	**95%CI**	
DRB1															
**01:01:01G**	1.26	0.49	3.19	**2.88**	**1.34**	**6.17**	0.21	-0.11	0.52	0.15	-0.34	0.64	0.16	-0.34	0.67
**01:02:01G**	0.76	0.28	2.05	0.63	0.28	1.39	**-0.37**	**-0.68**	**-0.06**	-0.14	-0.57	0.28	-0.16	-0.66	0.34
**01:03:01**	**2.79**	**1.56**	**5.02**	**4.87E-15**	**5.84E-16**	**4.06E-14**	0.23	-0.06	0.52	**3.31**	**2.82**	**3.80**	0.22[b]	0.02	2.87
**03:01:01G**	1.01	0.51	2.01	2.01	0.89	4.54	0.06	-0.30	0.42	**-0.42**	**-0.79**	**-0.05**	0.23	-0.39	0.85
**03:02:01**	-0.33[a]	-0.69	0.03	**7.21E-16**	**8.94E-17**	**5.82E-15**	**2.63**	**2.24**	**3.02**	**-0.41**	**-0.68**	**-0.14**	-0.38	-0.84	0.07
**03:02:02**	0.20[a]	-1.14	1.53	2.88E-20	.	.	**1.92**	**1.55**	**2.30**	-0.29	-0.73	0.15	**1.81**	**1.05**	**2.57**
**04:01:01G**	-	.	.	-	.	.	0.00	.	.	-	.	.	-	.	.
**04:02:01**	-0.22[a]	-0.57	0.12	-0.28[a]	-0.63	0.07	**-0.45**	**-0.66**	**-0.23**	0.37	-0.33	1.07	0.00	-0.47	0.46
**04:03:01G**	-0.05[a]	-0.58	0.48	**3.28**	**1.15**	**9.33**	-0.15	-0.71	0.41	0.08	-0.36	0.53	**2.74**	**2.12**	**3.36**
**04:04:01**	0.92	0.50	1.70	2.42	0.94	6.18	0.20	-0.27	0.67	-0.30	-0.80	0.20	-0.23	-0.90	0.44
**04:05:01**	1.08	0.47	2.47	1.69	0.85	3.34	-0.40	-0.96	0.17	-0.27	-0.95	0.40	-0.01	-0.59	0.56
**04:05:04**	-0.10[a]	-0.60	0.40	0.23	0.05	1.16	-0.12	-0.71	0.47	-0.01	-0.70	0.68	**2.92**	**2.24**	**3.60**
**04:06:01G**	-	.	.	-	.	.	0.00	.	.	-	.	.	-	.	.
**04:07:01G**	1.36	0.81	2.28	0.89	0.51	1.58	0.14	-0.12	0.40	-0.09	-0.36	0.18	0.07	-0.27	0.41
**04:08:01**	**32.15**	**8.08**	**127.87**	1.05E-19	.	.	**2.13**	**1.73**	**2.52**	**2.27**	**1.78**	**2.77**	**3.47**	**2.98**	**3.96**
**04:10:01G**	-	.	.	-	.	.	0.00	.	.	-	.	.	-	.	.
**04:11:01**	0.37[a]	-0.18	0.92	0.56	0.23	1.36	**-0.67**	**-0.97**	**-0.38**	**-0.41**	**-0.75**	**-0.07**	**-0.41**	**-0.77**	**-0.05**
**07:01:01G**	1.19	0.64	2.21	0.34	0.10	1.15	0.02	-0.26	0.30	0.30	-0.15	0.76	-0.06	-0.52	0.41
**07:11**	-	.	.	1.22	0.40	3.72	-0.32	-0.96	0.31	-0.21	-0.55	0.12	-0.15	-0.83	0.54
**08:01:01G**	2.02	0.19	21.17	1.41	0.25	7.96	**-0.71**	**-0.99**	**-0.42**	**2.83**	**2.31**	**3.35**	**2.05**	**1.40**	**2.70**
**08:02:01G**	0.92	0.35	2.41	0.65	0.23	1.80	-0.21	-0.54	0.12	**-0.52**	**-0.80**	**-0.23**	3.27[b]	0.93	1.15
**08:03:02**	-	.	.	-	.	.	0.00	.	.	-	.	.	-	.	.
**08:04:01**	0.11[a]	-0.33	0.55	3.57	0.55	23.13	**-0.62**	**-0.97**	**-0.27**	0.10	-0.72	0.93	**2.85**	**2.28**	**3.43**
**08:06**	-	.	.	-	.	.	0.00	.	.	-	.	.	-	.	.
**08:07**	**0.45[a]**	**0.18**	**0.71**	**4.56E-15**	**5.50E-16**	**3.78E-14**	0.20	-0.17	0.57	-	.	.	**2.52**	**1.90**	**3.14**
**09:01:02G**	0.91	0.23	3.59	1.03	0.32	3.33	0.10	-0.28	0.48	**0.52**	**0.12**	**0.92**	-0.21	-0.72	0.31
**10:01:01G**	1.15	0.43	3.11	**0.42**	**0.20**	**0.86**	-0.17	-0.53	0.19	**-0.49**	**-0.83**	**-0.16**	-0.02	-0.75	0.71
**11:01:01G**	**2.82**	**1.15**	**6.87**	1.15	0.24	5.39	**2.73**	**2.34**	**3.13**	-0.21	-0.63	0.22	-0.48	-1.40	0.44
**11:01:02**	-	.	.	-	.	.	0.00	.	.	-	.	.	-	.	.
**11:02:01**	0.91	0.34	2.46	1.10E-19	.	.	**0.75**	**0.29**	**1.21**	0.07	-0.16	0.31	**2.65**	**1.72**	**3.58**
**11:04:01G**	1.19	0.58	2.46	**0.17**	**0.06**	**0.45**	0.02	-0.32	0.36	**0.55**	**0.24**	**0.86**	-0.10	-0.63	0.43

**11:14:01**	1.55	0.51	4.67	8.94E-20	.	.	**-0.71**	**-0.99**	**-0.42**	**-0.88**	**-1.32**	**-0.43**	**-0.81**	**-1.35**	**-0.26**
**12:01:01G**	4.31E-20	.	.	**2.58**	**1.30**	**5.13**	**-0.41**	**-0.67**	**-0.14**	**2.85**	**2.33**	**3.36**	**-0.55**	**-1.03**	**-0.06**
**13:01:01G**	-0.11[a]	-0.35	0.14	**2.00**	**1.18**	**3.41**	0.11	-0.21	0.43	0.22	-0.20	0.63	-0.32	-0.80	0.16
**13:02:01G**	1.44	0.39	5.26	1.52	0.46	5.02	0.34	-0.41	1.08	-0.01	-0.43	0.41	-0.07	-0.44	0.30
**13:03:01G**	1.56E-19	.	.	**3.55**	**1.09**	**11.59**	-0.03	-0.67	0.61	**2.38**	**1.76**	**3.00**	**2.42**	**1.84**	**2.99**
**13:04**	-0.14[a]	-1.04	0.77	**-0.69[a]**	**-0.96**	**-0.41**	**0.48**	**0.05**	**0.92**	**2.83**	**2.31**	**3.35**	**0.64**	**0.01**	**1.26**
**13:05:01**	2.60E-19	.	.	**2.58**	**1.30**	**5.13**	**-0.60**	**-1.13**	**-0.07**	**2.50**	**2.00**	**3.00**	**0.82**	**0.25**	**1.39**
**14:01:01G**	3.98E-21	.	.	0.51	0.10	2.57	-0.11	-0.47	0.25	**-0.55**	**-1.02**	**-0.07**	-0.57	-1.29	0.15
**14:02:01G**	0.49	0.18	1.34	**2.12**	**1.04**	**4.34**	0.03	-0.32	0.39	0.26	-0.03	0.56	0.30	-0.19	0.80
**14:04:01**	-	.	.	-	.	.	0.00	.	.	-	.	.	-	.	.
**14:06:01**	-	.	.	-	.	.	0.00	.	.	-	.	.	-	.	.
**15:01:01G**	0.76	0.37	1.59	0.67	0.25	1.78	-0.02	-0.35	0.30	0.14	-0.19	0.46	-0.04	-0.45	0.38
**15:02:01G**	1.01	0.35	2.87	**3.66**	**1.56**	**8.60**	**2.73**	**2.19**	**3.28**	0.34	-0.63	1.32	**2.76**	**2.14**	**3.38**
**15:03:01G**	**0.39[a]**	**0.12**	**0.67**	**-0.74[a]**	**-1.13**	**-0.35**	**0.36**	**0.10**	**0.62**	**2.16**	**1.51**	**2.81**	**3.26**	**2.76**	**3.76**
**16:01:01**	-0.30[a]	-0.83	0.22	**4.42**	**1.94**	**10.10**	**0.57**	**0.15**	**1.00**	**2.74**	**2.31**	**3.18**	**3.10**	**2.46**	**3.75**
**16:02:01G**	0.19[a]	-0.16	0.54	0.63	0.24	1.67	-0.05	-0.44	0.34	-0.06	-0.55	0.42	**0.48**	**0.04**	**0.92**
DQB1															
**02:01:01G**	1.07	0.59	1.94	1.35	0.64	2.84	0.02	-0.25	0.28	-0.12	-0.42	0.18	0.10	-0.35	0.55
**03:01:01G**	0.76	0.45	1.26	0.79	0.42	1.50	-0.03	-0.25	0.18	0.09	-0.17	0.35	0.52[b]	0.22	1.22
**03:02:01G**	1.26	0.75	2.12	1.33	0.81	2.20	0.01	-0.22	0.25	-0.14	-0.40	0.13	0.01	-0.29	0.32
**03:02:03**	**2.87**	**1.39**	**5.89**	-	.	.	0.12	-0.16	0.40	**-0.72**	**-1.05**	**-0.39**	**-1.01**	**-1.47**	**-0.54**
**03:03:02G**	0.21[a]	-0.20	0.62	0.38	0.07	2.19	-3.48E-03	-0.34	0.33	**0.52**	**0.09**	**0.94**	-0.15	-0.54	0.24
**04:02:01G**	1.06	0.53	2.11	**0.41**	**0.17**	**0.98**	-0.11	-0.39	0.16	**-0.36**	**-0.66**	**-0.06**	-0.16	-0.54	0.23
**05:01:01G**	1.15	0.66	2.03	0.76	0.39	1.46	0.02	-0.21	0.25	0.10	-0.20	0.41	-0.03	-0.37	0.31
**05:02:01G**	**-0.39[a]**	**-0.72**	**-0.06**	**2.35**	**1.06**	**5.19**	0.31	-0.30	0.92	0.19	-0.41	0.78	-0.03	-0.48	0.43
**05:03:01G**	3.98E-21	.	.	0.51	0.10	2.57	-0.11	-0.47	0.25	**-0.55**	**-1.02**	**-0.07**	-0.57	-1.29	0.15
**06:01:01G**	1.01	0.35	2.87	**3.66**	**1.56**	**8.60**	**2.73**	**2.19**	**3.28**	0.34	-0.63	1.32	**2.76**	**2.14**	**3.38**
**06:02:01G**	0.25[a]	-0.05	0.54	1.28	0.47	3.51	-0.06	-0.36	0.24	0.19	-0.14	0.53	0.29	-0.23	0.82
**06:03:01G**	-0.11[a]	-0.35	0.14	**2.00**	**1.18**	**3.41**	0.11	-0.21	0.43	0.23	-0.18	0.63	-0.36	-0.79	0.07
**06:04:01G**	1.11	0.27	4.55	0.94	0.52	1.71	**2.71**	**2.13**	**3.29**	-0.13	-0.56	0.30	-0.25	-0.53	0.04
**06:09:01G**	-0.36[a]	-1.02	0.29	4.23	0.38	46.71	0.10	-0.63	0.83	0.06	-0.55	0.67	0.01	-0.58	0.59

Analysis was adjusted for origin, age, lifetime amount of sexual partners, abortions, coinfection and viral load (low: ≤9.99E+5, medium: 1.00E+6 to 9.99E+9, high: ≥1.00E+10).
95%CI: 95% confidence interval, HPV: human papillomavirus, Coeff: regression coefficient (hazard ratio for Cox proportional hazards regression model and time ratio for log-normal parametric model), *DRB1: DR beta 1, DQB1: DQ beta 1,*
Values in bold indicate statistical significance based on the 95% confidence interval, $p<0.05$.
[a] The Cox proportional hazards model did not fulfil the assumption of proportionality; the log-normal parametric model was used in such cases
[b] The Cox proportional hazards model was used.

Supplementary Table S10. Multivariate models evaluating the association between *DRB1* and *DQB1* haplotypes and HR-HPV redetection for HPV-16, -18 and -31

Haplotype		HPV-16			HPV-18			HPV-31		
DRB1	***DQB1***	**Coeff.**	**95%CI**		**Coeff.**	**95%CI**		**Coeff.**	**95%CI**	
01:01:01G	03:01:01G	**1.55**	**1.27**	**1.82**	**2.74**	**1.07**	**6.98**	0.47[b]	0.09	2.39
01:01:01G	03:02:01G	-0.32	-0.82	-0.17	2.23	0.23	2.09	**4.73E-18[b]**	**1.01E-18**	**2.21E-17**
01:01:01G	03:03:02G	0	.	.	1.00	.	.	1.00[b]	.	.
01:01:01G	04:02:01G	0	.	.	1.00	.	.	0	.	.
01:01:01G	05:01:01G	-0.13	-0.39	-0.12	1.97	0.96	4.04	0.30	-0.02	0.62
01:02:01G	02:01:01G	0	.	.	**7.82E-17**	**5.93E-18**	**1.03E-15**	**3.84[b]**	**1.92**	**7.68**
01:02:01G	03:01:01G	**0.23**	**0.04**	**0.42**	1.17E-19	.	.	1.00[b]	.	.
01:02:01G	03:02:01G	0.16	-0.35	0.68	1.51	0.23	9.80	2.87[b]	0.88	9.27
01:02:01G	03:03:02G	**1.40**	**1.12**	**1.68**	**2.74E-14**	**3.29E-15**	**2.28E-13**	**-0.38**	**-0.64**	**-0.12**
01:02:01G	04:02:01G	0	.	.	1.00			1.00[b]	.	.
01:02:01G	05:01:01G	0.14	-0.09	0.39	1.16	0.50	2.68	**3.43[b]**	**1.57**	**7.48**
01:03:01	02:01:01G	0	.	.	**5.54E-16**	**6.46E-17**	**4.75E-15**	**0.32**	**0.06**	**0.59**
01:03:01	03:01:01G	**-0.51**	**-0.69**	**-0.33**	1.00	.	.	0.86[b]	0.18	4.10
01:03:01	03:02:01G	-0.09	-0.26	0.07	**8.50E-16**	**8.91E-17**	**8.11E-15**	1.00[b]	.	.
01:03:01	04:02:01G	0	.	.	**1.47E-16**	**1.80E-17**	**1.20E-15**	0	.	.
01:03:01	05:01:01G	**-0.30**	**-0.60**	**-0.05**	9.59E-20	.	.	0.23[b]	-0.05	0.51
01:03:01	05:02:01G	0	.	.	1.00	.	.	0	.	.
03:01:01G	02:01:01G	-0.04	-0.20	0.20	1.45	0.69	3.05	0.05	-0.30	0.41
03:01:01G	05:01:01G	0	.	.	1.00	.	.	**3.94**	**1.49**	**1.04**
03:02:01	02:01:01G	0	.	.	1.00	.	.	0	.	.
03:02:01	04:02:01G	**-0.39**	**-0.62**	**-0.16**	**1.40E-15**	**1.80E-16**	**1.08E-14**	**2.63**	**2.24**	**3.02**
03:02:02	02:01:01G	0	.	.	**8.23E-15**	**7.21E-16**	**9.41E-14**	**1.87**	**1.36**	**2.37**
03:02:02	03:01:01G	0.13	0.006	2.59	**4.23E-20**	.	.	**1.92**	**1.55**	**2.29**
04:01:01G	03:02:01G	0	.	.	1.00	.	.	0	.	.
04:02:01	02:01:01G	0	.	.	1.00	.	.	**0.60**	**0.19**	**1.01**

**04:02:01**	**03:01:01G**	0.13	-0.15	0.42	1.00	.	.	**0.43**	**-0.68**	**-0.19**
**04:02:01**	**03:02:01G**	-0.08	-0.28	0.11	-0.21[a]	-0.46	0.03	**0.44**	**-0.66**	**-0.20**
**04:02:01**	**05:01:01G**	0	.	.	1.00	.	.	0	.	.
**04:03:01G**	**02:01:01G**	0.01	-0.15	0.18	**3.03**	**1.12**	**8.16**	**1.98**	**1.58**	**2.38**
**04:03:01G**	**03:01:01G**	0	.	.	**5.98E-16**	**6.44E-17**	**5.55E-15**	**-0.67**	**-1.17**	**-0.18**
**04:03:01G**	**03:02:01G**	0.10	-0.12	0.32	**3.86**	**1.58**	**9.40**	-0.34	-0.91	0.23
**04:04:01**	**02:01:01G**	**0.30**	**0.15**	**0.46**	1.00	.	.	0	.	.
**04:04:01**	**03:01:01G**	0.13	-0.28	0.55	1.00	0.41	2.49	0.02	-0.49	0.48
**04:04:01**	**03:02:01G**	0.04	-0.11	0.21	-0.27[a]	-0.56	0.02	0.20	-0.26	0.66
**04:04:01**	**05:01:01G**	0	.	.	**-1.02[a]**	**-1.24**	**-0.80**	0	.	.
**04:05:01**	**02:01:01G**	-0.19	-0.83	0.43	**5.01**	**1.85**	**1.35**	**1.81**	**1.37**	**2.26**
**04:05:01**	**03:01:01G**	**1.58**	**1.28**	**1.88**	2.44	0.98	6.11	**0.54**	**0.16**	**0.91**
**04:05:01**	**03:02:01G**	-0.004	-0.25	0.25	**3.02**	**1.25**	**7.29**	0.39	-0.96	0.16
**04:05:04**	**02:01:01G**	**-0.44**	**-0.68**	**-0.20**	**1.21E-17**	**1.43E-18**	**1.02E-16**	**0.32**	**0.02**	**0.63**
**04:05:04**	**03:01:01G**	**1.61**	**1.34**	**1.88**	**2.78E-14**	**3.58E-15**	**2.15E-13**	0	.	.
**04:05:04**	**03:02:01G**	-0.23	-0.52	0.05	1.00	.	.	0	.	.
**04:05:04**	**04:02:01G**	-0.13	-0.43	0.16	0.63	0.124	3.19	0.12	-0.71	0.46
**04:05:04**	**05:01:01G**	0	.	.	**1.47E-16**	**1.80E-17**	**1.20E-15**	0	.	.
**04:06:01G**	**04:02:01G**	0	.	.	1.00	.	.	0	.	.
**04:07:01G**	**02:01:01G**	**0.38**	**0.20**	**0.55**	0.63	0.148	2.70	-0.12	-1.17	0.92
**04:07:01G**	**03:01:01G**	-0.03	-0.20	0.13	0.22	0.03	1.26	0.19	-0.32	0.72
**04:07:01G**	**03:02:01G**	-0.04	-0.17	0.07	0.84	0.45	1.57	0.07	-0.19	0.33
**04:07:01G**	**03:02:03**	**-0.16**	**-0.32**	**-0.006**	**7.97E-15**	**9.98E-16**	**6.37E-14**	0.12	-0.15	0.39
**04:07:01G**	**04:02:01G**	**-0.29**	**-0.48**	**-0.11**	1.37E-19	.	.	0.31	-0.14	0.78
**04:07:01G**	**05:01:01G**	-0.02	-0.29	0.24	0.94	0.33	2.63	-0.12	-0.54	0.29
**04:08:01**	**02:01:01G**	**-0.47**	**-0.73**	**-0.20**	**5.46E-17**	**1.09E-17**	**2.75E-16**	**2.12**	**1.73**	**2.52**
**04:08:01**	**03:01:01G**	**-0.47**	**-0.73**	**-0.20**	**5.46E-17**	**1.09E-17**	**2.75E-16**	**2.12**	**1.73**	**2.52**
**04:10:01G**	**03:01:01G**	0	.	.	1.00	.	.	0	.	.
**04:10:01G**	**04:02:01G**	0	.	.	1.00	.	.	0	.	.
**04:11:01**	**02:01:01G**	0	.	.	**2.76**	**1.26**	**6.01**	**0.67**	**-0.97**	**-0.37**
**04:11:01**	**03:02:01G**	0.28	-0.08	0.66	1.00	0.46	2.16	**0.67**	**-0.96**	**-0.37**

**04:11:01**	**04:02:01G**	**1.54**	**1.20**	**1.87**	3.68E-19	.	.	0.37	-0.05	0.80
**07:01:01G**	**02:01:01G**	-0.09	-0.28	0.09	**0.30**	**0.10**	**0.83**	0.16	-0.13	0.46
**07:01:01G**	**03:01:01G**	**1.30**	**1.01**	**1.59**	**5.69E-16**	**1.81E-16**	**1.79E-15**	0.14	-0.23	0.52
**07:01:01G**	**03:02:01G**	**0.24**	**0.005**	**0.48**	0.90	0.13	5.80	**2.58**	**2.18**	**2.97**
**07:01:01G**	**03:03:02G**	-0.09	-0.47	0.28	1.09	0.31	3.76	-0.14	-0.44	0.15
**07:01:01G**	**04:02:01G**	**-0.31**	**-0.55**	**-0.07**	7.26E-20	.		**0.39**	**0.10**	**0.68**
**07:01:01G**	**05:01:01G**	**1.40**	**1.12**	**1.68**	**2.02E-15**	**4.94E-16**	**8.27E-15**	-0.20	-0.61	0.20
**07:11**	**03:02:01G**	0			**2.76**	**1.26**	**6.01**	**7.29**	**0.97**	**-0.37**
**07:11**	**04:02:01G**	0			**4.25E-15**	**5.03E-16**	**3.59E-14**	**16.70**	**1.71**	**26.19**
**08:01:01G**	**03:01:01G**	0.12	-0.27	0.53	1.44	0.51	4.08	**-0.70**	**-0.99**	**-0.42**
**08:01:01G**	**04:02:01G**	0.12	-0.27	0.53	1.05	0.28	3.89	**-0.70**	**-0.99**	**-0.42**
**08:02:01G**	**03:01:01G**	**0.31**	**0.02**	**0.60**	3.91E-19	.	.	-0.28	-0.96	0.38
**08:02:01G**	**04:02:01G**	-0.02	-0.27	0.21	1.08	0.41	2.82	-0.21	-0.54	0.12
**08:02:01G**	**05:01:01G**	0	.	.	1.00	.	.	0	.	.
**08:03:02**	**04:02:01G**	0	.	.	1.00	.	.	0	.	.
**08:04:01**	**03:01:01G**	0.02	-0.19	0.23	0.67	0.13	3.26	**0.61**	**-0.96**	**-0.26**
**08:04:01**	**03:02:01G**	**0.19**	**0.05**	**0.33**	**1.51E-19**	**2.71E-20**	**8.37E-19**	**0.61**	**-0.97**	**-0.26**
**08:04:01**	**04:02:01G**	0	.	.	1.00	.	.	0	.	.
**08:04:01**	**05:01:01G**	0.16	-0.004	0.32	6.48E-19	.	.	0.37	-0.19	0.94
**08:06:06**	**02:01G**	0	.	.	1.00	.	.	0	.	.
**08:07**	**04:02:01G**	0.89	0.77.	2.56	2.46E-19	.	.	0.30	-1.65	0.57
**09:01:02G**	**03:01:01G**	0.08	-0.15	0.31	1.76	0.10	2.92	**0.60**	**0.24**	**0.96**
**09:01:02G**	**03:03:02G**	-0.05	-0.49	0.38	0.80	0.17	3.80	0.10	-0.27	0.48
**10:01:01G**	**05:01:01G**	-0.001	-0.38	0.38	0.60	0.26	1.38	-0.17	-0.53	0.18
**11:01:01G**	**03:01:01G**	-0.11	-0.26	0.03	0.30	0.09	0.97	**2.73**	**2.33**	**3.12**
**11:01:01G**	**03:02:01G**	-0.06	-0.25	0.12	0.66	0.09	4.80	**2.61**	**2.22**	**3.00**
**11:01:02**	**05:01:01G**	0	.	.	1.00	.	.	0	.	.
**11:02:01**	**03:01:01G**	0.17	-0.03	0.38	**1.62E-16**	**1.95E-17**	**1.34E-15**	**0.74**	**0.29**	**1.20**
**11:02:01**	**03:02:01G**	0	.	.	1.00	.	.	0	.	.
**11:02:01**	**04:02:01G**	0	.	.	1.00	.	.	0	.	.
**11:02:01**	**05:01:01G**	**1.40**	**1.11**	**1.68**	**1.62E-16**	**1.95E-17**	**1.34E-15**	**0.56**	**0.12**	**1.00**

**11:04:01G**	**02:01:01G**	0	.	.	**7.10E-16**	**1.12E-16**	**4.52E-15**	0.36	-0.07	0.80
**11:04:01G**	**03:01:01G**	-0.06	-0.24	0.12	**0.30**	**0.09**	**0.97**	0.02	-0.32	0.36
**11:04:01G**	**03:02:01G**	0.04	-0.18	0.27	1.00	.	.	-0.16	-0.68	0.35
**11:04:01G**	**04:02:01G**	0	.	.	1.00	.	.	**0.53**	**0.08**	**0.97**
**11:04:01G**	**05:01:01G**	**-0.51**	**-0.69**	**-0.33**	**6.90E-18**	**5.33E-19**	**8.94E-17**	-0.02	-0.38	0.33
**11:04:01G**	**06:03:01G**	0	.	.	1.00	.	.	0	.	.
**11:14:01**	**04:02:01G**	**0.31**	**0.02**	**0.60**	3.65E-19	.	.	**0.70**	**-0.99**	**-0.42**
**12:01:01G**	**03:02:01G**	**1.47**	**1.19**	**1.75**	**2.31**	**8.87**	**6.06**	**-0.40**	**-0.66**	**-0.14**
**12:01:01G**	**04:02:01G**	0	.	.	1.00	.	.	0	.	.
**13:01:01G**	**03:01:01G**	0.23	-0.16	0.63	9.57E-20	.	.	**-0.65**	**-0.90**	**-0.39**
**13:01:01G**	**05:02:01G**	**-0.61**	**-0.83**	**-0.39**	**2.11E-17**	**2.73E-18**	**1.64E-16**	**0.40**	**0.003**	**0.81**
**13:01:01G**	**05:03:01G**	0	.	.	1.00	.	.	0	.	.
**13:01:01G**	**06:02:01G**	0	.	.	**4.24E-17**	**4.75E-18**	**3.79E-16**	0.10	-0.19	0.41
**13:01:01G**	**06:03:01G**	-0.06	-0.22	0.08	1.19	0.68	2.08	0.10	-0.21	0.43
**13:02:01G**	**03:01:01G**	-0.09	-0.22	0.03	**4.93E-17**	**8.60E-18**	**2.83E-16**	0.41	-0.06	0.89
**13:02:01G**	**05:01:01G**	**-0.57**	**-0.72**	**-0.42**	**2.18E-14**	**2.69E-15**	**1.78E-13**	**2.14**	**1.76**	**2.52**
**13:02:01G**	**06:02:01G**	0	.	.	**1.21E-15**	**1.35E-16**	**1.08E-14**	**2.53**	**2.13**	**2.93**
**13:02:01G**	**06:04:01G**	0.02	-0.20	0.26	1.34	0.52	3.45	**2.71**	**2.13**	**3.29**
**13:02:01G**	**06:09:01G**	-0.31	-0.70	0.07	1.35	0.26	6.99	0.10	-0.63	0.83
**13:03:01G**	**03:01:01G**	**1.58**	**1.31**	**1.85**	2.04	0.51	8.10	-0.02	-0.66	0.61
**13:03:01G**	**03:02:01G**	0	.	.	1.00	.	.	0	.	.
**13:03:01G**	**04:02:01G**	**1.58**	**1.31**	**1.85**	**4.92E-18**	**6.19E-19**	**3.91E-17**	**-0.70**	**-0.99**	**-0.42**
**13:03:01G**	**05:01:01G**	0	.	.	1.00	.	.	**2.24**	**1.83**	**2.65**
**13:04**	**03:01:01G**	5.81	0.29 .	4.07	**2.27**	**1.12**	**4.61**	**8.39**	**0.05**	**9.17**
**13:04**	**04:02:01G**	1.16	3.27	9.45	**2.27**	**1.12**	**4.61**	1.94	-014	0.787
**13:05:01**	**03:01:01G**	**1.13**	**0.85**	**1.41**	1.84	0.22	1.51	**0.59**	**-1.13**	**-0.06**
**13:05:01**	**03:02:01G**	**1.13**	**0.85**	**1.41**	**9.26E-18**	**1.06E-18**	**8.10E-17**	**0.60**	**-1.13**	**-0.06**
**14:01:01G**	**05:03:01G**	**1.83E-16**[b]	**3.47E-17**	**9.67E-16**	0.53	0.038	7.32	-0.11	-0.47	0.24
**14:01:01G**	**06:03:01G**	0	.	.	0.06[a]	-0.13	0.26	0	.	.
**14:02:01G**	**03:01:01G**	**0.24**	**0.01**	**0.46**	1.49	0.70	3.16	0.03	-0.32	0.38
**14:02:01G**	**03:02:01G**	0.23	-0.15	0.63	1.24	0.72	2.13	0.36	0.08	0.64

**14:02:01G**	**04:02:01G**	0	.	.	4.27E-18	.	.	**2.27**	**1.76**	**2.77**
**14:02:01G**	**05:01:01G**	**1.55**	**1.27**	**1.82**	1.96	0.86	4.47	**0.56**	**0.12**	**1.00**
**14:02:01G**	**06:04:01G**	-0.13	-0.37	0.10	3.45E-19	.	.	0.23	-0.24	0.72
**14:02:01G**	**06:09:01G**	0	.	.	1.00	.	.	0	.	.
**14:04:01**	**03:01:01G**	0	.	.	1.00	.	.	0	.	.
**14:06:01**	**06:03:01G**	0	.	.	1.00	.	.	0	.	.
**15:01:01G**	**05:01:01G**	-0.04	-0.42	0.33	0.38	0.06	2.15	-0.30	-0.67	0.06
**15:01:01G**	**05:02:01G**	**-0.39**	**-0.62**	**-0.16**	**1.40E-15**	**1.80E-16**	**1.08E-14**	**2.63**	**2.24**	**3.02**
**15:01:01G**	**06:02:01G**	0.12	-0.08	0.32	0.78	0.30	2.00	-0.10	-0.41	0.19
**15:01:01G**	**06:03:01G**	0	.	.	**4.24E-17**	**4.75E-18**	**3.79E-16**	0.10	-0.19	0.41
**15:01:01G**	**06:04:01G**	0	.	.	1.00	.	.	0	.	.
**15:02:01G**	**06:01:01G**	-0.14	-0.43	0.14	1.17	0.23	5.83	**2.73**	**2.18**	**3.27**
**15:03:01G**	**06:02:01G**	0.10	-0.09	0.29	**3.24**	**1.17**	**8.91**	**0.35**	**0.10**	**0.61**
**16:01:01**	**03:01:01G**	**1.22**	**0.90**	**1.54**	**1.85**	**1.16**	**2.66**	**0.57**	**0.17**	**0.96**
**16:01:01**	**05:02:01G**	-0.26	-0.55	0.03	1.71	0.33	8.70	**0.57**	**0.14**	**0.99**
**16:01:01**	**06:03:01G**	**-0.61**	**-0.83**	**-0.39**	**2.11E-17**	**2.73E-18**	**1.64E-16**	**0.40**	**0.003**	**0.81**
**16:02:01G**	**03:01:01G**	0.16	-0.06	0.38	0.64	0.25	1.60	0.01	-0.42	0.46
**16:02:01G**	**03:02:01G**	-0.10	-0.34	0.13	0.48	0.10	2.27	0.01	-0.56	0.60
**16:02:01G**	**03:03:02G**	-0.02	-0.17	0.13	6.38E-20	.	.	**0.44**	**0.06**	**0.82**
**16:02:01G**	**04:02:01G**	**1.61**	**1.34**	**1.88**	**2.78E-14**	**3.58E-15**	**2.15E-13**	0	.	.
**16:02:01G**	**05:01:01G**	0	.	.	**5.10**	**1.98**	**1.31**	**2.36**	**1.93**	**2.78**
**16:02:01G**	**05:02:01G**	-0.18	-0.46	0.10	3.44	0.91	1.29	**-0.42**	**-0.68**	**-0.16**
**16:02:01G**	**05:03:01G**	0	.	.	1.00	.	.	0	.	.
**16:02:01G**	**06:03:01G**	**1.67**	**1.38**	**1.97**	9.57E-20	.	.	**-0.65**	**-0.90**	**-0.39**
**16:02:01G**	**06:04:01G**	0	.	.	1.00	.	.	0	.	.
**16:02:01G**	**06:09:01G**	0	.	.	**2.14E-14**	**2.42E-15**	**1.89E-13**	0.23	-0.24	0.72

Analysis was adjusted for origin, age, lifetime amount of sexual partners, abortions, coinfection and viral load (low: ≤9.99E+5, medium: 1.00E+6 to 9.99E+9, high: ≥1.00E+10).

95%CI: 95% confidence interval, HPV: human papillomavirus, Coeff: regression coefficient (hazard ratio for Cox proportional hazards regression model and time ratio for log-normal parametric model), *DRB1: DR beta 1, DQB1: DQ beta 1*

Values in bold indicate statistical significance based on the 95%CI, $p<0.05$.

[a] Cox proportional hazards model did not fulfil the assumption of proportionality; the log-normal parametric model was used in such cases

[b] The Cox proportional hazards model was used.

Supplementary Table S11. Multivariate models evaluating the association between *DRB1* and *DQB1* haplotypes and HR-HPV redetection for HPV-45 and -58

Haplotype		HPV-45[a]			HPV-58		
DRB1	***DQB1***	**Coeff.**	**95%CI**		**Coeff.**	**95%CI**	
**01:01:01G**	**03:01:01G**	**3.32**	**2.71**	**3.92**	**3.67E-15[b]**	**6.14E-16**	**2.19E-14**
**01:01:01G**	**03:02:01G**	-0.49	-0.99	0.01	1.29[b]	0.23	7.18
**01:01:01G**	**03:03:02G**	**2.40**	**1.89**	**2.92**	0	.	.
**01:01:01G**	**04:02:01G**	**1.02**	**0.32**	**1.72**	0	.	.
**01:01:01G**	**05:01:01G**	0.27	-0.26	0.82	0.40	-0.12	0.94
**01:02:01G**	**02:01:01G**	**-0.63**	**-1.03**	**-0.24**	**3.40E-17[b]**	**3.97E-18**	**2.91E-16**
**01:02:01G**	**03:01:01G**	**2.93**	**2.36**	**3.50**	**2.31**	**1.64**	**2.98**
**01:02:01G**	**03:02:01G**	0.58	-0.10	1.27	-0.54	-1.12	0.02
**01:02:01G**	**03:03:02G**	**1.08**	**0.48**	**1.68**	-0.27	-0.65	0.09
**01:02:01G**	**04:02:01G**	0	.	.	0	.	.
**01:02:01G**	**05:01:01G**	-0.14	-0.56	0.27	-0.15	-0.65	0.34
**01:03:01**	**02:01:01G**	0	.	.	0	.	.
**01:03:01**	**03:01:01G**	**2.71**	**2.16**	**3.27**	**3.48**	**2.91**	**4.06**
**01:03:01**	**03:02:01G**	**2.96**	**2.39**	**3.53**	**-0.85**	**-1.29**	**-0.40**
**01:03:01**	**04:02:01G**	**3.06**	**2.53**	**3.60**	0	.	.
**01:03:01**	**05:01:01G**	**3.30**	**2.81**	**3.79**	0.21[b]	0.01	2.86
**01:03:01**	**05:02:01G**	0	.	.	0	.	.
**03:01:01G**	**02:01:01G**	**-0.41**	**-0.79**	**-0.04**	0.23	-0.38	0.85
**03:01:01G**	**05:01:01G**	**-0.63**	**-1.03**	**-0.24**	**2.42**	**1.68**	**3.15**
**03:02:01**	**02:01:01G**	0	.	.	0	.	.
**03:02:01**	**04:02:01G**	**-0.40**	**-0.67**	**-0.13**	-0.38	-0.83	0.07
**03:02:02**	**02:01:01G**	0	.	.	**1.66**	**0.86**	**2.46**
**03:02:02**	**03:01:01G**	-0.28	-0.72	0.14	**1.80**	**1.04**	**2.56**
**04:01:01G**	**03:02:01G**	0	.	.	0	.	.
**04:02:01**	**02:01:01G**	**1.09**	**0.51**	**1.67**	**1.05**	**0.55**	**1.55**
**04:02:01**	**03:01:01G**	**2.08**	**2.37**	**3.41**	-0.27	-0.65	0.11
**04:02:01**	**03:02:01G**	0.37	-0.32	1.07	-0.04	-0.46	0.45
**04:02:01**	**05:01:01G**	0	.	.	0	.	.
**04:03:01G**	**02:01:01G**	-0.03	-0.48	0.41	**2.49**	**1.90**	**3.09**
**04:03:01G**	**03:01:01G**	0	.	.	**2.01**	**1.34**	**2.69**
**04:03:01G**	**03:02:01G**	0.08	-0.36	0.52	**2.74**	**2.12**	**3.36**
**04:04:01**	**02:01:01G**	**1.07**	**0.52**	**1.83**	**-0.93**	**-1.37**	**-0.49**
**04:04:01**	**03:01:01G**	**1.01**	**0.65**	**1.72**	0	.	.
**04:04:01**	**03:02:01G**	-0.29	-0.79	0.20	-0.23	-0.90	0.43
**04:04:01**	**05:01:01G**	**-0.70**	**-1.02**	**-0.38**	-0.64	-1.47	0.19
**04:05:01**	**02:01:01G**	**-1.05**	**-1.41**	**-0.69**	**0.65**	**0.07**	**1.23**
**04:05:01**	**03:01:01G**	0.88	-0.25	0.43	**2.59**	**2.01**	**3.18**
**04:05:01**	**03:02:01G**	-0.27	-0.94	0.40	-0.01	-0.58	0.56
**04:05:04**	**02:01:01G**	0	.	.	**2.93**	**2.25**	**3.61**

**04:05:04**	**03:01:01G**	**-0.64**	**-1.03**	**-0.25**	**0.70**	**0.16**	**1.25**
**04:05:04**	**03:02:01G**	**1.25**	**0.59**	**1.91**	**2.11**	**1.37**	**2.85**
**04:05:04**	**04:02:01G**	-0.58	-0.69	0.68	**2.92**	**2.24**	**3.59**
**04:05:04**	**05:01:01G**	**3.06**	**2.53**	**3.60**	0	.	.
**04:06:01G**	**04:02:01G**	0	.	.	0	.	.
**04:07:01G**	**02:01:01G**	0.37	-0.24	0.99	**3.30**	**2.65**	**3.95**
**04:07:01G**	**03:01:01G**	**-0.36**	**-0.63**	**-0.09**	0.37	-0.21	0.96
**04:07:01G**	**03:02:01G**	-0.04	-0.31	0.22	0.09	-0.24	0.42
**04:07:01G**	**03:02:03**	**-0.71**	**-1.04**	**-0.38**	**-1.00**	**-1.47**	**-0.53**
**04:07:01G**	**04:02:01G**	**-0.54**	**-0.87**	**-0.21**	-0.54	-1.20	0.11
**04:07:01G**	**05:01:01G**	0.11	-0.41	0.63	**-0.46**	**-0.84**	**-0.08**
**04:08:01**	**02:01:01G**	**2.27**	**1.77**	**2.76**	**3.47**	**2.97**	**3.96**
**04:08:01**	**03:01:01G**	**2.27**	**1.77**	**2.76**	**3.47**	**2.97**	**3.96**
**04:10:01G**	**03:01:01G**	0	.	.	0	.	.
**04:10:01G**	**04:02:01G**	0	.	.	0	.	.
**04:11:01**	**02:01:01G**	-0.21	-0.55	0.12	-0.47	-1.36	0.42
**04:11:01**	**03:02:01G**	**-0.40**	**-0.75**	**-0.06**	**-0.41**	**-0.77**	**-0.04**
**04:11:01**	**04:02:01G**	0	.	.	-0.21	-1.12	0.70
**07:01:01G**	**02:01:01G**	0.27	-0.19	0.74	0.04	-0.52	0.61
**07:01:01G**	**03:01:01G**	**2.61**	**2.12**	**3.09**	**3.49**	**3.00**	**3.98**
**07:01:01G**	**03:02:01G**	0.36	-0.34	1.06	**2.89**	**2.24**	**3.54**
**07:01:01G**	**03:03:02G**	**2.40**	**1.89**	**2.92**	-0.02	-0.46	0.41
**07:01:01G**	**04:02:01G**	**2.40**	**1.82**	**2.97**	**2.95**	**2.27**	**3.62**
**07:01:01G**	**05:01:01G**	**2.40**	**1.89**	**2.92**	0.01	-0.58	0.61
**07:11**	**03:02:01G**	-0.21	0.55	0.12	-4.71	-1.36	0.42
**07:11**	**04:02:01G**	0	.	.	-0.25	-0.98	0.48
**08:01:01G**	**03:01:01G**	**2.83**	**2.31**	**3.34**	**2.05**	**1.40**	**2.70**
**08:01:01G**	**04:02:01G**	**2.83**	**2.31**	**3.34**	**2.05**	**1.40**	**2.70**
**08:02:01G**	**03:01:01G**	-0.47	-1.23	0.27	4.47 [b]	0.63	3.13
**08:02:01G**	**04:02:01G**	**-0.51**	**-0.80**	**-0.23**	3.26[b]	0.92	1.15
**08:02:01G**	**05:01:01G**	**1.02**	**0.32**	**1.72**	0	.	.
**08:03:02**	**04:02:01G**	0	.	.	0	.	.
**08:04:01**	**03:01:01G**	0.10	-0.72	0.92	**2.85**	**2.27**	**3.43**
**08:04:01**	**03:02:01G**	**-0.63**	**-0.97**	**-0.29**	**2.77**	**2.09**	**3.46**
**08:04:01**	**04:02:01G**	0	.	.	0	.	.
**08:04:01**	**05:01:01G**	**2.93**	**2.36**	**3.50**	**2.32**	**1.74**	**2.90**
**08:06:06**	**02:01G**	0	.	.	0	.	.
**08:07**	**04:02:01G**	0	.	.	**2.51**	**0.89**	**3.13**
**09:01:02G**	**03:01:01G**	**2.50**	**1.99**	**3.00**	-0.46	-0.96	0.03
**09:01:02G**	**03:03:02G**	0.42	-0.01	0.87	0.13	-0.33	0.59
**10:01:01G**	**05:01:01G**	**-0.49**	**-0.82**	**-0.15**	-0.01	-0.75	0.71
**11:01:01G**	**03:01:01G**	-0.20	-0.63	0.21	-0.47	-1.39	0.44
**11:01:01G**	**03:02:01G**	-0.20	-0.63	0.21	-0.26	-1.65	1.11
**11:01:02**	**05:01:01G**	0	.	.	0	.	.
**11:02:01**	**03:01:01G**	0.73	-0.16	0.31	**2.65**	**1.72**	**3.58**
**11:02:01**	**03:02:01G**	0	.	.	0	.	.

**11:02:01**	**04:02:01G**	0	.	.	0	.	.
**11:02:01**	**05:01:01G**	0	.	.	**2.37**	**1.42**	**3.31**
**11:04:01G**	**02:01:01G**	**2.12**	**1.58**	**2.65**	**-0.68**	**-1.18**	**-0.19**
**11:04:01G**	**03:01:01G**	**0.53**	**0.22**	**0.84**	0.48[b]	0.10	2.37
**11:04:01G**	**03:02:01G**	**2.15**	**1.61**	**2.68**	**2.88**	**2.33**	**3.43**
**11:04:01G**	**04:02:01G**	**1.18**	**0.58**	**1.78**	**0.70**	**0.14**	**1.26**
**11:04:01G**	**05:01:01G**	**2.41**	**1.89**	**2.94**	**3.21**	**2.66**	**3.75**
**11:04:01G**	**06:03:01G**	**2.12**	**1.58**	**2.65**	**-0.68**	**-1.18**	**-0.19**
**11:14:01**	**04:02:01G**	**-0.87**	**-1.31**	**-0.43**	**-0.80**	**-1.34**	**-0.26**
**12:01:01G**	**03:02:01G**	**2.84**	**2.33**	**3.36**	**-0.54**	**-1.03**	**-0.06**
**12:01:01G**	**04:02:01G**	0	.	.	0	.	.
**13:01:01G**	**03:01:01G**	0.58	-0.31	1.47	**2.07**	**1.40**	**2.74**
**13:01:01G**	**05:02:01G**	**2.68**	**2.22**	**3.14**	**2.22**	**1.32**	**3.12**
**13:01:01G**	**05:03:01G**	0	.	.	0	.	.
**13:01:01G**	**06:02:01G**	-0.08	-0.43	0.41	**2.50**	**1.77**	**3.24**
**13:01:01G**	**06:03:01G**	0.21	-0.19	0.62	-0.32	-0.80	0.15
**13:02:01G**	**03:01:01G**	0.14	-0.29	0.59	-0.09	-0.46	0.27
**13:02:01G**	**05:01:01G**	**-0.47**	**-0.79**	**-0.14**	0	.	.
**13:02:01G**	**06:02:01G**	**2.87**	**2.34**	**3.40**	-0.06	-0.91	0.77
**13:02:01G**	**06:04:01G**	-0.12	-0.55	0.29	-0.24	-0.53	0.03
**13:02:01G**	**06:09:01G**	0.06	-0.55	0.67	0.08	-0.57	0.59
**13:03:01G**	**03:01:01G**	**2.37**	**1.75**	**3.00**	**2.41**	**1.84**	**2.99**
**13:03:01G**	**03:02:01G**	0	.	.	0	.	.
**13:03:01G**	**04:02:01G**	0	.	.	**2.05**	**1.40**	**2.70**
**13:03:01G**	**05:01:01G**	**2.37**	**1.75**	**3.00**	**2.41**	**1.83**	**2.99**
**13:04**	**03:01:01G**	**2.83**	**2.31**	**3.34**	**0.63**	**0.01**	**1.26**
**13:04**	**04:02:01G**	**2.83**	**2.31**	**3.34**	**0.63**	**0.01**	**1.26**
**13:05:01**	**03:01:01G**	**2.50**	**1.99**	**3.00**	**0.81**	**0.24**	**1.39**
**13:05:01**	**03:02:01G**	0	.	.	**-1.23**	**-2.21**	**-0.25**
**14:01:01G**	**05:03:01G**	**-0.54**	**-1.02**	**-0.06**	-0.56	-1.28	.1.51
**14:01:01G**	**06:03:01G**	0	.	.	0	.	.
**14:02:01G**	**03:01:01G**	0.26	-0.03	0.55	0.30	-0.19	0.79
**14:02:01G**	**03:02:01G**	-0.22	-0.60	0.16	0.93[b]	0.28	3.05
**14:02:01G**	**04:02:01G**	**2.30**	**1.68**	**2.92**	**2.14**	**1.54**	**2.74**
**14:02:01G**	**05:01:01G**	0	.	.	-0.38	-1.29	0.52
**14:02:01G**	**06:04:01G**	-0.04	-0.43	0.35	**1.96**	**1.37**	**2.55**
**14:02:01G**	**06:09:01G**	0	.	.	0	.	.
**14:04:01**	**03:01:01G**	0	.	.	0	.	.
**14:06:01**	**06:03:01G**	0	.	.	0	.	.
**15:01:01G**	**05:01:01G**	-0.27	-0.87	0.33	-0.21	-0.72	0.29
**15:01:01G**	**05:02:01G**	**-0.40**	**-0.67**	**-0.13**	-0.38	-0.83	0.07
**15:01:01G**	**06:02:01G**	0.17	-0.16	0.52	0.05	-0.42	0.43
**15:01:01G**	**06:03:01G**	-0.008	-0.43	0.41	**2.50**	**1.77**	**3.24**
**15:01:01G**	**06:04:01G**	0	.	.	0	.	.
**15:02:01G**	**06:01:01G**	0.34	-0.63	1.31	**2.75**	**2.14**	**3.37**
**15:03:01G**	**06:02:01G**	**2.16**	**1.51**	**2.80**	**3.25**	**2.75**	**3.75**

**16:01:01**	**03:01:01G**	**2.04**	**1.88**	**2.92**	**0.97**	**0.23**	**1.71**
**16:01:01**	**05:02:01G**	**2.07**	**2.30**	**3.17**	**3.10**	**2.45**	**3.75**
**16:01:01**	**06:03:01G**	**2.06**	**2.22**	**3.14**	**2.22**	**1.32**	**3.12**
**16:02:01G**	**03:01:01G**	-0.06	-0.54	0.41	**0.66**	**0.08**	**1.23**
**16:02:01G**	**03:02:01G**	-0.34	-0.84	0.15	0.58	-0.03	1.20
**16:02:01G**	**03:03:02G**	0	.	.	-0.14	-0.49	0.21
**16:02:01G**	**04:02:01G**	**-0.64**	**-1.03**	**-0.25**	**0.70**	**0.16**	**1.25**
**16:02:01G**	**05:01:01G**	**3.32**	**2.71**	**3.92**	**1.90**	**1.16**	**2.64**
**16:02:01G**	**05:02:01G**	**2.40**	**1.88**	**2.92**	**1.90**	**1.16**	**2.64**
**16:02:01G**	**05:03:01G**	0	.	.	-0.1[b]	-0.52	0.15
**16:02:01G**	**06:03:01G**	**3.50**	**3.03**	**3.97**	-0.59	-1.56	0.36
**16:02:01G**	**06:04:01G**	0	.	.	0	.	.
**16:02:01G**	**06:09:01G**	**1.05**	**0.37**	**1.74**	**1.98**	**1.17**	**2.80**

Analysis was adjusted for origin, age, lifetime amount of sexual partners, abortions, coinfection and viral load (low: ≤9.99E+5, medium: 1.00E+6 to 9.99E+9, high: ≥1.00E+10).

95%CI: 95% confidence interval, HPV: human papillomavirus, Coeff: regression coefficient (hazard ratio for Cox proportional hazards regression model and time ratio for log-normal parametric model), *DRB1: DR beta 1, DQB1: DQ beta 1*

Values in bold indicate statistical significance based on the 95%CI, $p<0.05$

[a] The Cox proportional hazards model did not fulfil the assumption of proportionality; the log-normal parametric model was used in such cases

[b] The Cox proportional hazards model was used.

Supplementary Table S12. DRB1 and DQB1 alleles associated with infection clearance/persistence for each of the 6 HR-HPV types

Allele	Coeff.	95%CI		HR-HPV type	Effect regarding clearance	*p*	*p*[a]	Previous studies	References
		Lower	Upper						
DRB1									
**01:02:01G**	2.50	1.12	5.60	18	Greater probability	0.025	1.175	*01 protective factor for CC<br><br>*01:02 lower risk of SIL and CC	[1, 2]
**01:03:01**	-0.75[b]	-1.31	-0.19	31	Earlier occurrence	0.000	**0.000**		
**01:03:01**	2.92	1.42	5.98	45	Greater probability	0.003	0.141		
**03:01:01G**	3.49	1.32	9.26	33	Greater probability	0.012	0.564	*03 lower risk of CC and HPV-16<br><br>*03:01 increased risk of CC (case-control studies) and lower risk of HR-HPV and persistence (cohort study)	[1-4]
**03:02:01**	7.16E-18	8.15E-19	6.30E-17	16	Lower probability	0.000	**0.000**		
**03:02:01**	-1.55[b]	-2.01	-1.09	31	Earlier occurrence	0.000	**0.000**		
**03:02:01**	8.59	2.76	26.73	33	Greater probability	0.000	**0.000**		
**03:02:01**	1.65E-16	2.13E-17	1.28E-15	45	Lower probability	0.000	**0.000**		
**03:02:01**	8.69E-17	1.02E-17	7.40E-16	58	Lower probability	0.000	**0.000**		
**03:02:02**	2.60E-10	2.52E-11	2.68E-09	33	Lower probability	0.000	**0.000**		
**04:02:01**	8.00E-16	2.99E-16	2.14E-15	31	Lower probability	0.000	**0.000**	*04 increased risk of LIE-AG, CC, and HPV, especially in the Caucasian and Mexican population, less frequency in redetection<br><br>*04:05 increased risk of CC in Caucasian and Hispanic population and higher risk of HPV in the Brazilian population	[2, 5-8]
**04:05:01**	3.16	1.59	6.28	16	Greater probability	0.001	**0.047**		
**04:07:01G**	1.72	1.03	2.85	58	Greater probability	0.037	1.739		
**04:08:01**	3.64E-16	4.11E-17	3.23E-15	16	Lower probability	0.000	**0.000**		
**04:08:01**	4.22	1.40	1.27	18	Greater probability	0.011	0.517		
**04:10:01G**	1.11E-17	1.11E-18	1.11E-16	33	Lower probability	0.000	**0.000**		
**04:10:01G**	4.02E-14	4.79E-15	3.37E-13	45	Lower probability	0.000	**0.000**		
**04:11:01**	4.43E-15	1.37E-15	1.43E-14	31	Lower probability	0.000	**0.000**		
**04:11:01**	5.48E-16	1.56E-16	1.92E-15	45	Lower probability	0.000	**0.000**		
**04:11:01**	1.71E-18	5.97E-19	4.92E-18	58	Lower probability	0.000	**0.000**		
**07:11**	3.47E-17	7.58E-18	1.59E-16	16	Lower probability	0.000	**0.000**	*07 lower risk of persistence	[5]
**07:11**	15.33	5.17	45.46	18	Greater probability	0.000	**0.000**		
**07:11**	5.15E-19	6.56E-20	4.05E-18	45	Lower probability	0.000	**0.000**		
**07:11**	3.52[b]	2.93	4.11	58	Later occurrence	0.000	**0.000**		
**08:01:01G**	1.15E-14	3.17E-15	4.15E-14	31	Lower probability	0.000	**0.000**	*08 higher risk of SIL and CC	

**08:04:01**	1.33E-15	3.53E-16	4.99E-15	31	Lower probability	0.000	**0.000**	*08:01 increased risk of CC in Asian population<br>*08:07 increased risk of HPV-16 and viral persistence	[1, 2, 9]
**08:04:01**	-0.67[b]	-0.97	-0.36	33	Earlier occurrence	0.000	**0.000**		
**08:04:01**	3.80	1.47	9.86	58	Greater probability	0.006	0.282		
**08:07**	-1.47[b]	-2.10	-0.84	18	Earlier occurrence	0.000	**0.000**		
**08:07**	2.96	1.47	5.96	31	Greater probability	0.020	0.940		
**08:07**	6.79E-17	7.26E-18	6.35E-16	33	Lower probability	0.000	**0.000**		
**08:07**	6.78E-15	7.83E-16	5.87E-14	45	Lower probability	0.000	**0.000**		
**08:07**	-0.28[b]	-0.52	-0.05	58	Earlier occurrence	0.017	0.799		
**09:01:02G**	-0.70[b]	-1.16	-0.24	45	Earlier occurrence	0.003	0.141	*09:01 lower risk of HPV-18 persistence in Taiwanese population, lower risk of CC in Caucasian population.<br>*09:01:02 increased risk of CC and HPV-16 infection in Asian population.	[6] [8] [10]
**10:01:01G**	3.79	1.50	9.55	33	Greater probability	0.005	0.235	*10 increased risk of HPV infection and reinfection, lower risk of SIL	[2, 5]
**11:02:01**	5.15E-15	1.31E-15	2.02E-14	31	Lower probability	0.000	**0.000**	*11 increased risk of CC and SIL plus HPV	[2]
**11:02:01**	1.97	1.08	3.59	45	Greater probability	0.027	1.269		
**11:14:01**	3.43	1.21	9.74	18	Greater probability	0.021	0.987		
**11:14:01**	1.58E-16	1.89E-17	1.33E-15	31	Lower probability	0.000	**0.000**		
**11:14:01**	8.05E-16	1.03E-16	6.30E-15	45	Lower probability	0.000	**0.000**		
**11:14:01**	4.08[b]	3.35	4.81	58	Later occurrence	0.000	**0.000**		
**12:01:01G**	4.80	2.67	8.64	16	Greater probability	0.000	**0.000**	*12 lower CC risk in Chinese population	[2, 4]
**12:01:01G**	2.79E-15	2.74E-16	2.84E-14	18	Lower probability	0.000	**0.000**		
**12:01:01G**	8.24E-17	1.68E-17	4.03E-16	31	Lower probability	0.000	**0.000**		
**12:01:01G**	3.58	1.34	9.58	33	Greater probability	0.011	0.517		
**12:01:01G**	12.65	4.93	32.45	45	Greater probability	0.000	**0.000**		
**12:01:01G**	1.65E-14	3.59E-15	7.55E-14	58	Lower probability	0.000	**0.000**		
**13:01:01G**	1.93	1.07	3.50	45	Greater probability	0.029	1.363	*13 lower risk of CC, HSIL plus HPV-16 in Caucasians, higher risk in Chinese population<br>*13:01 lower risk of CC and SIL plus with HPV	[2, 11, 12]
**13:03:01G**	2.29E-16	4.88E-17	1.08E-15	18	Lower probability	0.000	**0.000**		
**13:03:01G**	10.94	2.66	45.00	33	Greater probability	0.001	**0.047**		
**13:03:01G**	22.15	8.34	58.85	45	Greater probability	0.000	**0.000**		
**13:03:01G**	-0.57[b]	-1.13	-0.01	58	Earlier occurrence	0.048	2.256		
**13:04**	5.04E-19	7.32E-20	3.48E-18	18	Lower probability	0.000	**0.000**		

**13:04**	1.19E-14	2.24E-15	6.27E-14	31	Lower probability	0.000	**0.000**		
**13:04**	2.67	1.22	5.81	45	Greater probability	0.013	0.611		
**13:04**	2.75[b]	2.11	3.39	58	Later occurrence	0.000	**0.000**		
**13:05:01**	3.21	1.68	6.12	16	Greater probability	0.000	**0.000**		
**13:05:01**	6.84E-16	1.90E-16	2.46E-15	18	Lower probability	0.000	**0.000**		
**13:05:01**	4.20[b]	3.08	5.31	31	Later occurrence	0.000	**0.000**		
**13:05:01**	1.09E-17	1.65E-18	7.24E-17	58	Lower probability	0.000	**0.000**		
**15:02:01G**	3.74E-16	7.98E-17	1.75E-15	18	Lower probability	0.000	**0.000**	*15 increased risk of SIL and CC plus HPV and HPV-16<br>*15:02 higher risk of CC in Chinese population<br>*15:03:01 increased risk of CC, SIL and HPV	[2, 4, 7, 8]
**15:03:01G**	20.24	8.61	47.55	16	Greater probability	0.000	**0.000**		
**15:03:01G**	1.89E-17	3.79E-18	9.44E-17	18	Lower probability	0.000	**0.000**		
DQB1									
**03:02:03**	2.25	1.23	4.11	16	Greater probability	0.008	0.112	*03 higher risk of CC in different populations (Caucasian, Oriental and Latin American), higher risk of SIL plus HR-HPV / HPV-16<br>*03:02 increased risk of SIL, CC and HPV-18 persistence	[2, 6, 11, 13]
**03:02:03**	1.62E-15	2.00E-16	1.30E-14	18	Lower probability	0.000	**0.000**		
**03:02:03**	-1.22[b]	-1.67	-0.78	31	Earlier occurrence	0.000	**0.000**		
**03:02:03**	7.39E-16	3.63E-17	1.50E-14	45	Lower probability	0.000	**0.000**		
**03:02:03**	1.51E-15	1.76E-16	1.29E-14	58	Lower probability	0.000	**0.000**		
**04:02:01G**	-0.65[b]	-1.16	-1.16	18	Earlier occurrence	0.013	0.182	*04 increased risk of SIL[2][2][2]	
**05:02:01G**	8.59	2.76	26.73	33	Greater probability	0.000	**0.000**	*05 lower risk of CC and HPV-16 in Caucasian, higher risk of CC in China<br>*05:02 increased risk of HPV and persistence	[1, 2, 7, 11]
**06:01:01G**	3.74E-16	7.98E-17	1.75E-15	18	Lower probability	0.000	**0.000**	*06 lower risk of SIL and CC plus HPV, higher risk in Asian population<br>*06:01 increased risk of persistence, higher frequency of HPV-18 and -31 persistent infections<br>*06:03 lower risk of HSIL and CC	[1-3, 12]
**06:03:01G**	1.82	1.08	3.07	45	Greater probability	0.025	0.350		

95%CI: 95% confidence interval, Coeff: regression coefficient (hazard ratio for Cox proportional hazards regression model and time ratio for log-normal parametric model), *DRB1: DR beta 1, DQB1: DQ beta 1,* HR-HPV: high-risk human papillomavirus, SIL: squamous intraepithelial lesion, HSIL: high-grade squamous intraepithelial lesion, CC: cervical cancer.

[a] *p*: *p* value was corrected using the Bonferroni method

[b] The Cox proportional hazards model did not fulfil the assumption of proportionality; the log-normal parametric model was used in such cases

Supplementary Table S13. DRB1 and DQB1 alleles associated with HR-HPV redetection

Allele	Coeff.	95%CI		HR-HPV	Effect on redetection	*p*	*p*[a]
		Lower	Upper	type			
DRB1							
**01:01:01G**	2.88	1.34	6.17	18	Greater probability	0.007	0.329
**01:02:01G**	-0.37[b]	-0.68	-0.06	31	Earlier occurrence	0.021	0.987
**01:03:01**	2.79	1.56	5.02	16	Greater probability	0.001	**0.047**
**01:03:01**	4.87E-15	5.84E-16	4.06E-14	18	Lower probability	0.000	**0.000**
**01:03:01**	3.31[b]	2.82	3.80	45	Later occurrence	0.000	**0.000**
**03:01:01G**	-0.42[b]	-0.79	-0.05	45	Earlier occurrence	0.028	1.316
**03:02:01**	7.21E-16	8.94E-17	5.82E-15	18	Lower probability	0.000	**0.000**
**03:02:01**	2.63[b]	2.24	3.02	31	Later occurrence	0.000	**0.000**
**03:02:01**	-0.41[b]	-0.68	-0.14	45	Earlier occurrence	0.003	0.141
**03:02:02**	1.92[b]	1.55	2.30	31	Later occurrence	0.000	**0.000**
**03:02:02**	1.81[b]	1.05	2.57	58	Later occurrence	0.000	**0.000**
**04:02:01**	-0.45[b]	-0.66	-0.23	31	Earlier occurrence	0.000	**0.000**
**04:03:01G**	3.28	1.15	9.33	18	Greater probability	0.026	1.222
**04:03:01G**	2.74[b]	2.12	3.36	58	Later occurrence	0.000	**0.000**
**04:05:04**	2.92[b]	2.24	3.60	58	Later occurrence	0.000	**0.000**
**04:08:01**	32.15	8.08	127.87	16	Greater probability	0.000	**0.000**
**04:08:01**	2.13[b]	1.73	2.52	31	Later occurrence	0.000	**0.000**
**04:08:01**	2.27[b]	1.78	2.77	45	Later occurrence	0.000	**0.000**
**04:08:01**	3.47[b]	2.98	3.96	58	Later occurrence	0.000	**0.000**
**04:11:01**	-0.67[b]	-0.97	-0.38	31	Earlier occurrence	0.000	**0.000**
**04:11:01**	-0.41[b]	-0.75	-0.07	45	Earlier occurrence	0.019	0.893
**04:11:01**	-0.41[b]	-0.77	-0.05	58	Earlier occurrence	0.026	1.222
**08:01:01G**	-0.71[b]	-0.99	-0.42	31	Earlier occurrence	0.000	**0.000**
**08:01:01G**	2.83[b]	2.31	3.35	45	Later occurrence	0.000	**0.000**
**08:01:01G**	2.05[b]	1.40	2.70	58	Later occurrence	0.000	**0.000**
**08:02:01G**	-0.52[b]	-0.80	-0.23	45	Earlier occurrence	0.000	**0.000**
**08:04:01**	-0.62[b]	-0.97	-0.27	31	Earlier occurrence	0.001	**0.047**
**08:04:01**	2.85[b]	2.28	3.43	58	Later occurrence	0.000	**0.000**
**08:07**	0.45[b]	0.18	0.71	16	Earlier occurrence	0.001	**0.047**
**08:07**	4.56E-15	5.50E-16	3.78E-14	18	Lower probability	0.000	**0.000**
**08:07**	2.52[b]	1.90	3.14	58	Later occurrence	0.000	**0.000**
**09:01:02G**	0.52[b]	0.12	0.92	45	Earlier occurrence	0.010	0.470

**10:01:01G**	0.42	0.20	0.86	18	Lower probability	0.018	0.846
**10:01:01G**	-0.49[b]	-0.83	-0.16	45	Earlier occurrence	0.004	0.188
**11:01:01G**	2.82	1.15	6.87	16	Greater probability	0.023	1.081
**11:01:01G**	2.73[b]	2.34	3.13	31	Later occurrence	0.000	**0.000**
**11:02:01**	0.75[b]	0.29	1.21	31	Earlier occurrence	0.001	**0.047**
**11:02:01**	2.65[b]	1.72	3.58	58	Later occurrence	0.000	**0.000**
**11:04:01G**	0.17	0.06	0.45	18	Lower probability	0.000	**0.000**
**11:04:01G**	0.55[b]	0.24	0.86	45	Earlier occurrence	0.001	**0.047**
**11:14:01**	-0.71[b]	-0.99	-0.42	31	Earlier occurrence	0.000	**0.000**
**11:14:01**	-0.88[b]	-1.32	-0.43	45	Earlier occurrence	0.000	**0.000**
**11:14:01**	-0.81[b]	-1.35	-0.26	58	Earlier occurrence	0.004	0.188
**12:01:01G**	2.58	1.30	5.13	18	Greater probability	0.007	0.329
**12:01:01G**	-0.41[b]	-0.67	-0.14	31	Earlier occurrence	0.002	0.094
**12:01:01G**	2.85[b]	2.33	3.36	45	Later occurrence	0.000	**0.000**
**12:01:01G**	-0.55[b]	-1.03	-0.06	58	Earlier occurrence	0.027	1.269
**13:01:01G**	2.00	1.18	3.41	18	Greater probability	0.010	0.470
**13:03:01G**	3.55	1.09	11.59	18	Greater probability	0.036	1.692
**13:03:01G**	2.38[b]	1.76	3.00	45	Later occurrence	0.000	**0.000**
**13:03:01G**	2.42[b]	1.84	2.99	58	Later occurrence	0.000	**0.000**
**13:04**	-0.69[b]	-0.96	-0.41	18	Earlier occurrence	0.000	**0.000**
**13:04**	0.48[b]	0.05	0.92	31	Earlier occurrence	0.028	1.316
**13:04**	2.83[b]	2.31	3.35	45	Later occurrence	0.000	**0.000**
**13:04**	0.63[b]	0.01	1.26	58	Earlier occurrence	0.046	2.162
**13:05:01**	2.58	1.30	5.13	18	Greater probability	0.007	0.329
**13:05:01**	-0.60[b]	-1.13	-0.07	31	Earlier occurrence	0.028	1.316
**13:05:01**	2.50[b]	2.00	3.00	45	Later occurrence	0.000	**0.000**
**13:05:01**	0.82[b]	0.25	1.39	58	Earlier occurrence	0.005	0.235
**14:01:01G**	-0.55[b]	-1.02	-0.07	45	Earlier occurrence	0.025	1.175
**14:02:01G**	2.12	1.04	4.34	18	Greater probability	0.040	1.880
**15:02:01G**	3.66	1.56	8.60	18	Greater probability	0.003	0.141
**15:02:01G**	2.73[b]	2.19	3.28	31	Later occurrence	0.000	**0.000**
**15:02:01G**	2.76[b]	2.14	3.38	58	Later occurrence	0.000	**0.000**
**15:03:01G**	0.39[b]	0.12	0.67	16	Earlier occurrence	0.004	0.188
**15:03:01G**	-0.74[b]	-1.13	-0.35	18	Earlier occurrence	0.000	**0.000**
**15:03:01G**	0.36[b]	0.10	0.62	31	Earlier occurrence	0.006	0.282
**15:03:01G**	2.16[b]	1.51	2.81	45	Later occurrence	0.000	**0.000**
**15:03:01G**	3.26[b]	2.76	3.76	58	Later occurrence	0.000	**0.000**

**16:01:01**	4.42	1.94	10.10	18	Greater probability	0.000	**0.000**
**16:01:01**	0.57[b]	0.15	1.00	31	Earlier occurrence	0.008	0.376
**16:01:01**	2.74[b]	2.31	3.18	45	Later occurrence	0.000	**0.000**
**16:01:01**	3.10[b]	2.46	3.75	58	Later occurrence	0.000	**0.000**
**16:02:01G**	0.48[b]	0.04	0.92	58	Earlier occurrence	0.032	1.504
DQB1							
**03:02:03**	2.87	1.39	5.89	16	Greater probability	0.004	0.056
**03:02:03**	-0.71[b]	-1.05	-0.39	45	Earlier occurrence	0.000	**0.000**
**03:02:03**	-1.01[b]	-1.47	-0.54	58	Earlier occurrence	0.000	**0.000**
**03:03:02G**	0.52[b]	0.09	0.94	45	Earlier occurrence	0.017	0.238
**04:02:01G**	0.41	0.17	0.98	18	Lower probability	0.045	0.630
**04:02:01G**	-0.36[b]	-0.66	-0.06	45	Earlier occurrence	0.020	0.280
**05:02:01G**	-0.39[b]	-0.72	-0.06	16	Earlier occurrence	0.020	0.280
**05:02:01G**	2.35	1.06	5.19	18	Greater probability	0.035	0.490
**05:03:01G**	-0.55[b]	-1.02	-0.07	45	Earlier occurrence	0.025	0.350
**06:01:01G**	3.66	1.56	8.60	18	Greater probability	0.003	**0.042**
**06:01:01G**	2.73[b]	2.19	3.28	31	Later occurrence	0.000	**0.000**
**06:01:01G**	2.76[b]	2.14	3.38	58	Later occurrence	0.000	**0.000**
**06:03:01G**	2.00	1.18	3.41	18	Greater probability	0.010	0.140
**06:04:01G**	2.71[b]	2.13	3.29	31	Later occurrence	0.000	**0.000**

95%CI: 95% confidence interval, Coeff: regression coefficient (hazard ratio for Cox proportional hazards regression model and time ratio for log-normal parametric model), *DRB1: DR beta 1, DQB1: DQ beta 1,* HR-HPV: high-risk human papillomavirus

[a] *p*: *p* value was corrected using the Bonferroni method.

[b] The Cox proportional hazards model did not fulfil the assumption of proportionality; the log-normal parametric model was used in such cases

Supplementary Table S14. DRB1 and DQB1 haplotypes associated with infection clearance/persistence for each of the 6 HR-HPV types

Haplotype		Coeff.	95%CI		HR-HPV type	Effect on clearance	Previous studies	Ref
DRB1	*DQB1*		Lower	Upper				
**01:01:01G**	**03:01:01G**	2.00E-16	3.72E-17	1.08E-15	18	Lower probability		
**01:01:01G**	**03:01:01G**	2.17E-16	2.07E-17	2.27E-15	33	Lower probability		
**01:01:01G**	**03:02:01G**	4.30E-16	1.01E-16	1.83E-16	16	Lower probability		
**01:01:01G**	**03:03:02G**	17.09	4.51	64.70	33	Greater probability		
**01:01:01G**	**03:03:02G**	36.89	9.94	136.91	45	Greater probability		
**01:01:01G**	**03:03:02G**	1.43E-17	1.80E-18	1.13E-16	58	Lower probability		
**01:01:01G**	**04:02:01G**	2.32E-15	2.64E-16	2.05E-14	18	Lower probability		
**01:01:01G**	**04:02:01G**	7.52E-14	9.37E-15	6.04E-13	31	Lower probability		
**01:01:01G**	**04:02:01G**	5.44E-17	5.31E-18	5.57E-16	33	Lower probability		
**01:01:01G**	**04:02:01G**	2.01E-15	2.33E-16	1.73E-14	45	Lower probability		
**01:01:01G**	**04:02:01G**	1.60E-13	2.03E-14	1.27E-12	58	Lower probability		
**01:02:01G**	**02:01:01G**	2.43E-16	4.56E-16	1.29E-16	16	Lower probability		
**01:02:01G**	**02:01:01G**	1.63E-15	1.58E-16	1.67E-14	18	Lower probability		
**01:02:01G**	**02:01:01G**	4.05E-16	8.02E-17	2.05E-15	31	Lower probability		
**01:02:01G**	**02:01:01G**	3.66*	3.05	4.27	45	Later occurrence		
**01:02:01G**	**03:01:01G**	5.53	2.75	11.13	45	Greater probability		
**01:02:01G**	**03:02:01G**	-0.77*	-1.12	-0.42	45	Earlier occurrence		
**01:02:01G**	**03:03:02G**	3.11	1.72	5.63	16	Greater probability		
**01:02:01G**	**03:03:02G**	2.20E-15	2.51E-16	1.93E-14	18	Lower probability		
**01:02:01G**	**03:03:02G**	1.64E-15	2.10E-16	1.28E-14	31	Lower probability		
**01:02:01G**	**03:03:02G**	6.14E-17	7.55E-18	4.99E-16	45	Lower probability		
**01:02:01G**	**03:03:02G**	9.02E-19	1.15E-19	7.10E-18	58	Lower probability		
**01:03:01**	**02:01:01G**	2.72E-16	2.84E-17	2.61E-15	18	Lower probability		
**01:03:01**	**02:01:01G**	6.31	2.84	1.40	31	Greater probability		
**01:03:01**	**03:01:01G**	1.21E-17	1.50E-18	9.80E-17	16	Lower probability		
**01:03:01**	**03:02:01G**	6.63E-17	5.97E-18	7.36E-16	16	Lower probability		

**01:03:01**	**03:02:01G**	3.64	1.79	7.40	45	Greater probability		
**01:03:01**	**03:02:01G**	3.59*	3.04	4.13	58	Later occurrence		
**01:03:01**	**04:02:01G**	3.48E-14	4.04E-15	3.00E-13	31	Lower probability		
**01:03:01**	**04:02:01G**	5.97E-19	5.74E-20	6.20E-18	58	Lower probability		
**01:03:01**	**05:01:01G**	7.48E-16	9.57E-17	5.84E-15	16	Lower probability	*DRB1**01-*DQB1**05:01 lower risk of persistence	[1]
**03:01:01G**	**05:01:01G**	1.06E-14	1.18E-15	9.49E-14	31	Lower probability		
**03:01:01G**	**05:01:01G**	1.94E-14	1.86E-15	2.02E-13	45	Lower probability		
**03:02:01**	**04:02:01G**	7.86E-15	8.94E-16	6.90E-14	16	Lower probability		
**03:02:01**	**04:02:01G**	-1.54*	-2.01	-1.08	31	Earlier occurrence		
**03:02:01**	**04:02:01G**	8.58	2.75	2.67	33	Greater probability		
**03:02:01**	**04:02:01G**	3.51E-14	4.12E-15	2.98E-13	58	Lower probability		
**03:02:02**	**02:01:01G**	38.11	9.46	153.43	18	Greater probability		
**04:02:01**	**02:01:01G**	2.24E-14	2.72E-15	1.84E-13	31	Lower probability		
**04:02:01**	**02:01:01G**	2.65	2.16	3.15	33	Greater probability		
**04:02:01**	**02:01:01G**	1.22E-14	1.53e-15	9.82E-14	45	Lower probability		
**04:02:01**	**02:01:01G**	2.89E-18	3.68E-19	2.27E-17	58	Lower probability		
**04:02:01**	**03:01:01G**	1.11E-16	1.16E-17	1.05E-15	18	Lower probability		
**04:02:01**	**03:01:01G**	2.65E-16	5.63E-17	1.25E-15	31	Lower probability		
**04:02:01**	**03:02:01G**	8.00E-16	2.99E-16	2.14E-15	31	Lower probability		
**04:03:01G**	**02:01:01G**	4.53	2.13	9.62	31	Greater probability		
**04:03:01G**	**03:01:01G**	1.20E-14	1.31E-15	1.11E-13	18	Lower probability		
**04:03:01G**	**03:01:01G**	1.23E-15	1.22E-16	1.23E-14	31	Lower probability		
**04:03:01G**	**03:01:01G**	7.53	2.97	1.91	58	Greater probability		
**04:03:01G**	**03:02:01G**	5.43E-15	8.14E-16	3.62E-14	18	Lower probability	*DRB1**04:03-*DQB1**03:02 increased risk of HPV-18 persistence, HSIL and CC	[2, 6]
**04:04:01**	**02:01:01G**	4.76E-16	5.87E-17	3.86E-15	18	Lower probability		
**04:04:01**	**02:01:01G**	1.79E-16	1.99E-17	1.61E-15	31	Lower probability		
**04:04:01**	**02:01:01G**	2.01E-14	2.49E-15	1.63E-13	45	Lower probability		
**04:04:01**	**02:01:01G**	3.74*	3.19	4.28	58	Later occurrence		
**04:04:01**	**03:01:01G**	4.24	2.39	7.55	16	Greater probability		
**04:04:01**	**03:01:01G**	5.37E-18	1.18E-18	2.45E-17	18	Lower probability		

**04:04:01**	**03:01:01G**	4.17E-15	1.17E-15	1.49E-14	45	Lower probability		
**04:04:01**	**05:01:01G**	2.01E-18	2.26E-19	1.79E-17	16	Lower probability		
**04:04:01**	**05:01:01G**	1.73E-16	2.10E-17	1.42E-15	31	Lower probability		
**04:04:01**	**05:01:01G**	7.77E-17	9.90E-18	6.10E-16	45	Lower probability		
**04:04:01**	**05:01:01G**	2.05E-16	1.18E-17	3.57E-15	58	Lower probability		
**04:05:01**	**02:01:01G**	134.73	10.84	1673.64	33	Greater probability		
**04:05:01**	**02:01:01G**	1.25E-16	2.48E-17	6.27E-16	45	Lower probability		
**04:05:01**	**02:01:01G**	3.79*	3.23	4.36	58	Later occurrence		
**04:05:01**	**03:01:01G**	4.92	2.49	9.71	16	Greater probability		
**04:05:01**	**03:01:01G**	2.90E-15	5.71E-16	1.48E-14	31	Lower probability		
**04:05:01**	**03:01:01G**	5.37	2.17	13.28	45	Greater probability		
**04:05:04**	**02:01:01G**	8.26E-19	1.47E-19	4.63E-18	16	Lower probability		
**04:05:04**	**02:01:01G**	1.81E-17	2.20E-18	1.48E-16	45	Lower probability		
**04:05:04**	**03:01:01G**	3.51	1.74	7.09	16	Greater probability		
**04:05:04**	**03:01:01G**	4.31E-17	4.63E-18	4.01E-16	31	Lower probability		
**04:05:04**	**03:01:01G**	1.02E-13	6.26E-15	1.66E-12	45	Lower probability		
**04:05:04**	**03:01:01G**	3.22E-18	3.96E-19	2.61E-17	58	Lower probability		
**04:05:04**	**03:02:01G**	1.22E-14	1.43E-15	1.05E-13	16	Lower probability		
**04:05:04**	**03:02:01G**	2.98E-17	3.68E-18	2.41E-16	18	Lower probability	*DRB1**04:05-*DQB1**03:02 increased risk of HPV-18 persistence	[6]
**04:05:04**	**03:02:01G**	3.89E-16	4.88E-17	3.11E-15	31	Lower probability		
**04:05:04**	**03:02:01G**	5.68E-17	6.99E-18	4.61E-16	45	Lower probability		
**04:05:04**	**05:01:01G**	3.48E-14	4.04E15	3.00E-13	31	Lower probability		
**04:05:04**	**05:01:01G**	5.97E-19	5.74E-20	6.20E-18	58	Lower probability		
**04:07:01G**	**03:02:03**	1.19E-14	1.48E-15	9.63E-14	18	Lower probability		
**04:07:01G**	**03:02:03**	-1.22*	-1.66	-0.77	31	Earlier occurrence		
**04:07:01G**	**03:02:03**	1.48E-14	7.29E-16	3.02E-13	45	Lower probability		
**04:07:01G**	**03:02:03**	8.24E-14	9.61E-15	7.06E-13	58	Lower probability		
**04:07:01G**	**04:02:01G**	2.49E-16	4.69E-17	1.33E-15	45	Lower probability		
**04:08:01**	**02:01:01G**	2.69E-15	3.03E-16	2.39E-14	16	Lower probability		
**04:08:01**	**03:01:01G**	2.69E-15	3.03E-16	2.39E-14	16	Lower probability	*DRB1**04-*DQB1**03:01 increased risk of HR-HPV infections and transient infections	[1]

**04:10:01G**	**04:02:01G**	4.02E-14	4.79E-15	3.37E-13	45	Lower probability		
**04:10:01G**	**04:02:01G**	6.45E-13	8.02E-14	5.19E-12	58	Lower probability		
**04:11:01**	**02:01:01G**	7.47E-17	9.48E-18	5.89E-16	16	Lower probability		
**04:11:01**	**02:01:01G**	4.27E-14	2.42E-15	7.54E-13	18	Lower probability		
**04:11:01**	**02:01:01G**	8.96E-17	1.10E-17	7.32E-16	31	Lower probability		
**04:11:01**	**02:01:01G**	4.18E-15	5.31E-16	3.28E-14	45	Lower probability		
**04:11:01**	**02:01:01G**	1.94E-16	2.29E-17	1.63E-15	58	Lower probability		
**04:11:01**	**03:02:01G**	1.63E-15	5.06E-16	5.25E-15	31	Lower probability		
**04:11:01**	**03:02:01G**	5.00E-19	1.42E-19	1.75E-18	45	Lower probability		
**04:11:01**	**03:02:01G**	9.36E-17	3.26E-17	2.68E-16	58	Lower probability		
**04:11:01**	**04:02:01G**	6.36	2.35	17.22	18	Greater probability		
**04:11:01**	**04:02:01G**	1.17E-15	1.39E-16	9.89E-15	31	Lower probability		
**04:11:01**	**04:02:01G**	2.40E-19	2.85E-20	2.02E-18	58	Lower probability		
**07:01:01G**	**05:01:01G**	9.31E-18	2.31E-18	3.75E-17	18	Lower probability		
**07:11**	**03:02:01G**	7.47E-17	9.48E-18	5.89E-16	16	Lower probability		
**07:11**	**03:02:01G**	4.27E-14	2.42E-15	7.54E-13	18	Lower probability		
**07:11**	**03:02:01G**	8.96E-17	1.10E-17	7.32E-16	31	Lower probability	*DRB1**07-*DQB1**03 higher risk of CC and HSIL	[2]
**07:11**	**03:02:01G**	4.18E-15	5.31E-16	3.28E-14	45	Lower probability		
**07:11**	**03:02:01G**	1.94E-16	2.29E-17	1.63E-15	58	Lower probability		
**07:11**	**04:02:01G**	7.84E-16	1.01E-16	6.08E-15	16	Lower probability		
**07:11**	**04:02:01G**	20.29	8.25	49.93	18	Greater probability		
**07:11**	**04:02:01G**	7.65	3.18	837.74	31	Greater probability		
**07:11**	**04:02:01G**	3.25*	2.49	4.02	58	Later occurrence		
**08:01:01G**	**03:01:01G**	1.93E-16	4.17E-17	8.98E-16	18	Lower probability		
**08:01:01G**	**03:01:01G**	4.34E-19	9.48E-20	1.98E-18	31	Lower probability		
**08:01:01G**	**04:02:01G**	5.71E-16	1.58E-16	2.07E-15	31	Lower probability	*DRB1**08-*DQB1**04 higher risk of CC and HSIL	[2]
**08:02:01G**	**03:01:01G**	20.31	4.38	94.17	33	Greater probability		
**08:02:01G**	**05:01:01G**	2.32E-15	2.64E-16	2.05E-14	18	Lower probability		
**08:02:01G**	**05:01:01G**	7.52E-14	9.37E-15	6.04E-13	31	Lower probability		
**08:02:01G**	**05:01:01G**	5.44E-17	5.31E-18	5.57E-16	33	Lower probability		
**08:02:01G**	**05:01:01G**	2.01E-15	2.33E-16	1.73E-14	45	Lower probability		

**08:02:01G**	**05:01:01G**	1.60E-13	2.03E-14	1.27E-12	58	Lower probability		
**08:04:01**	**03:01:01G**	4.45E-19	1.18E-19	1.67E-18	31	Lower probability		
**08:04:01**	**03:02:01G**	8.53	4.05	18.00	16	Greater probability		
**08:04:01**	**03:02:01G**	1.46E-16	3.36E-17	6.37E-16	18	Lower probability		
**08:04:01**	**03:02:01G**	1.36E-16	2.70E-17	6.87E-16	31	Lower probability		
**08:04:01**	**03:02:01G**	2.14E-17	2.41E-18	1.89E-16	45	Lower probability		
**08:04:01**	**05:01:01G**	4.21	2.05	8.65	16	Greater probability		
**08:04:01**	**05:01:01G**	4.50E-19	4.81E-20	4.21E-18	31	Lower probability		
**08:04:01**	**05:01:01G**	5.53	2.75	1.11	45	Greater probability		
**08:07**	**04:02:01G**	3.38E-18	3.62E-19	3.16E1-7	33	Lower probability	*DRB1**08:07-04:02 increased risk of HPV-16 infection and persistence	[1, 2]
**08:07**	**04:02:01G**	4.57E-17	5.28E-18	3.96E-16	45	Lower probability		
**09:01:02G**	**03:01:01G**	8.46	3.12	22.95	18	Greater probability		
**09:01:02G**	**03:01:01G**	5.38E-15	1.17E-15	2.48E-14	31	Lower probability		
**09:01:02G**	**03:01:01G**	23.73	7.92	71.10	45	Greater probability		
**09:01:02G**	**03:01:01G**	3.27*	2.76	3.77	58	Later occurrence		
**11:02:01**	**03:01:01G**	2.56E-16	6.52E-17	1.01E-15	31	Lower probability		
**11:02:01**	**05:01:01G**	9.46	4.48	19.98	18	Greater probability		
**11:02:01**	**05:01:01G**	8.97E-16	1.12E-16	7.17E-15	31	Lower probability		
**11:04:01G**	**02:01:01G**	4.73E-15	7.81E-16	2.87E-14	16	Lower probability		
**11:04:01G**	**02:01:01G**	7.54E-15	9.53E-16	5.96E-14	31	Lower probability		
**11:04:01G**	**02:01:01G**	5.97E-17	1.68E-17	2.13E-16	58	Lower probability		
**11:04:01G**	**03:02:01G**	1.11E-16	1.16E-17	1.07E-15	18	Lower probability	*DRB1**11-*DQB1**03 lower risk of CC and HSIL, higher risk in Amerindians and Senegalese<br>*DRB1**11:02-*DQB1**03:01 lower risk of persistence	[1, 2, 11]
**11:04:01G**	**03:02:01G**	-1.06*	-1.50	-0.62	58	Earlier occurrence		
**11:04:01G**	**04:02:01G**	1.31E-14	1.51E-15	1.13E-13	31	Lower probability		
**11:04:01G**	**04:02:01G**	1.84E-17	2.26E-18	1.49E-16	45	Lower probability		
**11:04:01G**	**04:02:01G**	1.53E-18	1.78E-19	1.32E-17	58	Lower probability		
**11:04:01G**	**05:01:01G**	1.33E-19	2.52E-20	7.03E-19	16	Lower probability		
**11:04:01G**	**05:01:01G**	1.70E-14	1.85E-19	1.56E-17	18	Lower probability		
**11:04:01G**	**06:03:01G**	2.29E-15	2.77E-16	1.90E-14	58	Lower probability		
**11:14:01**	**04:02:01G**	3.18E-15	3.79E-16	2.67E-14	31	Lower probability		
**11:14:01**	**04:02:01G**	1.09E-16	1.39E-17	8.53E-16	45	Lower probability		

**11:14:01**	**04:02:01G**	4.07*	3.35	4.80	58	Later occurrence		
**12:01:01G**	**03:02:01G**	4.80	2.66	8.64	16	Greater probability		
**12:01:01G**	**03:02:01G**	6.92E-18	6.79E-19	7.05E-17	18	Lower probability		
**12:01:01G**	**03:02:01G**	5.55E-19	1.13E-19	2.72E-18	31	Lower probability		
**12:01:01G**	**03:02:01G**	12.65	4.93	32.45	45	Greater probability		
**12:01:01G**	**03:02:01G**	2.75E-19	5.99E-20	1.26E-18	58	Lower probability		
**13:01:01G**	**03:01:01G**	2.09E-18	5.74E-19	7.58E-18	18	Lower probability		
**13:01:01G**	**03:01:01G**	4.09E-16	9.32E-17	1.79E-15	31	Lower probability	*DRB1**13:03-*DQB1**03:01 lower risk of CC and HSIL	[2, 11, 12, 14]
**13:01:01G**	**03:01:01G**	3.46	2.13	5.63	45	Greater probability		
**13:01:01G**	**05:02:01G**	3.35E-17	4.13E-18	2.71E-18	18	Lower probability		
**13:01:01G**	**05:02:01G**	1.64E-15	2.10E-16	1.28E-14	31	Lower probability		
**13:01:01G**	**06:02:01G**	27.22	6.58	112.44	18	Greater probability		
**13:02:01G**	**03:01:01G**	4.34*	3.69	4.99	31	Later occurrence		
**13:02:01G**	**05:01:01G**	3.38E-15	4.41E-16	2.59E-14	16	Lower probability		
**13:02:01G**	**05:01:01G**	1.23E-15	2.61E-16	5.79E-15	18	Lower probability		
**13:02:01G**	**05:01:01G**	-1.29*	-1.74	-0.85	31	Earlier occurrence		
**13:02:01G**	**05:01:01G**	1.37E-15	1.73E-16	1.08E-14	45	Lower probability		
**13:02:01G**	**06:02:01G**	2.76E-19	3.47E-20	2.20E-18	16	Lower probability		
**13:02:01G**	**06:02:01G**	-1.17*	-1.67	-0.67	31	Earlier occurrence	*DRB1**13-*DQB1**06 lower risk of CC<br>*DRB1**13:01-*DQB1**06:03 lower risk of CC	[2, 11, 12, 14]
**13:02:01G**	**06:02:01G**	5.35	2.39	1.19	45	Greater probability		
**13:02:01G**	**06:04:01G**	4.87E-16	1.09E-16	2.17E-15	18	Lower probability		
**13:03:01G**	**03:01:01G**	2.29E-16	4.88E-17	1.08E-15	18	Lower probability		
**13:03:01G**	**03:01:01G**	22.15	8.33	58.85	45	Greater probability		
**13:03:01G**	**04:02:01G**	4.54*	3.88	5.20	31	Later occurrence		
**13:03:01G**	**05:01:01G**	-1.33*	-1.82	-0.84	31	Earlier occurrence		
**13:03:01G**	**05:01:01G**	22.15	8.33	58.85	45	Greater probability		
**13:03:01G**	**05:01:01G**	-1.07*	-1.34	-0.80	58	Earlier occurrence		
**13:04**	**03:01:01G**	2.75E-17	4.00E-18	1.90E-16	18	Lower probability		
**13:04**	**03:01:01G**	1.19E-14	2.24E-15	6.27E-14	31	Lower probability		
**13:04**	**03:01:01G**	2.74*	2.10	3.38	58	Later occurrence		
**13:04**	**04:02:01G**	7.84E-16	3.56E-17	2.26E-15	16	Lower probability		

**13:04**	**04:02:01G**	1.92E-15	1.75E-16	2.12E-14	18	Lower probability		
**13:04**	**04:02:01G**	2.24E-16	2.51E-17	2.00E-15	31	Lower probability		
**13:04**	**04:02:01G**	2.74*	2.10	3.38	58	Later occurrence		
**13:05:01**	**03:01:01G**	3.21	1.68	6.11	16	Greater probability		
**13:05:01**	**03:01:01G**	4.19*	3.08	5.31	31	Later occurrence		
**13:05:01**	**03:01:01G**	5.97E-16	9.02E-17	3.95E-15	58	Lower probability		
**13:05:01**	**03:02:01G**	3.21	1.68	6.11	16	Greater probability		
**13:05:01**	**03:02:01G**	4.66E-18	8.06E-19	2.70E-17	18	Lower probability		
**13:05:01**	**03:02:01G**	4.78*	3.65	5.92	31	Later occurrence		
**13:05:01**	**03:02:01G**	2.90E-15	3.28E-16	2.56E-14	45	Lower probability		
**13:05:01**	**03:02:01G**	3.24E-17	3.62E-18	2.91E-16	58	Lower probability		
**14:01:01G**	**05:03:01G**	3.83E-16	4.52E-17	3.24E-15	33	Lower probability		
**14:02:01G**	**04:02:01G**	158.36	31.24	802.67	18	Greater probability		
**14:02:01G**	**04:02:01G**	-0.95*	-1.30	-0.59	58	Earlier occurrence		
**14:02:01G**	**05:01:01G**	5.67E-18	7.09E-19	4.54E-17	18	Lower probability		
**14:02:01G**	**05:01:01G**	6.63E-15	8.29E-16	5.30E-14	31	Lower probability		
**14:02:01G**	**05:01:01G**	2.77*	2.17	3.36	58	Later occurrence		
**14:02:01G**	**06:04:01G**	1.69E-18	1.96E-19	1.46E-17	16	Lower probability		
**15:01:01G**	**05:02:01G**	7.86E-15	8.94E-15	6.90E-14	16	Lower probability		
**15:01:01G**	**05:02:01G**	-1.54*	-2.01	-1.08	31	Earlier occurrence		
**15:01:01G**	**05:02:01G**	8.58	2.75	2.67	33	Greater probability		
**15:01:01G**	**05:02:01G**	3.51E-14	4.12E-15	2.98E-13	58	Lower probability		
**15:01:01G**	**06:03:01G**	27.22	6.58	112.44	18	Greater probability	*DRB1**15:01-*DQB1**06:02 lower risk of CC plus HPV-16 in Asian population, higher risk of CC in Hispanic and Amerindian population *DRB1**15:03-*DQB1**06:02 higher risk of CC and HSIL	[2, 7, 10, 15, 16]
**15:02:01G**	**06:01:01G**	2.52E-18	5.37E-19	1.18E-17	18	Lower probability		
**15:03:01G**	**06:02:01G**	2.02	8.61	47.54	16	Greater probability		
**15:03:01G**	**06:02:01G**	6.96E-18	1.39E-18	3.47E-17	18	Lower probability		
**15:03:01G**	**06:02:01G**	4.15E-17	6.69E-18	2.57E-16	33	Lower probability		
**16:01:01**	**03:01:01G**	4.53	1.97	10.38	16	Greater probability		
**16:01:01**	**03:01:01G**	1.19E-13	1.20E-14	1.18E-12	18	Lower probability		
**16:01:01**	**03:01:01G**	4.43E-14	5.34E-15	3.67E-13	31	Lower probability		
**16:01:01**	**03:01:01G**	9.04	3.92	2.08	45	Greater probability		

**16:01:01**	**03:01:01G**	2.30*	1.50	3.09	58	Later occurrence		
**16:01:01**	**06:03:01G**	3.35E-17	4.13E-18	2.71E-16	18	Lower probability		
**16:01:01**	**06:03:01G**	1.64E-15	2.10E-16	1.28E-14	31	Lower probability		
**16:02:01G**	**03:03:02G**	12.15	4.57	32.34	18	Greater probability		
**16:02:01G**	**03:03:02G**	4.26E-14	5.33E-15	3.41E-13	31	Lower probability		
**16:02:01G**	**03:03:02G**	2.83E-15	3.65E-16	2.20E-14	58	Lower probability		
**16:02:01G**	**04:02:01G**	3.51	1.74	7.09	16	Greater probability		
**16:02:01G**	**04:02:01G**	4.31E-17	4.63E-18	4.01E-16	31	Lower probability		
**16:02:01G**	**04:02:01G**	1.02E-13	6.26E-15	1.66E-12	45	Lower probability		
**16:02:01G**	**04:02:01G**	3.22E-18	3.96E-19	2.61E-17	58	Lower probability		
**16:02:01G**	**05:01:01G**	5.54E-15	7.05E-16	4.36E-14	16	Lower probability		
**16:02:01G**	**05:01:01G**	-1.15*	-1.58	-0.73	45	Earlier occurrence		
**16:02:01G**	**05:02:01G**	2.23E-15	3.10E-16	1.60E-14	18	Lower probability	*DRB1**16-*DQB1**05 lower risk of CC and HSIL	[2]
**16:02:01G**	**05:02:01G**	4.05*	3.41	4.70	31	Later occurrence		
**16:02:01G**	**05:02:01G**	3.07*	2.50	3.64	58	Later occurrence		
**16:02:01G**	**06:03:01G**	9.34E-17	2.06E-17	4.23E-16	18	Lower probability		
**16:02:01G**	**06:03:01G**	6.35E-17	7.72E-18	5.22E-16	31	Lower probability		
**16:02:01G**	**06:03:01G**	3.83	1.86	7.88	45	Greater probability		
**16:02:01G**	**06:03:01G**	8.62E-18	9.52E-19	7.80E-17	58	Lower probability		
**16:02:01G**	**06:09:01G**	6.61E-14	6.63E-15	6.60E-13	16	Lower probability		
**16:02:01G**	**06:09:01G**	7.99E-16	9.42E-17	26.78E-15	18	Lower probability		
**16:02:01G**	**06:09:01G**	5.43E-15	6.40E-16	4.61E-14	31	Lower probability		
**16:02:01G**	**06:09:01G**	6.80E-18	8.01E-19	5.77E-17	45	Lower probability		
**01:01:01G**	**03:01:01G**	2.00E-16	3.72E-17	1.08E-15	18	Lower probability		

95%CI: 95% confidence interval, Coeff: regression coefficient (hazard ratio for Cox proportional hazards regression model and time ratio for log-normal parametric model), *DRB1: DR beta 1, DQB1: DQ beta 1,* HR-HPV: high-risk human papillomavirus, SIL: squamous intraepithelial lesion, HSIL: high-grade squamous intraepithelial lesion, CC: cervical cancer

[a] *p*: *p* value was corrected using the Bonferroni method

[b] The Cox proportional hazards model did not fulfil the assumption of proportionality; the log-normal parametric model was used in such cases

Supplementary Table S15. DRB1 and DQB1 haplotypes associated with HR-HPV redetection

Haplotype		Coeff. [a]	95%CI		HR-HPV type	Effect on redetection
DRB1	*DQB1*		Lower	Lower		
**01:01:01G**	**03:01:01G**	1.55	1.27	1.82	16	Later occurrence
**01:01:01G**	**03:01:01G**	3.32	2.71	3.92	45	Later occurrence
**01:01:01G**	**03:01:01G**	3.67E-15[b]	6.14E-16	2.19E-14	58	Lower probability
**01:01:01G**	**03:02:01G**	4.73E-18[b]	1.01E-18	2.21E-17	31	Lower probability
**01:01:01G**	**03:03:02G**	2.40	1.89	2.92	45	Later occurrence
**01:02:01G**	**02:01:01G**	7.82E-17	5.93E-18	1.03E-15	18	Earlier occurrence
**01:02:01G**	**02:01:01G**	3.84[b]	1.92	7.68	31	Greater probability
**01:02:01G**	**02:01:01G**	3.40E-17[b]	3.97E-18	2.91E-16	58	Lower probability
**01:02:01G**	**03:01:01G**	2.93	2.36	3.50	45	Later occurrence
**01:02:01G**	**03:01:01G**	2.31	1.64	2.98	58	Later occurrence
**01:02:01G**	**03:03:02G**	1.40	1.12	1.68	16	Later occurrence
**01:02:01G**	**03:03:02G**	2.74E-14	3.29e-15	2.28E-13	18	Earlier occurrence
**01:02:01G**	**03:03:02G**	1.08	0.48	1.68	45	Later occurrence
**01:03:01**	**02:01:01G**	5.54E-16	6.46E-17	4.75E-15	18	Earlier occurrence
**01:03:01**	**03:01:01G**	-0.51	-0.69	-0.33	16	Earlier occurrence
**01:03:01**	**03:01:01G**	2.71	2.16	3.27	45	Later occurrence
**01:03:01**	**03:01:01G**	3.48	2.91	4.06	58	Later occurrence
**01:03:01**	**03:02:01G**	8.50E-16	8.91E-17	8.11E-15	18	Earlier occurrence
**01:03:01**	**03:02:01G**	2.96	2.39	3.53	45	Later occurrence
**01:03:01**	**03:02:01G**	-0.85	-1.29	-0.40	58	Earlier occurrence
**01:03:01**	**04:02:01G**	1.47E-16	1.80E-17	1.20E-15	18	Earlier occurrence
**01:03:01**	**04:02:01G**	3.06	2.53	3.60	45	Later occurrence
**01:03:01**	**05:01:01G**	3.30	2.81	3.79	45	Later occurrence
**03:01:01G**	**05:01:01G**	2.42	1.68	3.15	58	Later occurrence
**03:02:01**	**04:02:01G**	1.40E-15	1.80E-16	1.08E-14	18	Earlier occurrence
**03:02:01**	**04:02:01G**	2.63	2.24	3.02	31	Later occurrence
**03:02:02**	**02:01:01G**	8.23E-15	7.21E-16	9.41E-14	18	Earlier occurrence
**03:02:02**	**02:01:01G**	1.87	1.36	2.37	31	Later occurrence
**03:02:02**	**02:01:01G**	1.66	0.86	2.46	58	Later occurrence
**03:02:02**	**03:01:01G**	4.23E-20	.	.	18	Earlier occurrence
**03:02:02**	**03:01:01G**	1.92	1.55	2.29	31	Later occurrence
**03:02:02**	**03:01:01G**	1.80	1.04	2.56	58	Later occurrence
**04:02:01**	**02:01:01G**	1.09	0.51	1.67	45	Later occurrence
**04:02:01**	**02:01:01G**	1.05	0.55	1.55	58	Later occurrence
**04:02:01**	**03:01:01G**	0.43	-0.68	-0.19	31	Earlier occurrence
**04:02:01**	**03:01:01G**	2.08	2.37	3.41	45	Later occurrence

**04:02:01**	**03:02:01G**	0.44	-0.66	-0.20	58	Earlier occurrence
**04:03:01G**	**02:01:01G**	1.98	1.58	2.38	31	Later occurrence
**04:03:01G**	**02:01:01G**	2.49	1.90	3.09	58	Later occurrence
**04:03:01G**	**03:01:01G**	5.98E-16	6.44E-17	5.55E-15	18	Earlier occurrence
**04:03:01G**	**03:01:01G**	2.01	1.34	2.69	58	Later occurrence
**04:03:01G**	**03:02:01G**	2.74	2.12	3.36	58	Later occurrence
**04:04:01**	**02:01:01G**	0.30	0.15	0.46	16	Earlier occurrence
**04:04:01**	**02:01:01G**	1.07	0.52	1.83	45	Later occurrence
**04:04:01**	**02:01:01G**	-0.93	-1.37	-0.49	58	Earlier occurrence
**04:04:01**	**03:01:01G**	1.01	0.65	1.72	45	Later occurrence
**04:04:01**	**05:01:01G**	-1.02	-1.24	-0.80	18	Earlier occurrence
**04:04:01**	**05:01:01G**	-0.70	-1.02	-0.38	45	Earlier occurrence
**04:05:01**	**02:01:01G**	1.81	1.37	2.26	31	Later occurrence
**04:05:01**	**02:01:01G**	-1.05	-1.41	-0.69	45	Earlier occurrence
**04:05:01**	**03:01:01G**	1.58	1.28	1.88	16	Later occurrence
**04:05:01**	**03:01:01G**	2.59	2.01	3.18	58	Later occurrence
**04:05:04**	**02:01:01G**	-0.44	-0.68	-0.20	16	Earlier occurrence
**04:05:04**	**02:01:01G**	1.21E-17	1.43E-18	1.02E-16	18	Earlier occurrence
**04:05:04**	**02:01:01G**	2.93	2.25	3.61	58	Later occurrence
**04:05:04**	**03:01:01G**	1.61	1.34	1.88	16	Later occurrence
**04:05:04**	**03:01:01G**	2.78E-14	3.58E-15	2.15E-13	18	Earlier occurrence
**04:05:04**	**03:02:01G**	1.25	0.59	1.91	45	Later occurrence
**04:05:04**	**03:02:01G**	2.11	1.37	2.85	58	Later occurrence
**04:05:04**	**04:02:01G**	2.92	2.24	3.59	58	Later occurrence
**04:05:04**	**05:01:01G**	1.47E-16	1.80E-17	1.20E-15	18	Earlier occurrence
**04:05:04**	**05:01:01G**	3.06	2.53	3.60	45	Later occurrence
**04:07:01G**	**02:01:01G**	0.38	0.20	0.55	16	Earlier occurrence
**04:07:01G**	**02:01:01G**	3.30	2.65	3.95	58	Later occurrence
**04:07:01G**	**03:02:03**	7.97E-15	9.98E-16	6.37E-14	18	Earlier occurrence
**04:07:01G**	**03:02:03**	-0.71	-1.04	-0.38	45	Earlier occurrence
**04:07:01G**	**03:02:03**	-1.00	-1.47	-0.53	58	Earlier occurrence
**04:08:01**	**02:01:01G**	5.46E-17	1.09E-17	2.75E-16	18	Earlier occurrence
**04:08:01**	**02:01:01G**	2.12	1.73	2.52	31	Later occurrence
**04:08:01**	**02:01:01G**	2.27	1.77	2.76	45	Later occurrence
**04:08:01**	**02:01:01G**	3.47	2.97	3.96	58	Later occurrence
**04:08:01**	**03:01:01G**	5.46E-17	1.09E-17	2.75E-16	18	Earlier occurrence
**04:08:01**	**03:01:01G**	2.12	1.73	2.52	31	Later occurrence
**04:08:01**	**03:01:01G**	2.27	1.77	2.76	45	Later occurrence
**04:08:01**	**03:01:01G**	3.47	2.97	3.96	58	Later occurrence
**04:11:01**	**02:01:01G**	0.67	-0.97	-0.37	31	Earlier occurrence
**04:11:01**	**03:02:01G**	0.67	-0.96	-0.37	31	Earlier occurrence

**04:11:01**	**04:02:01G**	1.54	1.20	1.87	16	Later occurrence
**07:01:01G**	**03:01:01G**	1.30	1.01	1.59	16	Later occurrence
**07:01:01G**	**03:01:01G**	5.69E-16	1.81E-16	1.79E-15	18	Earlier occurrence
**07:01:01G**	**03:01:01G**	2.61	2.12	3.09	45	Later occurrence
**07:01:01G**	**03:01:01G**	3.49	3.00	3.98	58	Later occurrence
**07:01:01G**	**03:02:01G**	2.50	2.18	2.97	31	Later occurrence
**07:01:01G**	**03:02:01G**	2.89	2.24	3.54	58	Later occurrence
**07:01:01G**	**03:03:02G**	2.40	1.89	2.92	45	Later occurrence
**07:01:01G**	**04:02:01G**	2.40	1.82	2.97	45	Later occurrence
**07:01:01G**	**04:02:01G**	2.95	2.27	3.62	58	Later occurrence
**07:01:01G**	**05:01:01G**	1.40	1.12	1.68	16	Later occurrence
**07:01:01G**	**05:01:01G**	2.02E-15	4.94E-16	8.27E-15	18	Earlier occurrence
**07:01:01G**	**05:01:01G**	2.40	1.89	2.92	45	Later occurrence
**07:11**	**03:02:01G**	7.29	0.97	-0.37	31	Later occurrence
**07:11**	**04:02:01G**	4.25E-15	5.03E-16	3.59E-14	18	Earlier occurrence
**07:11**	**04:02:01G**	16.70	1.71	26.19	31	Later occurrence
**08:01:01G**	**03:01:01G**	-0.70	-0.99	-0.42	31	Earlier occurrence
**08:01:01G**	**03:01:01G**	2.83	2.31	3.34	45	Later occurrence
**08:01:01G**	**03:01:01G**	2.05	1.40	2.70	58	Later occurrence
**08:01:01G**	**04:02:01G**	-0.70	-0.99	-0.42	31	Earlier occurrence
**08:01:01G**	**04:02:01G**	2.83	2.31	3.34	45	Later occurrence
**08:01:01G**	**04:02:01G**	2.05	1.40	2.70	58	Later occurrence
**08:02:01G**	**04:02:01G**	-0.51	-0.80	-0.23	45	Earlier occurrence
**08:04:01**	**03:01:01G**	2.85	2.27	3.43	58	Later occurrence
**08:04:01**	**03:02:01G**	1.51E-19	2.71E-20	8.37E-19	18	Earlier occurrence
**08:04:01**	**03:02:01G**	-0.63	-0.97	-0.29	45	Earlier occurrence
**08:04:01**	**03:02:01G**	2.77	2.09	3.46	58	Later occurrence
**08:04:01**	**05:01:01G**	2.93	2.36	3.50	45	Later occurrence
**08:04:01**	**05:01:01G**	2.32	1.74	2.90	58	Later occurrence
**08:07**	**04:02:01G**	2.51	0.89	3.13	58	Later occurrence
**09:01:02G**	**03:01:01G**	2.50	1.99	3.00	45	Later occurrence
**11:01:01G**	**03:01:01G**	2.73	2.33	3.12	31	Later occurrence
**11:01:01G**	**03:02:01G**	2.61	2.22	3.00	31	Later occurrence
**11:01:02**	**05:01:01G**	1.62E-16	1.95E-17	1.34E-15	18	Earlier occurrence
**11:01:02**	**05:01:01G**	2.65	1.72	3.58	58	Later occurrence
**11:02:01**	**05:01:01G**	1.40	1.11	1.68	16	Later occurrence
**11:02:01**	**05:01:01G**	1.62E-16	1.95E-17	1.34E-15	18	Earlier occurrence
**11:02:01**	**05:01:01G**	2.37	1.42	3.31	58	Later occurrence
**11:04:01G**	**02:01:01G**	7.10E-16	1.12E-16	4.52E-15	18	Earlier occurrence
**11:04:01G**	**02:01:01G**	2.12	1.58	2.65	45	Later occurrence
**11:04:01G**	**03:02:01G**	2.15	1.61	2.68	45	Later occurrence

**11:04:01G**	**03:02:01G**	2.88	2.33	3.43	58	Later occurrence
**11:04:01G**	**04:02:01G**	1.18	0.58	1.78	45	Later occurrence
**11:04:01G**	**05:01:01G**	-0.51	-0.69	-0.33	16	Earlier occurrence
**11:04:01G**	**05:01:01G**	6.90E-18	5.33E-19	8.94E-17	18	Earlier occurrence
**11:04:01G**	**05:01:01G**	2.41	1.89	2.94	45	Later occurrence
**11:04:01G**	**05:01:01G**	3.21	2.66	3.75	58	Later occurrence
**11:04:01G**	**06:03:01G**	2.12	1.58	2.65	45	Later occurrence
**11:14:01**	**04:02:01G**	0.70	-0.99	-0.42	31	Earlier occurrence
**11:14:01**	**04:02:01G**	-0.87	-1.31	-0.43	45	Earlier occurrence
**12:01:01G**	**03:02:01G**	1.47	1.19	1.75	16	Later occurrence
**12:01:01G**	**03:02:01G**	2.31	8.87	6.06	18	Later occurrence
**12:01:01G**	**03:02:01G**	2.84	2.33	3.36	45	Later occurrence
**13:01:01G**	**03:01:01G**	-0.65	-0.90	-0.39	31	Earlier occurrence
**13:01:01G**	**03:01:01G**	2.07	1.40	2.74	58	Later occurrence
**13:01:01G**	**05:02:01G**	-0.61	-0.83	-0.39	16	Earlier occurrence
**13:01:01G**	**05:02:01G**	2.11E-17	2.73E-18	1.64E-16	18	Earlier occurrence
**13:01:01G**	**05:02:01G**	2.68	2.22	3.14	45	Later occurrence
**13:01:01G**	**05:02:01G**	2.22	1.32	3.12	58	Later occurrence
**13:01:01G**	**06:02:01G**	4.24E-17	4.75E-18	3.79E-16	18	Earlier occurrence
**13:01:01G**	**06:02:01G**	2.50	1.77	3.24	58	Later occurrence
**13:02:01G**	**03:01:01G**	4.93E-17	8.60E-18	2.83E-16	18	Earlier occurrence
**13:02:01G**	**05:01:01G**	-0.57	-0.72	-0.42	16	Earlier occurrence
**13:02:01G**	**05:01:01G**	2.18E-14	2.69E-15	1.78E-13	18	Earlier occurrence
**13:02:01G**	**05:01:01G**	2.14	1.76	2.52	31	Later occurrence
**13:02:01G**	**06:02:01G**	1.21E-15	1.35E-16	1.08E-14	18	Earlier occurrence
**13:02:01G**	**06:02:01G**	2.53	2.13	2.93	31	Later occurrence
**13:02:01G**	**06:02:01G**	2.87	2.34	3.40	45	Later occurrence
**13:02:01G**	**06:04:01G**	2.71	2.13	3.29	31	Later occurrence
**13:03:01G**	**03:01:01G**	1.58	1.31	1.85	16	Later occurrence
**13:03:01G**	**03:01:01G**	2.37	1.75	3.00	45	Later occurrence
**13:03:01G**	**03:01:01G**	2.41	1.84	2.99	58	Later occurrence
**13:03:01G**	**04:02:01G**	1.58	1.31	1.85	16	Later occurrence
**13:03:01G**	**04:02:01G**	4.92E-18	6.19E-19	3.91E-17	18	Earlier occurrence
**13:03:01G**	**04:02:01G**	-0.70	-0.99	-0.42	31	Earlier occurrence
**13:03:01G**	**04:02:01G**	2.05	1.40	2.70	58	Later occurrence
**13:03:01G**	**05:01:01G**	2.24	1.83	2.65	31	Later occurrence
**13:03:01G**	**05:01:01G**	2.37	1.75	3.00	45	Later occurrence
**13:03:01G**	**05:01:01G**	2.41	1.83	2.99	58	Later occurrence
**13:04**	**03:01:01G**	2.83	2.31	3.34	45	Later occurrence
**13:04**	**04:02:01G**	2.83	2.31	3.34	45	Later occurrence
**13:05:01**	**03:01:01G**	1.13	0.85	1.41	16	Later occurrence

**13:05:01**	**03:01:01G**	2.50	1.99	3.00	45	Later occurrence
**13:05:01**	**03:02:01G**	1.13	0.85	1.41	16	Later occurrence
**13:05:01**	**03:02:01G**	9.26E-18	1.06E-18	8.10E-17	18	Earlier occurrence
**14:01:01G**	**05:03:01G**	1.83E-16[b]	3.47E-17	9.67E-16	16	Lower probability
**14:02:01G**	**04:02:01G**	2.27	1.76	2.77	31	Later occurrence
**14:02:01G**	**04:02:01G**	2.30	1.68	2.92	45	Later occurrence
**14:02:01G**	**04:02:01G**	2.14	1.54	2.74	58	Later occurrence
**14:02:01G**	**05:01:01G**	1.55	1.27	1.82	16	Later occurrence
**14:02:01G**	**06:04:01G**	1.96	1.37	2.55	58	Later occurrence
**15:01:01G**	**05:02:01G**	1.40E-15	1.80E-16	1.08E-14	18	Earlier occurrence
**15:01:01G**	**05:02:01G**	2.63	2.24	3.02	31	Later occurrence
**15:01:01G**	**06:03:01G**	4.24E-17	4.75E-18	3.79E-16	18	Earlier occurrence
**15:01:01G**	**06:03:01G**	2.50	1.77	3.24	58	Later occurrence
**15:02:01G**	**06:01:01G**	2.73	2.18	3.27	31	Later occurrence
**15:02:01G**	**06:01:01G**	2.75	2.14	3.37	58	Later occurrence
**15:03:01G**	**06:02:01G**	2.16	1.51	2.80	45	Later occurrence
**15:03:01G**	**06:02:01G**	3.25	2.75	3.75	58	Later occurrence
**16:01:01**	**03:01:01G**	1.22	0.90	1.54	16	Later occurrence
**16:01:01**	**03:01:01G**	1.85	1.16	2.66	18	Later occurrence
**16:01:01**	**03:01:01G**	2.04	1.88	2.92	45	Later occurrence
**16:01:01**	**05:02:01G**	2.07	2.30	3.17	45	Later occurrence
**16:01:01**	**05:02:01G**	3.10	2.45	3.75	58	Later occurrence
**16:01:01**	**06:03:01G**	-0.61	-0.83	-0.39	16	Earlier occurrence
**16:01:01**	**06:03:01G**	2.11E-17	2.73E-18	1.64E-16	18	Earlier occurrence
**16:01:01**	**06:03:01G**	2.06	2.22	3.14	45	Later occurrence
**16:01:01**	**06:03:01G**	2.22	1.32	3.12	58	Later occurrence
**16:02:01G**	**04:02:01G**	1.61	1.34	1.88	16	Later occurrence
**16:02:01G**	**04:02:01G**	2.78E-14	3.58E-15	2.15E-13	18	Earlier occurrence
**16:02:01G**	**05:01:01G**	2.36	1.93	2.78	31	Later occurrence
**16:02:01G**	**05:01:01G**	3.32	2.71	3.92	45	Later occurrence
**16:02:01G**	**05:01:01G**	1.90	1.16	2.64	58	Later occurrence
**16:02:01G**	**05:02:01G**	2.40	1.88	2.92	45	Later occurrence
**16:02:01G**	**05:02:01G**	1.90	1.16	2.64	58	Later occurrence
**16:02:01G**	**06:03:01G**	1.67	1.38	1.97	16	Later occurrence
**16:02:01G**	**06:03:01G**	-0.65	-0.90	-0.39	31	Earlier occurrence
**16:02:01G**	**06:03:01G**	3.50	3.03	3.97	45	Later occurrence
**16:02:01G**	**06:09:01G**	2.14E-14	2.42E-15	1.89E-13	18	Earlier occurrence
**16:02:01G**	**06:09:01G**	1.98	1.17	2.80	58	Later occurrence

95%CI: 95% confidence interval, Coeff: regression coefficient (hazard ratio for Cox proportional hazards regression model and time ratio for log-normal parametric model), *DRB1: DR beta 1, DQB1: DQ beta 1,* HR-HPV: high-risk human papillomavirus.

[a] The Cox proportional hazards model did not fulfil the assumption of proportionality; the log-normal parametric model was used in such cases

[b] The Cox proportional hazards model was used.

Supplementary Table S16. MHC-II binding epitopes from the L1 protein of HR-HPV related to clearance/persistence events.

Locus	Allele	HPV	Effect on clearance/persistence	Number of peptide	Peptides	Core	%Rank	Analysis
	DRB1*01:03	31	Earlier occurrence	1	VTRTNIYYHAGSARLLTVGH	YYHAGSARL	0.08	The peptides may be related to the events described
				2	FPLGRKFLLQAGYRARPKFK	FLLQAGYRA	0.6	
				3	ITLSADIMTYIHSMNPAILE	YIHSMNPAI	1.3	
		45	Greater probability	1	YVSRTSIFYHAGSSRLLTVG	FYHAGSSRL	0.1	
				2	QYPLGRKFLVQAGLRRRPTI	FLVQAGLRR	3.0	
				3	TAEVMSYIHSMNSSILENWN	YIHSMNSSI	3.0	
	DRB1*03:02	16	Lower probability	1	-	-	-	No peptide explains the events found
		31	Earlier occurrence	1	-	-	-	
		33	Greater probability	1	-	-	-	
		45	Lower probability	1	-	-	-	
		58	Lower probability	1	CTLAILFCVADVNVFHIFLQ	CVADVNVFH	3.5	
	DRB1*04:08	16	Lower probability	1	GTLEDTYRFVTSQAIACQKH	YRFVTSQAI	1.0	The peptides 4, 5 and 6 may be related to the events described
				2	LTADVMTYIHSMNSTILEDW	YIHSMNSTI	3.0	
				3	EYVARTNIYYHAGTSRLLAV	YYHAGTSRL	4.0	
				4	TGFGAMDFTTLQANKSEVPL	FTTLQANKS	4.0	
				5	QFPLGRKFLLQAGLKAKPKF	FLLQAGLKA	4.0	
				6	-	-	-	
		18	Greater probability	1	TTSLVDTYRFVQSVAITCQK	YRFVQSVAI	3.0	
				2	LTADVMSYIHSMNSSILEDW	YIHSMNSSI	2.5	
				3	SIFYHAGSFRLLTVGNPYFR	FYHAGSFRL	2.5	
				4	-	-	-	
				5	-	-	-	
				6	GHYIILFLRNVNVFPIFLQM	FLRNVNVFP	3.0	
	DRB1*04:10	33	Lower probability	1	FFFLRREQMFVRHFFNRAGK	VRHFFNRAG	3.5	
				2	QFPLGRKFLLQAGLKAKPKL	FLLQAGLKA	3.5	
				3	-	-	-	
				4	-	-	-	
		45		1	-	-	-	
				2	-	-	-	
				3	KVSAYQYRVFRVALPDPNKF	YRVFRVALP	2.0	
				4	YGHGIIIFLKNVNVFPIFLQ	LKNVNVFPI	4.0	
	DRB1*04:11	31	Lower probability	1	QFPLGRKFLLQAGYRARPKF	FLLQAGYRA	3.5	
		45		1	-	-	-	
		58		1	FPLGRKFLLQSGLKAKPRLK	FLLQSGLKA	5.0	
	DRB1*07:11	16	Lower probability	1	EYVARTNIYYHAGTSRLLAV	YYHAGTSRL	0.6	
				2	GTLEDTYRFVTSQAIACQKH	YRFVTSQAI	1.2	
				3	KITLTADVMTYIHSMNSTIL	YIHSMNSTI	2.5	
				4	-	-	-	
		18	Greater probability	1	DYVTRTSIFYHAGSFRLLTV	FYHAGSFRL	0.1	
				2	TTSLVDTYRFVQSVAITCQK	YRFVQSVAI	1.5	
				3	ITLTADVMSYIHSMNSSILE	YIHSMNSSI	2.5	
				4	CGHYIILFLRNVNVFPIFLQ	LRNVNVFPI	4.5	
		45	Lower probability	1	DYVSRTSIFYHAGSSRLLTV	FYHAGSSRL	0.2	
				2	TTSLVDTYRFVQSVAVTCQK	TTSLVDTYRFVQSVAVTCQK	2.5	
				3	LTAEVMSYIHSMNSSILENW	YIHSMNSSI	1.9	
				4	IYGHGIIIFLKNVNVFPIFL	LKNVNVFPI	4.5	

DRB1	***DRB1*08:04***	31	Lower probability	1	LGRKFLLQAGYRARPKFKAG	FLLQAGYRA	2.5	No peptide explains the events found
		33	Earlier occurrence	1	GRKFLLQAGLKAKPKLKRAA	FLLQAGLKA	2.5	
		58	Greater probability	1	GRKFLLQSGLKAKPRLKRSA	FLLQSGLKA	2.5	
	DRB1*08:07	18	Earlier occurrence	1	GHYIILFLRNVNVFPIFLQM	GHYIILFLRNVNVFPIFLQM	1.3	
				2	-	-	-	
		31	Greater probability	1	-	-	-	
				2	ITLSADIMTYIHSMNPAILE	ITLSADIMTYIHSMNPAILE	4.5	
		33	Lower probability	1	-	-	-	
				2	-	-	-	
		45	Lower probability	1	YGHGIIIFLKNVNVFPIFLQ	LKNVNVFPI	1.1	
				2	-	-	-	
		58	Earlier occurrence	1	-	-	-	
				2	-	-	-	
	DRB1*11:02	31	Lower probability	1	PLGRKFLLQAGYRARPKFKA	FLLQAGYRA	3.5	
		45	Greater probability	1	QYPLGRKFLVQAGLRRRPTI	FLVQAGLRR	1.2	
	DRB1*11:14	18	Greater probability	1	GHYIILFLRNVNVFPIFLQM	LRNVNVFPI	4.0	
				2	ITLTADVMSYIHSMNSSILE	YIHSMNSSI	6.0	
		31	Lower probability	1	-	-	-	
				2	ITLSADIMTYIHSMNPAILE	YIHSMNPAI	3.0	
		45	Lower probability	1	YGHGIIIFLKNVNVFPIFLQ	LKNVNVFPI	4.0	
				2	VMSYIHSMNSSILENWNFGV	YIHSMNSSI	4.5	
		58	Later occurrence	1	-	-	-	
				2	-	-	-	
	DRB1*12:01	16	Greater probability	1	-	-	-	
				2	-	-	-	
		18	Lower probability	1	GHYIILFLRNVNVFPIFLQM	LRNVNVFPI	2.5	
				2	-	-	-	
		31	Lower probability	1	-	-	-	
				2	-	-	-	
		33	Greater probability	1	-	-	-	
				2	-	-	-	
		45	Greater probability	1	GHGIIIFLKNVNVFPIFLQM	LKNVNVFPI	1.9	
				2	NIIYGHGIIIFLKNVNVFPI	IIFLKNVNV	4.0	
		58	Lower probability	1	-	-	-	
				2	-	-	-	
	DRB1*13:03	18	Lower probability	1	DYVTRTSIFYHAGSFRLLTV	FYHAGSFRL	3.5	The Peptide 2 may be related to the event described for HPV-18
				2	GHYIILFLRNVNVFPIFLQM	LRNVNVFPI	4.5	
		33	Greater probability	1	EYVSRTSIYYYAGSSRLLAV	YYYAGSSRL	3.5	
				2	-	-		
		45	Greater probability	1	DYVSRTSIFYHAGSSRLLTV	FYHAGSSRL	3.0	
				2	-	-		
		58	Earlier occurrence	1	EYVSRTSIYYYAGSSRLLAV	YYYAGSSRL	3.5	
				2	-	-		
	DRB1*13:04	18	Lower probability	1	QYPLGRKFLVQAGLRRKPTI	FLVQAGLRR	1.8	
				2	-	-	-	
		31	Lower probability	1	PLGRKFLLQAGYRARPKFKA	FLLQAGYRA	2.5	
				2	TLFFYLRREQMFVRHFFNRS	LRREQMFVR	4.0	
		45	Greater probability	1	QYPLGRKFLVQAGLRRRPTI	FLVQAGLRR	1.0	
				2	-	-	-	
		58	Later occurrence	1	LGRKFLLQSGLKAKPRLKRS	FLLQSGLKA	3.0	

		58	Later occurrence	2	SLFFFLRREQMFVRHFFNRA	LRREQMFVR	3.5	No peptide explains the events found
		16	Greater probability	1	PLGRKFLLQAGLKAKPKFTL	FLLQAGLKA	1.3	
				2	SLFFYLRREQMFVRHLFNRA	FFYLRREQM	4.0	
	DRB1*13:05	18	Lower probability	1	QYPLGRKFLVQAGLRRKPTI	FLVQAGLRR	1.3	
				2	-	-	-	
		31	Later occurrence	1	GRKFLLQAGYRARPKFKAGK	FLLQAGYRA	0.9	
				2	TLFFYLRREQMFVRHFFNRS	FFYLRREQM	4.5	
		58	Lower probability	1	QYPLGRKFLVQAGLRRRPTI	FLVQAGLRR	1.0	
				2	SLFFFLRREQMFVRHFFNRA	FFFLRREQM	5.0	
	DRB1*15:03	16	Greater probability	1	QFPLGRKFLLQAGLKAKPKF	FLLQAGLKA	3.0	The Peptides 1, 2, 4 and 5 may be related to the events described for HPV-16 and 18
				2	FYLRREQMFVRHLFNRAGAV	FVRHLFNRA	4.0	
				3	EYVARTNIYYHAGTSRLLAV	YYHAGTSRL	4.0	
				4	-	-	-	
				5	-	-	-	
		18	Lower probability	1	-	-	-	
				2	-	-	-	
				3	YVTRTSIFYHAGSFRLLTVG	FYHAGSFRL	0.4	
				4	GHYIILFLRNVNVFPIFLQM	LRNVNVFPI	3.0	
				5	FYHAGSFRLLTVGNPYFRVP	LLTVGNPYF	4.5	
DQB1	DQB1*03:02	16	Greater probability	1	FPLGRKFLLQAGLKAKPKFT	FLLQAGLKA	0.5	No peptide explains the events found
				2	YVARTNIYYHAGTSRLLAVG	YYHAGTSRL	1.2	
				3	TLEDTYRFVTSQAIACQKHT	YRFVTSQAI	1.6	
				4	TGFGAMDFTTLQANKSEVPL	FTTLQANKS	2.5	
				5	TADVMTYIHSMNSTILEDWN	YIHSMNSTI	4.5	
				6	-	-	-	
		18	Lower probability	1	-	-	-	
				2	RTSIFYHAGSFRLLTVGNPY	FYHAGSFRL	0.9	
				3	TTSLVDTYRFVQSVAITCQK	YRFVQSVAI	2.0	
				4	-	-	-	
				5	TADVMSYIHSMNSSILEDWN	YIHSMNSSI	4.0	
				6	FYHAGSFRLLTVGNPYFRVP	FRLLTVGNP	5.0	
		31	Earlier occurrence	1	QFPLGRKFLLQAGYRARPKF	FLLQAGYRA	0.7	
				2	YVTRTNIYYHAGSARLLTVG	YYHAGSARL	0.4	
				3	SLEDTYRFVTSQAITCQKSA	YRFVTSQAI	3.0	
				4	-	-	-	
				5	SADIMTYIHSMNPAILEDWN	YIHSMNPAI	1.3	
				6	-	-	-	
		45	Lower probability	1	-	-	-	
				2	YVSRTSIFYHAGSSRLLTVG	FYHAGSSRL	0.5	
				3	TTSLVDTYRFVQSVAVTCQK	YRFVQSVAV	1.6	
				4	-	-	-	
				5	TAEVMSYIHSMNSSILENWN	YIHSMNSSI	3.0	
				6	-	-	-	
		58	Lower probability	1	QFPLGRKFLLQSGLKAKPRL	FLLQSGLKA	0.7	
				2	YVSRTSIYYYAGSSRLLAVG	YYYAGSSRL	0.7	
				3	ASLQDTYRFVTSQAITCQKT	YRFVTSQAI	2.5	
				4	-	-	-	
				5	-	-	-	
				6	YYYAGSSRLLAVGNPYFSIK	YYAGSSRLL	5.0	

Supplementary Table S17. MHC-II binding epitopes from the L1 protein of HR-HPV related to redetection events.

Locus	Allele	HPV	Effect on redetection	Number of peptide	Peptides	Core	%Rank	Analysis
	*DRB1*01:03*	16	Greater probability	1	QFPLGRKFLLQAGLKAKPKF	FLLQAGLKA	0.8	The peptide 4 may be related to the event described for HPV-16
				2	EYVARTNIYYHAGTSRLLAV	YYHAGTSRL	0.8	
				3	GTLEDTYRFVTSQAIACQKH	YRFVTSQAI	4.0	
				4	YLRREQMFVRHLFNRAGAVG	FVRHLFNRA	4.5	
				5	ITLTADVMTYIHSMNSTILE	YIHSMNSTI	5.0	
		18	Lower probability	1	QYPLGRKFLVQAGLRRKPTI	FLVQAGLRR	3.0	
				2	YVTRTSIFYHAGSFRLLTVG	FYHAGSFRL	0.2	
				3	TTSLVDTYRFVQSVAITCQK	YRFVQSVAI	4.0	
				4	-	-	-	
				5	ITLTADVMSYIHSMNSSILE	YIHSMNSSI	4.5	
		45	Later occurrence	1	QYPLGRKFLVQAGLRRRPTI	FLVQAGLRR	3.0	
				2	YVSRTSIFYHAGSSRLLTVG	FYHAGSSRL	0.1	
				3	TTSLVDTYRFVQSVAVTCQK	YRFVQSVAV	4.0	
				4	-	-	-	
				5	TAEVMSYIHSMNSSILENWN	YIHSMNSSI	3.0	
	*DRB1*03:02*	18	Lower probability	1	KDPYDKLKFWNVDLKEKFSL	WNVDLKEKF	7.0	No peptide explains the events found
				2	CGHYIILFLRNVNVFPIFLQ	FLRNVNVFP	8.5	
				3	-	-	-	
				4	-	-	-	
		31	Later occurrence	1	-	-	-	
				2	-	-	-	
				3	KITLSADIMTYIHSMNPAIL*	YIHSMNPAI	6.0	
				4	TYIHSMNPAILEDWNFGLTT*	ILEDWNFGL	10.0	
		45	Earlier occurrence	1	QDPYDKLKFWTVDLKEKFSS	WTVDLKEKF	9.5	
				2	GHGIIIFLKNVNVFPIFLQM	FLKNVNVFP	9.5	
				3	-	-	-	
				4	-	-	-	
		58	Later occurrence	1	-	-	-	
				2	CTLAILFCVADVNVFHIFLQ	CVADVNVFH	3.5	
				3	-	-	-	
				4	-	-	-	
	*DRB1*04:02*	18	Greater probability	1	GHYIILFLRNVNVFPIFLQM	LRNVNVFPI	4.0	Peptides 1, 2 and 3 may be related to the events described
				2	IFYHAGSFRLLTVGNPYFRV	LLTVGNPYF	4.5	
				3	-	-	-	
		58	Later occurrence	1	-	-	-	
				2	-	-	-	
				3	FPLGRKFLLQSGLKAKPRLK	FLLQSGLKA	4.0	
		16	Greater probability	1	GTLEDTYRFVTSQAIACQKH	YRFVTSQAI	1.0	
				2	LTADVMTYIHSMNSTILEDW	YIHSMNSTI	3.0	
				3	TGFGAMDFTTLQANKSEVPL	FTTLQANKS	4.0	
				4	QFPLGRKFLLQAGLKAKPKF	FLLQAGLKA	4.0	

			5	EYVARTNIYYHAGTSRLLAV	YYHAGTSRL	4.0	
			6	-	-	-	
	31	Later occurrence	1	GSLEDTYRFVTSQAITCQKS	YRFVTSQAI	1.9	
			2	LSADIMTYIHSMNPAILEDW	YIHSMNPAI	0.5	
			3	-	-	-	
			4	-	-	-	
			5	EYVTRTNIYYHAGSARLLTV	YYHAGSARL	5.0	
DRB1*04:08			6	-	-	-	The Peptide 3 may be related to the event described for HPV-16
	45	Later occurrence	1	TTSLVDTYRFVQSVAVTCQK	YRFVQSVAV	2.5	
			2	LTAEVMSYIHSMNSSILENW	YIHSMNSSI	1.7	
			3	-	-	-	
			4	-	-	-	
			5	YVSRTSIFYHAGSSRLLTVG	FYHAGSSRL	4.5	
			6	GHGIIIFLKNVNVFPIFLQM	FLKNVNVFP	3.5	
	58	Later occurrence	1	ASLQDTYRFVTSQAITCQKT	YRFVTSQAI	1.7	
			2	-	-	-	
			3	-	-	-	
			4	FPLGRKFLLQSGLKAKPRLK	FLLQSGLKA	3.5	
			5	SIYYYAGSSRLLAVGNPYFS	YYYAGSSRL	4.0	
			6	-	-	-	
DRB1*04:11	31	Earlier occurrence	1	QFPLGRKFLLQAGYRARPKF	FLLQAGYRA	3.5	No peptide explains the events found
			2	ITLSADIMTYIHSMNPAILE	YIHSMNPAI	6.0	
	45	Earlier occurrence	1	-	-	-	
			2	LTAEVMSYIHSMNSSILENW	YIHSMNSSI	4.5	
	58	Earlier occurrence	1	FPLGRKFLLQSGLKAKPRLK	FLLQSGLKA	5.0	
			2	-	-	-	
DRB1*08:01	31	Earlier occurrence	1	LGRKFLLQAGYRARPKFKAG	FLLQAGYRA	2.5	
			2	LFFYLRREQMFVRHFFNRSG	FFYLRREQM	4.0	
			3	KVSGLQYRVFRVRLPDPNKF	YRVFRVRLP	4.0	
	45	Later occurrence	1	YPLGRKFLVQAGLRRRPTIG	FLVQAGLRR	1.8	
			2	-	-	-	
			3	SAYQYRVFRVALPDPNKFGL	YRVFRVALP	2.5	
	58	Later occurrence	1	LGRKFLLQSGLKAKPRLKRS	FLLQSGLKA	3.5	
			2	LFFFLRREQMFVRHFFNRAG	FFFLRREQM	4.0	
			3	KVSGLQYRVFRVRLPDPNKF	YRVFRVRLP	4.0	
DRB1*08:04	31	Earlier occurrence	1	LGRKFLLQAGYRARPKFKAG	FLLQAGYRA	2.5	
	58	Later occurrence	1	GRKFLLQSGLKAKPRLKRSA	FLLQSGLKA	2.5	
DRB1*08:07	16	Earlier occurrence	1	-	-	-	
			2	-	-	-	
	18	Lower probability	1	GHYIILFLRNVNVFPIFLQM	LRNVNVFPI	1.3	
			2	SIFYHAGSFRLLTVGNPYFR	FYHAGSFRL	1.7	
	58	Later occurrence	1	-	-	-	
			2	-	-	-	
	18	Lower probability	1	TSIFYHAGSFRLLTVGNPYF	FYHAGSFRL	0.9	
			2	TTSLVDTYRFVQSVAITCQK	YRFVQSVAI	2.5	
			3	-	-	-	

				4	-	-	-	
				5	-	-	-	The peptides 3, 4 and 5 may be related to the event described for HPV-45
	DRB1*10:01			1	DYVSRTSIFYHAGSSRLLTV	FYHAGSSRL	2.5	
				2	TTSLVDTYRFVQSVAVTCQK	YRFVQSVAV	1.8	
DRB1		45	Earlier occurrence	3	KVSAYQYRVFRVALPDPNKF	YRVFRVALP	1.4	
				4	RLLTVGNPYFRVVPSGAGNK	YFRVVPSGA	3.0	
				5	LTAEVMSYIHSMNSSILENW	YIHSMNSSI	4.5	
	DRB1*11:01	16	Greater probability	1	PLGRKFLLQAGLKAKPKFTL	FLLQAGLKA	1.3	No peptide explains the events found
				2	SLFFYLRREQMFVRHLFNRA	FFYLRREQM	4.0	
		31	Later occurrence	1	GRKFLLQAGYRARPKFKAGK	FLLQAGYRA	0.9	
				2	TLFFYLRREQMFVRHFFNRS	FFYLRREQM	4.5	
	DRB1*11:02	31	Earlier occurrence	1	PLGRKFLLQAGYRARPKFKA	FLLQAGYRA	3.5	
		58	Later occurrence	1	LGRKFLLQSGLKAKPRLKRS	FLLQSGLKA	2.5	
	DRB1*11:04	18	Lower probability	1	YPLGRKFLVQAGLRRKPTIG	FLVQAGLRR	1.5	
		45	Earlier occurrence	1	YPLGRKFLVQAGLRRRPTIG	FLVQAGLRR	1.0	
	DRB1*11:14	31	Earlier occurrence	1	ITLSADIMTYIHSMNPAILE	YIHSMNPAI	3.0	
		45		1	VMSYIHSMNSSILENWNFGV	YIHSMNSSI	4.5	
		58		1	-	-	-	
	DRB1*12:01	18	Greater probability	1	GHYIILFLRNVNVFPIFLQM	LRNVNVFPI	2.5	The peptide 2 may be related to the event described for HPV-45
				2	-	-	-	
		31	Earlier occurrence	1	-	-	-	
				2	-	-	-	
		45	Later occurrence	1	GHGIIIFLKNVNVFPIFLQM	LKNVNVFPI	1.1	
				2	NIIYGHGIIIFLKNVNVFPI	IIFLKNVNV	4.0	
		58	Earlier occurrence	1	-	-	-	
				2	-	-	-	
	DRB1*13:03	18	Greater probability	1	DYVTRTSIFYHAGSFRLLTV	FYHAGSFRL	3.5	The peptide 2 may be related to the event described for HPV-18
				2	GHYIILFLRNVNVFPIFLQM	LRNVNVFPI	4.5	
		45	Later occurrence	1	DYVSRTSIFYHAGSSRLLTV	FYHAGSSRL	3.0	
				2	-	-	-	
		58	Later occurrence	1	EYVSRTSIYYYAGSSRLLAV	YYYAGSSRL	3.5	
				2	-	-	-	
	DRB1*13:04	18	Earlier occurrence	1	QYPLGRKFLVQAGLRRKPTI	FLVQAGLRR	1.8	No peptide explains the events found
				2	-	-	-	
		31	Earlier occurrence	1	PLGRKFLLQAGYRARPKFKA	FLLQAGYRA	2.5	
				2	TLFFYLRREQMFVRHFFNRS	LRREQMFVR	4.0	
		45	Later occurrence	1	QYPLGRKFLVQAGLRRRPTI	FLVQAGLRR	1.0	
				2	-	-	-	
		58	Earlier occurrence	1	LGRKFLLQSGLKAKPRLKRS	FLLQSGLKA	3.0	
				2	SLFFFLRREQMFVRHFFNRA	LRREQMFVR	3.5	
	DRB1*13:05	18	Greater probability	1	QYPLGRKFLVQAGLRRKPTI	FLVQAGLRR	1.3	
				2	-	-	-	
		31	Earlier occurrence	1	GRKFLLQAGYRARPKFKAGK	FLLQAGYRA	0.9	
				2	TLFFYLRREQMFVRHFFNRS	FFYLRREQM	4.5	
		45	Later occurrence	1	QYPLGRKFLVQAGLRRRPTI	FLVQAGLRR	1.0	
				2	-	-	-	

	58	Earlier occurrence	1	LGRKFLLQSGLKAKPRLKRS	FLLQSGLKA	1.2	
			2	SLFFFLRREQMFVRHFFNRA	FFFLRREQM	5.0	
	18	Greater probability	1	DYVTRTSIFYHAGSFRLLTV	FYHAGSFRL	0.7	The peptide 2 may be related to the event described for HPV-18
			2	CGHYIILFLRNVNVFPIFLQ*	LRNVNVFPI	1.1	
			3	ITLTADVMSYIHSMNSSILE	YIHSMNSSI	1.8	
			4	FYHAGSFRLLTVGNPYFRVP	LLTVGNPYF	3.5	
DRB1*15:02	31	Later occurrence	1	EYVTRTNIYYHAGSARLLTV	YYHAGSARL	1.2	
			2	-	-	-	
			3	ITLSADIMTYIHSMNPAILE	YIHSMNPAI	0.6	
			4	-	-	-	
	58	Later occurrence	1	EYVSRTSIYYYAGSSRLLAV	YYYAGSSRL	0.9	
			2	-	-	-	
			3	-	-	-	
			4	-	-	-	
	16	Earlier occurrence	1	QFPLGRKFLLQAGLKAKPKF	FLLQAGLKA	3.0	No peptide explains the events found
			2	FYLRREQMFVRHLFNRAGAV	FVRHLFNRA	4.0	
			3	EYVARTNIYYHAGTSRLLAV	YYHAGTSRL	4.0	
			4	-	-	-	
	18	Earlier occurrence	1	-	-	-	
			2	-	-	-	
			3	YVTRTSIFYHAGSFRLLTVG	FYHAGSFRL	0.4	
			4	GHYIILFLRNVNVFPIFLQM	LRNVNVFPI	3.0	
DRB1*15:03	31	Earlier occurrence	1	FPLGRKFLLQAGYRARPKFK	FLLQAGYRA	0.8	
			2	FYLRREQMFVRHFFNRSGTV	FVRHFFNRS	5.0	
			3	YVTRTNIYYHAGSARLLTVG	YYHAGSARL	1.3	
			4	-	-	-	
	45	Later occurrence	1	-	-	-	
			2	-	-	-	
			3	YVSRTSIFYHAGSSRLLTVG	FYHAGSSRL	1.2	
			4	YGHGIIIFLKNVNVFPIFLQ	LKNVNVFPI	3.5	
	58	Later occurrence	1	QFPLGRKFLLQSGLKAKPRL	FLLQSGLKA	2.5	
			2	FFLRREQMFVRHFFNRAGKL	FVRHFFNRA	1.7	
			3	EYVSRTSIYYYAGSSRLLAV	YYYAGSSRL	0.2	
			4	-	-	-	
	18	Greater probability	1	TRTSIFYHAGSFRLLTVGNP	FYHAGSFRL	0.3	
			2	QYPLGRKFLVQAGLRRKPTI	FLVQAGLRR	2.0	
			3	-	-	-	
			4	-	-	-	
	31	Earlier occurrence	1	VTRTNIYYHAGSARLLTVGH	YYHAGSARL	0.7	
			2	PLGRKFLLQAGYRARPKFKA	FLLQAGYRA	0.5	
			3	LFFYLRREQMFVRHFFNRSG	FFYLRREQM	3.5	
			4	PKVSGLQYRVFRVRLPDPNK	YRVFRVRLP	5.0	
DRB1*16:01	45	Later occurrence	1	VSRTSIFYHAGSSRLLTVGN	FYHAGSSRL	1.0	
			2	QYPLGRKFLVQAGLRRRPTI	FLVQAGLRR	1.7	
			3	-	-	-	
			4	KVSAYQYRVFRVALPDPNKF	YRVFRVALP	3.0	

		58	Later occurrence	1	VSRTSIYYYAGSSRLLAVGN	YYYAGSSRL	0.4	
				2	QFPLGRKFLLQSGLKAKPRL	FLLQSGLKA	0.6	
				3	LFFFLRREQMFVRHFFNRAG	FFFLRREQM	3.0	
				4	PKVSGLQYRVFRVRLPDPNK	YRVFRVRLP	5.0	
DQB1	***DQB1*03:02***	16	Greater probability	1	FPLGRKFLLQAGLKAKPKFT	FLLQAGLKA	0.5	Peptides 2 and 3 may be related to the events described
				2	YVARTNIYYHAGTSRLLAVG	YYHAGTSRL	1.2	
				3	TLEDTYRFVTSQAIACQKHT	YRFVTSQAI	1.6	
				4	TGFGAMDFTTLQANKSEVPL	FTTLQANKS	2.5	
				5	TADVMTYIHSMNSTILEDWN	YIHSMNSTI	4.5	
				6	-	-	-	
		45	Earlier occurrence	1	-	-	-	
				2	YVSRTSIFYHAGSSRLLTVG	FYHAGSSRL	0.5	
				3	TTSLVDTYRFVQSVAVTCQK	YRFVQSVAV	1.6	
				4	-	-	-	
				5	TAEVMSYIHSMNSSILENWN	YIHSMNSSI	3.0	
				6	-	-	-	
		58	Earlier occurrence	1	QFPLGRKFLLQSGLKAKPRL	FLLQSGLKA	0.7	
				2	YVSRTSIYYYAGSSRLLAVG	YYYAGSSRL	0.7	
				3	ASLQDTYRFVTSQAITCQKT	YRFVTSQAI	2.5	
				4	-	-	-	
				5	-	-	-	
				6	YYYAGSSRLLAVGNPYFSIK	YYAGSSRLL	5.0	
	DQB1*04:02	18	Lower probability	1	RTSIFYHAGSFRLLTVGNPY	FYHAGSFRL	0.9	No peptide explains the events found
				2	TTSLVDTYRFVQSVAITCQK	YRFVQSVAI	2.0	
				3	TADVMSYIHSMNSSILEDWN	YIHSMNSSI	4.0	
		45	Earlier occurrence	1	YVSRTSIFYHAGSSRLLTVG	FYHAGSSRL	0.5	
				2	TTSLVDTYRFVQSVAVTCQK	YRFVQSVAV	1.6	
				3	TAEVMSYIHSMNSSILENWN	YIHSMNSSI	3.0	
	DQB1*05:02	16	Earlier occurrence	1	FPLGRKFLLQAGLKAKPKFT	FLLQAGLKA	0.5	Peptides 2 and 3 may be related to the events described
				2	YVARTNIYYHAGTSRLLAVG	YYHAGTSRL	1.2	
				3	TLEDTYRFVTSQAIACQKHT	YRFVTSQAI	1.6	
				4	TGFGAMDFTTLQANKSEVPL	FTTLQANKS	2.5	
		18	Greater probability	1	-	-	-	
				2	RTSIFYHAGSFRLLTVGNPY	FYHAGSFRL	0.9	
				3	TTSLVDTYRFVQSVAITCQK	YRFVQSVAI	2.0	
				4	-	-	-	
	DQB1*06:01	18	Greater probability	1	RTSIFYHAGSFRLLTVGNPY	FYHAGSFRL	0.9	The peptide 4 may be related to the events described for HPV-31 and -58
				2	TTSLVDTYRFVQSVAITCQK	YRFVQSVAI	2.0	
				3	TADVMSYIHSMNSSILEDWN	YIHSMNSSI	4.0	
				4	-	-	-	
		31	Later occurrence	1	YVTRTNIYYHAGSARLLTVG	YYHAGSARL	0.4	
				2	SLEDTYRFVTSQAITCQKSA	YRFVTSQAI	3.0	
				3	SADIMTYIHSMNPAILEDWN	YIHSMNPAI	1.3	
				4	QFPLGRKFLLQAGYRARPKF	FLLQAGYRA	0.7	
		58	Later occurrence	1	YYYAGSSRLLAVGNPYFSIK	YYAGSSRLL	5.0	
				2	ASLQDTYRFVTSQAITCQKT	YRFVTSQAI	2.5	

[illegible]	Later occurrence	3	-	-	-
		4	QFPLGRKFLLQSGLKAKPRL	FLLQSGLKA	0.7

Supplementary Table S18. MHC-II binding epitopes from the L2 protein of HR-HPV related to clearance/persistence events.

Locus	Allele	HPV	Effect on clearance/persistence	Number of peptide	Peptide	Core	%Rank	Analysis
	*DRB1*01:03*	31	Earlier occurrence	1	GGDFYLHPSYYMLKRRRKRV	FYLHPSYYM	1.4	The peptide 2 may be related to the events described
				2	DPDFLDIIALHRPALTSRRN	IALHRPALT	3.5	
		45	Greater probability	1	-	-	-	
				2	SDFMDIIRLHRPALSSRRGT	IRLHRPALS	1.1	
	*DRB1*03:02*	16	Lower probability	1	GSPQYTIIADGGDFYLHPSY	IIADGGDFY	3.0	No peptide explains the events found
				2	AYEGIDVDNTLYVPSNDNSI	IDVDNTLYV	4.5	
				3	-	-	-	
				4	-	-	-	
				5	-	-	-	
		31	Earlier occurrence	1	-	-	-	
				2	-	-	-	
				3	-	-	-	
				4	-	-	-	
				5	-	-	-	
		33	Greater probability	1	SPFFPFDTIVVDGADFVLHP	IVVDGADFV	1.2	
				2	-	-	-	
				3	GKQIGARIHYYQDLSPIVPL	YYQDLSPIV	4.0	
				4	-	-	-	
				5	-	-	-	
		45	Lower probability	1	-	-	-	
				2	-	-	-	
				3	-	-	-	
				4	-	-	-	
				5	-	-	-	
		58	Lower probability	1	PLTPFNTIIVDGADFMLHPS	IIVDGADFM	0.9	
				2	-	-	-	
				3	-	-	-	
				4	INDGLYDIYADDADTIHDFQ	IYADDADTI	1.7	
				5	ILRRRRKRFPYFFADVRVAA	FFADVRVAA	4.5	
	*DRB1*04:08*	16	Lower probability	1	EIPMDTFIVSTNPNTVTSST	FIVSTNPNT	1.2	Peptides 1, 2, 4 and 3 may be related to the events described
				2	MLRKRRKRLPYFFSDVSLAA	FFSDVSLAA	1.6	
				3	IGAKVHYYYDLSTINPAEEI	YYYDLSTIN	4.0	
				4	AETGGHFTLSSSTISTHNYE	FTLSSSTIS	4.5	
				5	-	-	-	
		18	Greater probability	1	-	-	-	
				2	-	-	-	
				3	IGARVHFYHDISPIAPSPEY	FYHDISPIA	1.3	
				4	-	-	-	
				5	TTSFAFFKYSPTISSASSYS	FKYSPTISS	0.1	
	*DRB1*04:10*	33	Lower probability	1	-	-	-	No peptide explains the events found
		45		1	SDFMDIIRLHRPALSSRRGT	IRLHRPALS	1.2	
	*DRB1*04:11*	31	Lower probability	1	PDPDFLDIIALHRPALTSRR	IALHRPALT	3.5	
		45		1	SDFMDIIRLHRPALSSRRGT	IRLHRPALS	0.8	

DRB1		58		1	-	-	-	
	DRB1*07:11	16	Lower probability	1	QQVKVVDPAFVTTPTKLITY	FVTTPTKLI	3.0	Peptides 1 and 3 may be related to the events described
				2	GGDFYLHPSYYMLRKRRKRL	FYLHPSYYM	5.0	
				3	-	-	-	
		18	Greater probability	1	-	-	-	
				2	-	-	-	
				3	TTSFAFFKYSPTISSASSYS	FKYSPTISS	4.5	
		45	Lower probability	1	QQVRVSTSRFLTRPSSLVTF	VRVSTSRFL	4.0	
				2	-	-	-	
				3	-	-	-	
	DRB1*08:04	31	Lower probability	1	YLHPSYYMLKRRRKRVSYFF	YYMLKRRRK	0.3	No peptide explains the events found
				2	FLDIIALHRPALTSRRNTVR	IIALHRPAL	1.2	
		33	Earlier occurrence	1	VLHPSYFILRRRRKRFPYFF	YFILRRRRK	0.7	
				2	FLDIIALHRPAITSRRHTVR	IIALHRPAI	1.2	
		58	Greater probability	1	MLHPSYFILRRRRKRFPYFF	YFILRRRRK	0.7	
				2	FLDIVALHRPALTSRRGTVR	IVALHRPAL	2.5	
	DRB1*08:07	18	Earlier occurrence	1	SDFMDIIRLHRPALTSRRGT	IRLHRPALT	1.9	
		31	Greater probability	1	PDFLDIIALHRPALTSRRNT	IALHRPALT	3.0	
		33	Lower probability	1	DPDFLDIIALHRPAITSRRH	IIALHRPAI	2.5	
		45	Lower probability	1	DSDFMDIIRLHRPALSSRRG	IRLHRPALS	1.2	
		58	Earlier occurrence	1	-	-	-	
	DRB1*11:02	31	Lower probability	1	YLHPSYYMLKRRRKRVSYFF	LKRRRKRVS	1.0	The peptide 1 may be related to the clearence for HPV-31
				2	DFLDIIALHRPALTSRRNTV	IALHRPALT	1.0	
		45	Greater probability	1	-	-	-	
				2	FMDIIRLHRPALSSRRGTVR	IRLHRPALS	0.1	
	DRB1*11:14	18	Greater probability	1	-	-	-	No peptide explains the events found
				2	-	-	-	
		31	Lower probability	1	-	-	-	
				2	-	-	-	
		45	Lower probability	1	-	-	-	
				2	-	-	-	
		58	Later occurrence	1	-	-	-	
				2	-	-	-	
	DRB1*12:01	16	Greater probability	1	PDPDFLDIVALHRPALTSRR	IVALHRPAL	4.5	
		18	Lower probability	1	PDSDFMDIIRLHRPALTSRR	IIRLHRPAL	1.5	
		31	Lower probability	1	PDPDFLDIIALHRPALTSRR	IIALHRPAL	1.6	
		33	Greater probability	1	PDPDFLDIIALHRPAITSRR	IIALHRPAI	1.7	
		45	Greater probability	1	DSDFMDIIRLHRPALSSRRG	IRLHRPALS	1.1	
		58	Lower probability	1	PDPDFLDIVALHRPALTSRR	IVALHRPAL	4.5	
	DRB1*13:03	18	Lower probability	1	DSDFMDIIRLHRPALTSRRG	IRLHRPALT	3.5	
				2	-	-	-	
		33	Greater probability	1	-	-	-	
				2	GADFVLHPSYFILRRRRKRF	FVLHPSYFI	2.0	
		45	Greater probability	1	PDSDFMDIIRLHRPALSSRR	IRLHRPALS	2.5	
				2	-	-	-	
		58	Earlier occurrence	1	-	-	-	
				2	GADFMLHPSYFILRRRRKRF	FMLHPSYFI	3.0	
		18	Lower probability	1	SDFMDIIRLHRPALTSRRGT	IRLHRPALT	0.4	

		18	Lower probability	2	YLWPLYYFIPKKRKRVPYFF	LYYFIPKKR	1.7	
		31	Lower probability	1	DPDFLDIIALHRPALTSRRN	IIALHRPAL	1.5	
				2	YLHPSYYMLKRRRKRVSYFF	LKRRRKRVS	0.4	The peptide 2 may be related to the clearence for HPV-18, -31 and -58
	DRB1*13:04	45	Greater probability	1	SDFMDIIRLHRPALSSRRGT	IRLHRPALS	0.2	
				2	-	-	-	
		58	Later occurrence	1	FLDIVALHRPALTSRRGTVR	IVALHRPAL	3.5	
				2	FMLHPSYFILRRRRKRFPYF	ILRRRRKRF	0.4	
		16	Greater probability	1	DFYLHPSYYMLRKRRKRLPY	YYMLRKRRK	0.1	
				2	PDFLDIVALHRPALTSRRTG	IVALHRPAL	5.0	
		18	Lower probability	1	LWPLYYFIPKKRKRVPYFFA	YYFIPKKRK	0.3	
				2	SDFMDIIRLHRPALTSRRGT	IIRLHRPAL	1.3	
	DRB1*13:05	31	Later occurrence	1	YLHPSYYMLKRRRKRVSYFF	YYMLKRRRK	0.05	No peptide explains the events found
				2	DPDFLDIIALHRPALTSRRN	IIALHRPAL	3.5	
		58	Lower probability	1	MLHPSYFILRRRRKRFPYFF	YFILRRRRK	0.2	
				2	DPDFLDIVALHRPALTSRRG	IVALHRPAL	5.0	
		16	Greater probability	1	GGDFYLHPSYYMLRKRRKRL	FYLHPSYYM	0.8	
				2	-	-	-	
				3	-	-	-	
	DRB1*15:03	18	Lower probability	1	GTHYYLWPLYYFIPKKRKRV	LYYFIPKKR	1.8	Peptides 2 and 3 may be related to the clearence for HPV-18
				2	DSDFMDIIRLHRPALTSRRG	IRLHRPALT	1.7	
				3	RSTTSFAFFKYSPTISSASS	FFKYSPTIS	3.0	
		16	Greater probability	1	GSPQYTIIADGGDFYLHPSY	IIADGGDFY	3.0	
				2	AYEGIDVDNTLYVPSNDNSI	IDVDNTLYV	4.5	
				3	-	-	-	
				4	-	-	-	
		18	Lower probability	1	-	-	-	
				2	-	-	-	
				3	-	-	-	
				4	-	-	-	
DQB1	*DQB1*03:02*	31	Earlier occurrence	1	-	-	-	No peptide explains the events found
				2	-	-	-	
				3	-	-	-	
				4	-	-	-	
		45	Lower probability	1	-	-	-	
				2	-	-	-	
				3	-	-	-	
				4	-	-	-	
		58	Lower probability	1	PLTPFNTIIVDGADFMLHPS	IIVDGADFM	0.9	
				2	-	-	-	
				3	INDGLYDIYADDADTIHDFQ	IYADDADTI	1.7	
				4	ILRRRRKRFPYFFADVRVAA	FFADVRVAA	4.5	

Supplementary Table S19. MHC-II binding epitopes from the L2 protein of HR-HPV related to redetection events.

Locus	Allele	HPV	Effect on redetection	Number of peptide	Peptide	Core	%Rank	Analysis
	*DRB1*01:03*	16	Greater probability	1	GGDFYLHPSYYMLRKRRKRL	FYLHPSYYM	1.1	The peptide 2 may be related to the redetection for HPV-18 and -45
				2	-	-	-	
				3	-	-	-	
		18	Lower probability	1	-			
				2	SDFMDIIRLHRPALTSRRGT	IRLHRPALT	1.4	
				3	TTSFAFFKYSPTISSASSYS	FKYSPTISS	3.5	
				4				
				5				
		45	Later occurrence	1	-			
				2	SDFMDIIRLHRPALSSRRGT	IRLHRPALS	1.1	
				3	-	-	-	
	*DRB1*03:02*	18	Lower probability	1	-	-	-	The peptide 1 may be related to the redetection for HPV-45
				2	-	-	-	
				3	-	-	-	
				4	-	-	-	
		31	Later occurrence	1	-	-	-	
				2	-	-	-	
				3	-	-	-	
				4	-	-	-	
		45	Earlier occurrence	1	QIGGRVHFYHDISPIAATEE	FYHDISPIA	4.5	
				2	-	-	-	
				3	-	-	-	
				4	-	-	-	
		58	Later occurrence	1	-	-	-	
				2	PLTPFNTIIVDGADFMLHPS	IIVDGADFM	0.9	
				3	INDGLYDIYADDADTIHDFQ	IYADDADTI	1.7	
				4	ILRRRRKRFPYFFADVRVAA	FFADVRVAA	4.5	
	*DRB1*04:02*	18	Greater probability	1	SFAFFKYSPTISSASSYSNV	FKYSPTISS	1.7	Peptides 1 and 2 may be related to the redetection for HPV-18
				2	SDFMDIIRLHRPALTSRRGT	IRLHRPALT	4.0	
		58	Later occurrence	1	-	-	-	
				2	-	-	-	
	*DRB1*04:08*	16	Greater probability	1	EIPMDTFIVSTNPNTVTSST	FIVSTNPNT	1.2	Peptide 4 may be related to the redetection for HPV-16
				2	MLRKRRKRLPYFFSDVSLAA	FFSDVSLAA	1.6	
				3	IGAKVHYYYDLSTINPAEEI	YYYDLSTIN	4.0	
				4	AETGGHFTLSSSTISTHNYE	FTLSSSTIS	4.5	
		31	Later occurrence	1	EIPMDTFIVSTNNENITSST	FIVSTNNEN	4.0	
				2	MLKRRRKRVSYFFTDVSVAA	FFTDVSVAA	3.5	
				3	GARVHYYYDISSINPAGESI	YYYDISSIN	3.5	
				4	-	-	-	
		45	Later occurrence	1	-	-	-	
				2	-	-	-	
				3	IGGRVHFYHDISPIAATEEI	-	0.7	

				4	-	-	-	
		58	Later occurrence	1	-	-	-	
				2	-	-	-	
				3	-	-	-	
				4	-	-	-	
	DRB1*04:11	31	Earlier occurrence	1	PDPDFLDIIALHRPALTSRR	IALHRPALT	3.5	
		45	Earlier occurrence	1	SDFMDIIRLHRPALSSRRGT	IRLHRPALS	0.8	
		58	Earlier occurrence	1	-	-	-	
	DRB1*08:01	31	Earlier occurrence	1	FYLHPSYYMLKRRRKRVSYF	YYMLKRRRK	0.6	
				2	PDFLDIIALHRPALTSRRNT	IIALHRPAL	2.5	
		45	Later occurrence	1	-	-	-	
				2	SDFMDIIRLHRPALSSRRGT	IRLHRPALS	0.4	
		58	Later occurrence	1	FMLHPSYFILRRRRKRFPYF	YFILRRRRK	1.1	No peptide explains the events found
				2	PDFLDIVALHRPALTSRRGT	IVALHRPAL	4.0	
	DRB1*08:04	31	Earlier occurrence	1	YLHPSYYMLKRRRKRVSYFF	YYMLKRRRK	0.3	
				2	FLDIIALHRPALTSRRNTVR	IIALHRPAL	1.2	
		58	Later occurrence	1	MLHPSYFILRRRRKRFPYFF	YFILRRRRK	0.7	
				2	FLDIVALHRPALTSRRGTVR	IVALHRPAL	2.5	
	DRB1*08:07	16	Earlier occurrence	1	-	-	-	
		18	Lower probability	1	SDFMDIIRLHRPALTSRRGT	IRLHRPALT	1.9	
		58	Later occurrence	1	-	-	-	
	DRB1*10:01	18	Lower probability	1	TTSFAFFKYSPTISSASSYS	FKYSPTISS	0.2	The peptide 1 may be related to the redetection for HPV-18
		45	Earlier occurrence	1	-	-	-	
	DRB1*11:01	16	Greater probability	1	DFYLHPSYYMLRKRRKRLPY	YYMLRKRRK	0.1	
				2	PDFLDIVALHRPALTSRRTG	IVALHRPAL	5.0	
		31	Later occurrence	1	YLHPSYYMLKRRRKRVSYFF	YYMLKRRRK	0.05	
				2	DPDFLDIIALHRPALTSRRN	IIALHRPAL	3.5	
	DRB1*11:02	31	Earlier occurrence	1	YLHPSYYMLKRRRKRVSYFF	LKRRRKRVS	1.0	
				2	DFLDIIALHRPALTSRRNTV	IALHRPALT	1.0	
		58	Later occurrence	1	FMLHPSYFILRRRRKRFPYF	ILRRRRKRF	1.8	
				2	FLDIVALHRPALTSRRGTVR	IVALHRPAL	2.5	
DRB1	***DRB1*11:04***	18	Lower probability	1	LWPLYYFIPKKRKRVPYFFA	YYFIPKKRK	0.1	
				2	SDFMDIIRLHRPALTSRRGT	IIRLHRPAL	1.0	
		45	Earlier occurrence	1	LWPWYYYFPKKRKRIPYFFA	YFPKKRKRI	2.0	
				2	SDFMDIIRLHRPALSSRRGT	IRLHRPALS	0.7	
	DRB1*11:14	31	Earlier occurrence	1	-	-	-	No peptide explains the events found
		45		1	-	-	-	
		58		1	-	-	-	
	DRB1*12:01	18	Greater probability	1	PDSDFMDIIRLHRPALTSRR	IIRLHRPAL	1.5	
		31	Earlier occurrence	1	PDPDFLDIIALHRPALTSRR	IIALHRPAL	1.6	
		45	Later occurrence	1	DSDFMDIIRLHRPALSSRRG	IRLHRPALS	1.1	
		58	Earlier occurrence	1	PDPDFLDIVALHRPALTSRR	IVALHRPAL	4.5	
	DRB1*13:03	18	Greater probability	1	DSDFMDIIRLHRPALTSRRG	IRLHRPALT	3.5	
				2	-	-	-	
		45	Later occurrence	1	PDSDFMDIIRLHRPALSSRR	IRLHRPALS	2.5	
				2	-	-	-	
		58	Later occurrence	1	-	-	-	

	58	Later occurrence	2	GADFMLHPSYFILRRRRKRF	FMLHPSYFI	3.0	
*DRB1*13:04*	18	Earlier occurrence	1	SDFMDIIRLHRPALTSRRGT	IRLHRPALT	0.4	The peptide 2 may be related to the redetection for HPV-18, -31 and -58
			2	YLWPLYYFIPKKRKRVPYFF	LYYFIPKKR	1.7	
	31	Earlier occurrence	1	DPDFLDIIALHRPALTSRRN	IIALHRPAL	1.5	
			2	YLHPSYYMLKRRRKRVSYFF	LKRRRKRVS	0.4	
	45	Later occurrence	1	SDFMDIIRLHRPALSSRRGT	IRLHRPALS	0.25	
			2	-	-	-	
	58	Earlier occurrence	1	FLDIVALHRPALTSRRGTVR	IVALHRPAL	3.5	
			2	FMLHPSYFILRRRRKRFPYF	ILRRRRKRF	0.4	
*DRB1*13:05*	18	Greater probability	1	LWPLYYFIPKKRKRVPYFFA	YYFIPKKRK	0.3	No peptide explains the events found
			2	SDFMDIIRLHRPALTSRRGT	IIRLHRPAL	1.3	
	31	Earlier occurrence	1	YLHPSYYMLKRRRKRVSYFF	YYMLKRRRK	0.05	
			2	DPDFLDIIALHRPALTSRRN	IIALHRPAL	3.5	
	45	Later occurrence	1	LWPWYYYFPKKRKRIPYFFA	YFPKKRKRI	3.5	
			2	SDFMDIIRLHRPALSSRRGT	IRLHRPALS	0.8	
	58	Earlier occurrence	1	MLHPSYFILRRRRKRFPYFF	YFILRRRRK	0.2	
			2	DPDFLDIVALHRPALTSRRG	IVALHRPAL	5.0	
*DRB1*15:02*	18	Greater probability	1	STTSFAFFKYSPTISSASSY	FKYSPTISS	4.0	Peptides 1, 2 and 3 may be related to the event described
			2	IGARVHFYHDISPIAPSPEY	FYHDISPIA	4.0	
			3	-	-	-	
	31	Later occurrence	1	-	-	-	
			2	-	-	-	
			3	SIFVDGGDFYLHPSYYMLKR	FYLHPSYYM	3.5	
	58	Later occurrence	1	-	-	-	
			2	-	-	-	
			3	TIIVDGADFMLHPSYFILRR	FMLHPSYFI	2.0	
*DRB1*15:03*	16	Earlier occurrence	1	GGDFYLHPSYYMLRKRRKRL	FYLHPSYYM	0.8	
			2	-	-	-	
			3	-	-	-	
			4	-	-	-	
	18	Earlier occurrence	1	GTHYYLWPLYYFIPKKRKRV	LYYFIPKKR	1.8	
			2	DSDFMDIIRLHRPALTSRRG	IRLHRPALT	1.7	
			3	RSTTSFAFFKYSPTISSASS	FFKYSPTIS	3.0	
			4				
	31	Earlier occurrence	1	GGDFYLHPSYYMLKRRRKRV	FYLHPSYYM	0.8	
			2	DPDFLDIIALHRPALTSRRN	IALHRPALT	2.5	
			3	-	-	-	
			4	TTIADQILRYGSMGVFFGGL	ILRYGSMGV	4.5	
	45	Later occurrence	1	-	-	-	
			2	DSDFMDIIRLHRPALSSRRG	IRLHRPALS	1.5	
			3	-	-	-	
			4	-	-	-	
	58	Later occurrence	1	GADFMLHPSYFILRRRRKRF	FMLHPSYFI	0.9	
			2	-	-	-	
			3	-	-	-	
			4	TTIADQILRYGSLGVFFGGL	ILRYGSLGV	4.5	
			1	HYYLWPLYYFIPKKRKRVPY	LYYFIPKKR	0.8	

		18	Greater probability	2	STTSFAFFKYSPTISSASSY	FKYSPTISS	1.0	No peptide explains the events found
				3	SDFMDIIRLHRPALTSRRGT	IRLHRPALT	4.5	
		31	Earlier occurrence	1	GGDFYLHPSYYMLKRRRKRV	YYMLKRRRK	0.3	
				2	-	-	-	
				3	-	-	-	
	*DRB1*16:01*	45	Later occurrence	1	YYLWPWYYYFPKKRKRIPYF	WYYYFPKKR	2.0	
				2	-	-	-	
				3	SDFMDIIRLHRPALSSRRGT	IRLHRPALS	3.0	
		58	Later occurrence	1	GADFMLHPSYFILRRRRKRF	YFILRRRRK	1.0	
				2	-	-	-	
				3	-	-	-	
		16	Greater probability	1	GSPQYTIIADGGDFYLHPSY	IIADGGDFY	3.0	
				2	AYEGIDVDNTLYVPSNDNSI	IDVDNTLYV	4.5	
				3	-	-	-	
				4	-	-	-	
	*DQB1*03:02*	45	Earlier occurrence	1	-	-	-	
				2	-	-	-	
				3	-	-	-	
				4	-	-	-	
DQB1		58	Earlier occurrence	1	PLTPFNTIIVDGADFMLHPS	IIVDGADFM	0.9	
				2	-	-	-	
				3	INDGLYDIYADDADTIHDFQ	IYADDADTI	1.7	
				4	ILRRRRKRFPYFFADVRVAA	FFADVRVAA	4.5	
	*DQB1*04:02*	18	Lower probability	1	TTSFAFFKYSPTISSASSYS	FKYSPTISS	1.8	Peptides may be related to the event described
		45	Earlier occurrence	1	SDFMDIIRLHRPALSSRRGT	IRLHRPALS	4.5	
	*DQB1*05:02*	16	Earlier occurrence	1	-	-	-	No peptide explains the events found
		18	Greater probability	1	TTSFAFFKYSPTISSASSYS	FKYSPTISS	1.8	
	*DQB1*06:01*	18	Greater probability	1	TTSFAFFKYSPTISSASSYS	FKYSPTISS	1.8	
		31	Later occurrence	1	-	-	-	
		58	Later occurrence	1	-	-	-	

Supplementary Table 20. Oligonucleotide primers used in this study

Gen	Viral type	Cebador	Primer sequence (5' - 3')	Size (bp)
L1- GP5+/6+	HPV	Fwd	TTTGTTACTGTGGTAGATACTAC	150
		Rev	GAAAAATAAACTGTAAATCATATTC	
L1- MY09/11		Fwd	CGTCCMARRGGAWACTGATC	450
		Rev	GCMCAGGGWCATAAYAATGG	
E6	HPV-16	Fwd	TCAAAAGCCACTGTGTCCTGA	120
		Rev	CGTGTTCTTGATGATCTGCAA	
E6-E7	HPV -18	Fwd	GACAGGAACGACTCCAACGA	202
		Rev	GCTGGTAAATGTTGATGATTAACT	
E5	HPV -31	Fwd	CTACAGTAAGCATTGTGCTAT	155
		Rev	ACGTAATGGAGAGGTTGCAATAACCC	
E7	HPV -33	Fwd	AACGCCATGAGAGGACACAAG	212
		Rev	ACACATAAACGAACTGTGGTG	
E6-E7	HPV -45	Fwd	ACGGCAAGAAAGACTTCGCA	134
		Rev	CACAACAGGTCAACAGGATC	
E7	HPV -58	Fwd	CGAGGATGAAATAGGCTTGG	109
		Rev	ACACAAACGAACCGTGGTGC	

Fwd: forward; Rev: reverse; HPV: human papillomavirus; bp: base pairs

Supplementary Table 21. qPCR oligonucleotide primers and probes

Gene	Viral type	Primer	Primer sequence (5' - 3')	Fluorophore	Quencher
E7	HPV-16	Fwd<br>Rev<br>Prb	AGCTCAGAGGAGGAGGAT<br>GGTTACAATATTGTAATGGGCTC<br>CCAGCTGGACAAGCAGAACCGG	FAM	ZEN/IBFQ
E1	HPV -18	Fwd<br>Rev<br>Prb	CATTTTGTGAACAGGCAGAGC<br>ACTTGTGCATCATTGTGGACC<br>AGAGACAGCACAGGCATTGTTCCATG	Cy5	IBRQ
E6	HPV -31	Fwd<br>Rev<br>Prb	ACGATTCCACAACATAGGAGGA<br>TACACTTGGGTTTCAGTACGAGGT<br>GGACGTTGCATAGCATGTTGGAG	HEX	ZEN/IBFQ
E7	HPV -33	Fwd<br>Rev<br>Prb	ATTAAGTGACAGCTCAGATGA<br>ACATAAACGAACTGTGGTGTT<br>ACCAGCCACAGCTGATTACTACATTGTA	FAM	ZEN/IBFQ
E1	HPV -45	Fwd<br>Rev<br>Prb	CCATTTGTGAACAGGCAGAGC<br>CAACACCTGTGCATCATTCTGA<br>AGAGACAGCACAGGCATTGTTCCATG	Cy5	IBRQ
E7	HPV -58	Fwd<br>Rev<br>Prb	CGAGGATGAAATAGGCTTGG<br>ACACAAACGAACCGTGGCGT<br>CGGCCACAGCTAATTACTACATTGT	HEX	ZEN/IBFQ
HMBS		Fwd<br>Rev<br>Prb	GCCTGCAGTTTGAAATCAGTG<br>CGGGACGGGCTTTAGCTA<br>TGGAAGCTAATGGGAAGCCCAGTACC	FAM	ZEN/IBFQ

Fwd: forward; Rev: reverse; Prb: probe; HPV: human papillomavirus; *HMBS: hydroxymethylbilane synthase*; FAM (6-carboxyfluorescein); Cy5: FluoroLink; mono reactive dye Cy5; HEX: hexachlorofluoresceine; ZEN/IBFQ: ZEN and Iowa Black FQ; IBRQ: Iowa Black RQ.

References

1. Maciag PC, Schlecht NF, Souza PS, Rohan TE, Franco EL, Villa LL. Polymorphisms of the human leukocyte antigen DRB1 and DQB1 genes and the natural history of human papillomavirus infection. J Infect Dis **2002**; 186:164-72.

2. Zoodsma M, Nolte IM, Te Meerman GJ, De Vries EG, Van der Zee AG. HLA genes and other candidate genes involved in susceptibility for (pre)neoplastic cervical disease. Int J Oncol **2005**; 26:769-84.

3. Zhang X, Zhang L, Tian C, Yang L, Wang Z. Genetic variants and risk of cervical cancer: epidemiological evidence, meta-analysis and research review. BJOG **2014**; 121:664-74.

4. Wei LZ, Wang HL, Liu X, et al. Meta-analysis on the relationship between HLA-DRBl gene polymorphism and cervical cancer in Chinese population. PLoS One **2014**; 9:e88439.

5. Bernal-Silva S, Granados J, Gorodezky C, et al. HLA-DRB1 Class II antigen level alleles are associated with persistent HPV infection in Mexican women; a pilot study. Infect Agent Cancer **2013**; 8:31.

6. Chuang LC, Hu CY, Chen HC, et al. Associations of human leukocyte antigen class II genotypes with human papillomavirus 18 infection and cervical intraepithelial neoplasia risk. Cancer **2012**; 118:223-31.

7. Maciag PC, Schlecht NF, Souza PS, Franco EL, Villa LL, Petzl-Erler ML. Major histocompatibility complex class II polymorphisms and risk of cervical cancer and human papillomavirus infection in Brazilian women. Cancer Epidemiol Biomarkers Prev **2000**; 9:1183-91.

8. Yang YC, Chang TY, Lee YJ, et al. HLA-DRB1 alleles and cervical squamous cell carcinoma: experimental study and meta-analysis. Hum Immunol **2006**; 67:331-40.

9. Xiao X, Liu L, Li WJ, Liu J, Chen DJ. HLA-A, HLA-B, HLA-DRB1 polymorphisms and risk of cervical squamous epithelial cell carcinoma: a population study in China. Asian Pac J Cancer Prev **2013**; 14:4427-33.

10. Wu Y, Liu B, Lin W, et al. Human leukocyte antigen class II alleles and risk of cervical cancer in China. Hum Immunol **2007**; 68:192-200.

11. Liang J, Xu A, Xie Y, Awonuga AO, Lin Z. Some but not all of HLA-II alleles are associated with cervical cancer in Chinese women. Cancer Genet Cytogenet **2008**; 187:95-100.

12. Wang SS, Wheeler CM, Hildesheim A, et al. Human leukocyte antigen class I and II alleles and risk of cervical neoplasia: results from a population-based study in Costa Rica. J Infect Dis **2001**; 184:1310-4.

13. Eiguchi K, Tatti S, Alonio LV, et al. Association of DRB1 and DQB1 HLA class II polymorphisms in high-grade and neoplastic cervical lesions of women from Argentina. J Low Genit Tract Dis **2008**; 12:262-8.

14. Madeleine MM, Johnson LG, Smith AG, et al. Comprehensive analysis of HLA-A, HLA-B, HLA-C, HLA-DRB1, and HLA-DQB1 loci and squamous cell cervical cancer risk. Cancer Res **2008**; 68:3532-9.

15. Beskow AH, Josefsson AM, Gyllensten UB. HLA class II alleles associated with infection by HPV16 in cervical cancer in situ. Int J Cancer **2001**; 93:817-22.

16. Schiff MA, Apple RJ, Lin P, Nelson JL, Wheeler CM, Becker TM. HLA alleles and risk of cervical intraepithelial neoplasia among southwestern American Indian women. Hum Immunol **2005**; 66:1050-6.
